# Nanobodies dismantle post‐pyroptotic ASC specks and counteract inflammation *in vivo*

**DOI:** 10.15252/emmm.202115415

**Published:** 2022-04-19

**Authors:** Damien Bertheloot, Carlos WS Wanderley, Ayda H Schneider, Lisa DJ Schiffelers, Jennifer D Wuerth, Jan MP Tödtmann, Salie Maasewerd, Ibrahim Hawwari, Fraser Duthie, Cornelia Rohland, Lucas S Ribeiro, Lea‐Marie Jenster, Nathalia Rosero, Yonas M Tesfamariam, Fernando Q Cunha, Florian I Schmidt, Bernardo S Franklin

**Affiliations:** ^1^ Institute of Innate Immunity Medical Faculty University of Bonn Bonn Germany; ^2^ Center for Research in Inflammatory Diseases (CRID) Ribeirao Preto Medical School University of Sao Paulo Sao Paulo Brazil; ^3^ Department of Pharmacology Ribeirao Preto Medical School University of Sao Paulo Sao Paulo Brazil; ^4^ Core Facility Nanobodies Medical Faculty University of Bonn Bonn Germany

**Keywords:** arthritis, extracellular inflammasomes, gout, nanobodies, pyroptosis, Immunology, Musculoskeletal System

## Abstract

Inflammasomes sense intracellular clues of infection, damage, or metabolic imbalances. Activated inflammasome sensors polymerize the adaptor ASC into micron‐sized “specks” to maximize caspase‐1 activation and the maturation of IL‐1 cytokines. Caspase‐1 also drives pyroptosis, a lytic cell death characterized by leakage of intracellular content to the extracellular space. ASC specks are released among cytosolic content, and accumulate in tissues of patients with chronic inflammation. However, if extracellular ASC specks contribute to disease, or are merely inert remnants of cell death remains unknown. Here, we show that camelid‐derived nanobodies against ASC (VHH_ASC_) target and disassemble post‐pyroptotic inflammasomes, neutralizing their prionoid, and inflammatory functions. Notably, pyroptosis‐driven membrane perforation and exposure of ASC specks to the extracellular environment allowed VHH_ASC_ to target inflammasomes while preserving pre‐pyroptotic IL‐1β release, essential to host defense. Systemically administrated mouse‐specific VHH_ASC_ attenuated inflammation and clinical gout, and antigen‐induced arthritis disease. Hence, VHH_ASC_ neutralized post‐pyroptotic inflammasomes revealing a previously unappreciated role for these complexes in disease. VHH_ASC_ are the first biologicals that disassemble pre‐formed inflammasomes while preserving their functions in host defense.

## Graphical abstract

Inflammasome activation results in a lytic cell death named Pyroptosis. Following pyroptosis, inflammasome platforms called ASC specks are released into the extracellular space, where they spread inflammation through their pro‐inflammatory and prion‐like activities. However, if extracellular ASC specks have functions in the development of disease remains ill‐defined. The present study tackles this question using ASC‐targeting nanobodies.

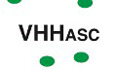

## Introduction

Inflammasomes are supramolecular organizing centers (SMOCs) that enable maturation of IL‐1‐family cytokines (e.g., IL‐1β) and trigger a pro‐inflammatory form of lytic cell death, termed pyroptosis. Inflammasomes are assembled following the activation of a sensor molecule which nucleates the oligomerization of the adaptor protein apoptosis‐associated speck‐like protein containing a CARD (ASC) (Martinon *et al*, 2002). ASC assembles into filamentous structures that are cross‐linked into star‐shaped complexes called specks, and which optimize signal transduction via proximity‐induced caspase‐1 activation. Caspase‐1 then drives the maturation of pro‐forms of IL‐1 cytokines and the proteolytic activation of gasdermin D (GSDMD). Active GSDMD oligomerizes and forms pores in the plasma membrane, which enable the release of IL‐1β and induce pyroptosis. At a later stage of pyroptosis, membrane damage is extensive andallows for the release of larger endogenous molecules including ASC specks (Baroja‐Mazo *et al*, 2014; Franklin *et al*, 2014; Volchuk *et al*, 2020).

Although IL‐1 cytokines are crucial to counter infection, their unbalanced production is responsible for a large spectrum of autoinflammatory disorders (Broderick *et al*, 2015; Spel & Martinon, 2020; Tartey & Kanneganti, 2020). Therapeutic strategies developed to prevent the negative effect of aberrant IL‐1 cytokines have mainly focused on targeting either cytokines (especially IL‐1β) or their receptors directly (Chauhan *et al*, 2020). However, the outputs of inflammasome activation are broader and include numerous danger signals (DAMPS), lipid mediators (von Moltke *et al*, 2012), and alarmins (Phulphagar *et al*, 2021). Given their essential roles in host defense, continuous inhibition of IL‐1 cytokines increases the risk of infections, as observed in patients receiving anakinra (Salliot *et al*, 2009). Hence, interest in targeting specific inflammasome sensors (e.g., NLRP3) has risen in the last years (Mangan *et al*, 2018; Chauhan *et al*, 2020). Preventing the activation of the upstream sensor would shortcut the cell death pathway triggered by a specific inflammasome and the ensuing release of DAMPs and ASC specks. However, this would also prevent beneficial roles of NLRP3 in host defense.

In addition to its central role as an inflammasome adaptor, inflammasome‐independent functions of ASC that amplify inflammation have been described (Ellebedy *et al*, 2011; Guo & Dhodapkar, 2012; Tsuchiya *et al*, 2014; Franklin *et al*, 2017; Venegas *et al*, 2017; Friker *et al*, 2020). We and others have shown that ASC specks are released from pyroptotic cells into the extracellular space (Baroja‐Mazo *et al*, 2014; Franklin *et al*, 2014). Once released, these post‐pyroptotic specks accumulate in tissues, where they sustain production of IL‐1β or are engulfed by surrounding phagocytes to propagate inflammation through seeding of further inflammasome structures within the recipient cells. Extracellular ASC specks are observed in mice after viral or bacterial infection (Sagoo *et al*, 2016; Tzeng *et al*, 2016), in the serum of patients with cryopyrin‐associated periodic syndrome (CAPS) (Baroja‐Mazo *et al*, 2014) or HIV infection (Ahmad *et al*, 2018), or in the bronchoalveolar lavage of chronic obstructive pulmonary disease (COPD) patients (Franklin *et al*, 2014). Recently, ASC specks were suggested as candidate biomarkers for medullary pyroptosis and ineffective hemopoiesis in patients with myelodysplastic syndromes (Basiorka *et al*, 2018). In fact, ASC‐targeting antibodies develop in patients with autoimmune disease (Franklin *et al*, 2014) indicating exposure of antigen‐presenting cells to circulating ASC. Furthermore, ASC^PYD^ has prion‐like activities *in vitro* and in yeast (Cai *et al*, 2014; Lu *et al*, 2014) that are maintained in mammalian cells, where engulfed ASC specks nucleate the polymerization of soluble ASC (Franklin *et al*, 2014). In the brain, ASC specks from pyroptotic microglia are released into the parenchyma where they cross‐seed Amyloid‐β plaques and contribute to neuroinflammation in Alzheimer's Disease (AD; Venegas *et al*, 2017; Friker *et al*, 2020). Hence, there is high interest in targeting ASC specks to limit their extracellular pro‐inflammatory activity.

Our previous attempt to target ASC specks using conventional antibodies (Abs) resulted in increased inflammation in a silica‐induced model of peritonitis (Franklin *et al*, 2014). Anti‐ASC Abs promoted the uptake of extracellular ASC specks by phagocytes through Fc‐mediated opsonization leading to increased IL‐1β release from macrophages and immune cell infiltration into the peritoneal cavity. This common feature of conventional Abs used for therapy encouraged the development of alternative approaches, including single‐domain antibody fragments, such as nanobodies (VHHs), which are derived from larger heavy chain‐only Abs found in camelids. We recently generated a VHH against human ASC (VHH_ASC_), which we over‐expressed in the cytosol of cells to study the molecular mechanisms involved in ASC oligomerization (Schmidt *et al*, 2016b). We showed that VHH_ASC_ binds the caspase‐recruitment domain (ASC^CARD^) of ASC, preventing formation of CARD/CARD interactions necessary to form full ASC specks.

In this study, we tested the therapeutic potential of VHH_ASC_ and a newly generated VHH against murine ASC (VHH_mASC_) to target ASC specks *in vitro* and *in vivo*. We show that pre‐incubation of extracellular ASC specks with VHH_ASC_ abrogated their inflammatory functions *in vitro*. Recombinant VHH_ASC_ rapidly disassembled pre‐formed ASC specks and thus inhibited their ability to seed the nucleation of soluble ASC. Notably, VHH_ASC_ required prior cytosolic access to prevent inflammasome activation within cells, but it was effective against extracellular ASC specks released following caspase‐1‐dependent loss of membrane integrity, and pyroptosis. Finally, systemic treatment with VHH_mASC_ efficiently dampened the inflammation induced by intra‐articular challenges with either monosodium urate (MSU) crystals or mBSA in mouse models of acute gout or chronic rheumatoid arthritis (RA), respectively.

Together, our data provide the first evidence that extracellular ASC specks, or those exposed to the extracellular environment following pyroptosis, contribute to the development of chronic disease such as gout and rheumatoid arthritis. They also indicate that VHH_ASC_ can effectively disassemble pre‐formed inflammasomes, thus questioning for the first time the stabile nature of SMOCs. Hence, our study opens the possibility of targeting inflammasomes, and potentially other SMOCs, in established chronic inflammatory diseases in the clinic.

## Results

### VHH_ASC_ blocks pro‐inflammatory function of extracellular ASC specks

**Figure 1:**
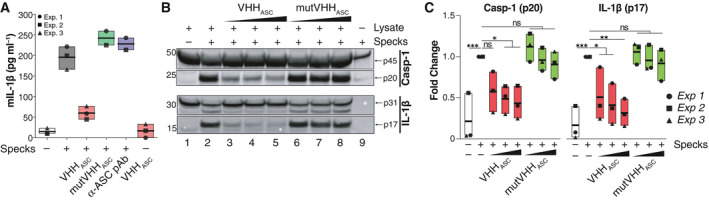
VHH_ASC_ blocks the extracellular pro‐inflammatory function of ASC specks Mouse IL‐1β (mIL‐1β) concentrations in cell‐free supernatants of LPS‐primed BMDMs (200 ng ml^−1^, for 2 h), that were stimulated with *in vitro*‐generated human ASC specks (200 µg ml^−1^, O/N). ASC specks were pre‐incubated with 200 µg ml^−1^ of an anti‐human ASC nanobody (VHH_ASC_), its mutated variant, mutVHH_ASC_ or 50 µg ml^−1^ of polyclonal ASC antibody (a‐ASC pAb, AL177) for 15 min at RT. Data is pooled from at least two independent experiments, each represented by a different symbol, and is displayed as floating bars with the max/min values and mean (thicker band).Immunoblot analysis of pro‐ (p31) and cleaved (p17) IL‐1β, and pro‐ (p45) and cleaved (p20) caspase‐1 in cytosolic fractions of ASC deficient (*Pycard* ^−/−^) immortalized murine macrophages that were primed with LPS (200 ng ml^−1^, for 3 h). Fractions were incubated with recombinant ASC specks in the presence of 2, 20 or 200 µg ml^−1^ of VHH_ASC_ or mutVHH_ASC_. One representative of three independent experiments is shown.Quantitative analysis of band densitometry of three independent experiments as shown in B. Data represents the fold change from all conditions (lanes 1–9) vs. *Pycard* ^−/−^ lysates incubated with ASC specks alone (lane 2). ^ns^ *P* > 0.05; *P < 0.05; **P < 0.005; ***P < 0.0002, One‐way ANOVA, multiple comparison (Dunnet test). Data is displayed as floating bars with the max/min values and mean (thicker band). Source data are available online for this figure.

**Figure EV1:**
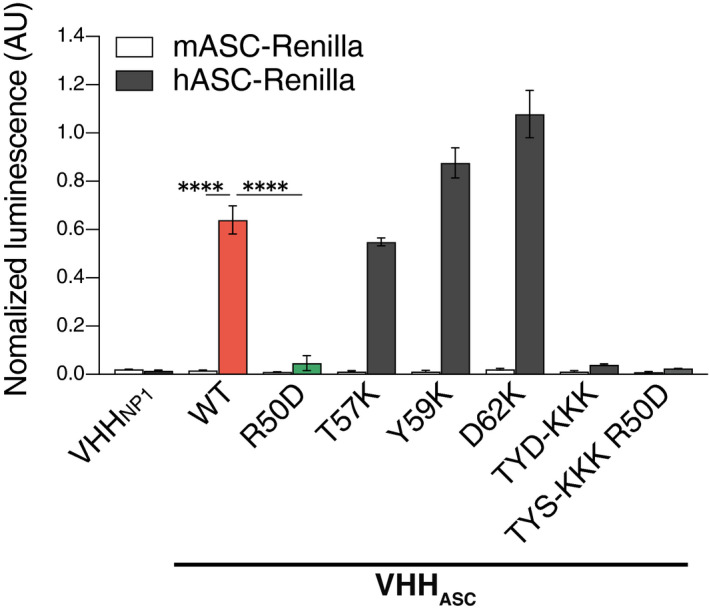
Binding activity and species specificity of ASC VHHs Lysates of HEK 293T cells transiently expressing HA‐tagged VHH_ASC_ (wild‐type or point mutants), an unrelated control (VHH_NP1_) and the indicated bait proteins fused to Renilla luciferase were used to immunoprecipitate VHHs with immobilized anti‐HA antibody. Renilla luciferase activity of the co‐immunoprecipitated proteins was measured and normalized to the input luciferase. Data represents mean values ± SEM from three independent experiments. ****P < 0.0001, One‐way ANOVA, multiple comparison (Tukey test). 'TYD‐KKK' indicates the triple mutant T57K, Y59K, and D62K.

We first tested the effect of nanobody directed against human ASC (VHH_ASC_) on the pro‐inflammatory function of ASC specks assembled *in vitro* from recombinant sources (Fernandes‐Alnemri *et al*, 2007; Fernandes‐Alnemri and Alnemri, 2008; Franklin *et al*, 2014). For this, recombinant human ASC specks were incubated with VHH_ASC_ prior to their addition to murine bone marrow‐derived macrophages (Fig 1A). To create a specific VHH control, we introduced a single structure‐guided amino acid mutation R50D into the complementarity determining regions (CDR2) of the VHH_ASC_, generating a variant (mutVHH_ASC_) unable to bind ASC, as shown by LUMIER assay (Fig EV1). We also tested several substitutions of single amino acids (T57, Y59, D62) present at the ASC/VHH_ASC_ interface (Schmidt *et al*, 2016) or their combinations (Fig EV1). The single R50D mutation showed the strongest loss of binding to ASC and was therefore used throughout this study (mutVHH_ASC_). As expected (Baroja‐Mazo *et al*, 2014; Franklin *et al*, 2014; de Almeida *et al*, 2015), macrophages incubated with ASC specks released IL‐1β into the culture supernatants. Notably, the pre‐incubation of ASC specks with VHH_ASC_, but not with mutVHH_ASC_ or a conventional polyclonal anti‐ASC Ab (anti‐ASC pAb), blunted the ability of ASC specks to trigger IL‐1β secretion from macrophages (Fig 1A). Treatment of macrophages with VHH_ASC_ alone did not induce IL‐1β release (Fig 1A). These results indicate that VHH_ASC_ interferes with the capacity of extracellular ASC specks to stimulate further intracellular processing of IL‐1β after their phagocytosis by surrounding macrophages.

Because extracellular ASC specks can also induce the maturation of caspase‐1 and IL‐1β in the extracellular space (Baroja‐Mazo *et al*, 2014; Franklin *et al*, 2014), we assessed the proteolytic processing of pro‐caspase‐1 and pro‐IL‐1β in the lysates of LPS‐primed ASC‐deficient (*Pycard* ^−/−^) immortalized murine macrophages incubated with ASC specks in the presence of increasing concentrations of VHH_ASC_ or mutVHH_ASC_. As previously reported (Baroja‐Mazo *et al*, 2014; Franklin *et al*, 2014; de Almeida *et al*, 2015), exogenous ASC specks induced the activation of pro‐caspase‐1 (p45), and pro‐IL‐1β (p31) contained in *Pycard* ^−/−^ lysates into their mature forms (p20 and p17, respectively, Fig 1B and C). VHH_ASC_ dose‐dependently inhibited the ASC specks‐induced activation of pro‐caspase‐1 and pro‐IL‐1β, while mutVHH_ASC_ had no effect. Consistent with our previous observations (Franklin *et al*, 2014), ASC specks contained little caspase‐1, but no detectable IL‐1β (Fig 1B), supporting that the detected proteins originate from the cleavage of their pro‐forms in the lysates. Together these data demonstrate that VHH_ASC_ blocks the pro‐inflammatory activity of extracellular ASC specks.

### VHH_ASC_ disassemble ASC oligomers and block the prionoid activities of ASC specks

**Figure 2:**
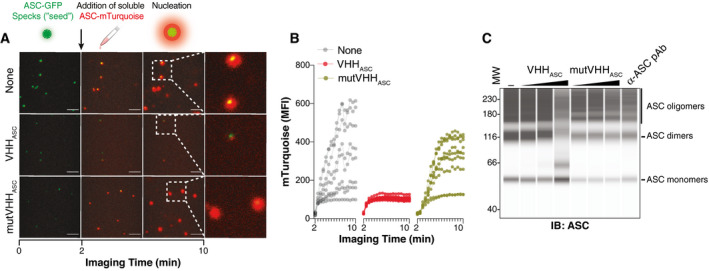
VHH_ASC_ disrupts the “prionoid” activities and disassembles ASC specks Time‐lapse confocal imaging of the *in vitro* nucleation of soluble ASC‐mTurquoise (red) by ASC‐TagGFP (ASC‐GFP) specks (green), that were left untreated (None), or pre‐incubated with VHH_ASC_ or mutVHH_ASC_ (200 µg ml^−1^ for 15 min). Scale bars: 10 μm.Median fluorescence intensity (MFI) of mTurquoise graphed over time showing its polymerization seeded by ASC‐TagGFP specks. Each line shows the mTurquoise MFI around a seeding ASC‐TagGFP speck. Data is one representative of three independent experiments.WES capillary electrophoresis and immunoblotting of DSS cross‐linked *in‐vitro*‐generated human ASC‐mTurquoise (ASC) specks that were incubated for 1 h (RT) with 2, 20 or 200 µg ml^−1^ of VHH_ASC_, mutVHH_ASC_ or with 20 µg ml^−1^ of a polyclonal anti‐ASC Ab (a‐ASC pAb). Data is from one representative of four independent experiments. Source data are available online for this figure.

In addition to its central function in assembling inflammasomes, ASC specks have prion‐like activities (Cai *et al*, 2014; Franklin *et al*, 2014; Lu *et al*, 2014) which propagate inflammation by nucleating the oligomerization of soluble ASC *in vitro*, and in the cytosol of macrophages that phagocytose ASC specks. We therefore assessed the effect of VHH_ASC_ or mutVHH_ASC_ on the nucleation of soluble ASC‐mTurquoise by ASC‐GFP specks. We imaged the recruitment of soluble ASC‐mTurquoise to ASC‐GFP specks that had been pre‐incubated with VHHs (Fig 2A and B). In line with our previous observations (Franklin *et al*, 2014), ASC‐GFP specks rapidly seeded the polymerization of soluble ASC‐mTurquoise. However, pre‐incubation with VHH_ASC_ prevented ASC‐GFP specks from seeding the nucleation of soluble ASC‐mTurquoise, while mutVHH_ASC_ had no effect (Fig 2A and B). To test whether VHH_ASC_ affected the integrity of specks, we assessed the stability of ASC oligomers using DSS cross‐link and immunoblotting (Fernandes‐Alnemri *et al*, 2007; Fernandes‐Alnemri and Alnemri, 2008; Franklin *et al*, 2014; Hoss *et al*, 2018; Fig 2C). VHH_ASC_ dose‐dependently disrupted the oligomeric conformation of ASC specks, demonstrated by the decreased proportion of ASC oligomers to monomers (Fig 2C), confirming previous findings that VHH_ASC_ shifts GFP‐ASC^CARD^ from an oligomeric to a monomeric conformation (Schmidt *et al*, 2016b). In contrast, mutVHH_ASC_ had no effect on the conformation of ASC specks, nor did the incubation with an ASC‐specific pAb (Fig 2C). Hence, VHH_ASC_ masks and prevents critical ASC^CARD^‐ASC^CARD^ interactions within specks disassembling ASC oligomers, and blocking their prion‐like functions.

### VHH_ASC_ requires access to cytosolic ASC to prevent inflammasome activation in intact cells

**Figure 3:**
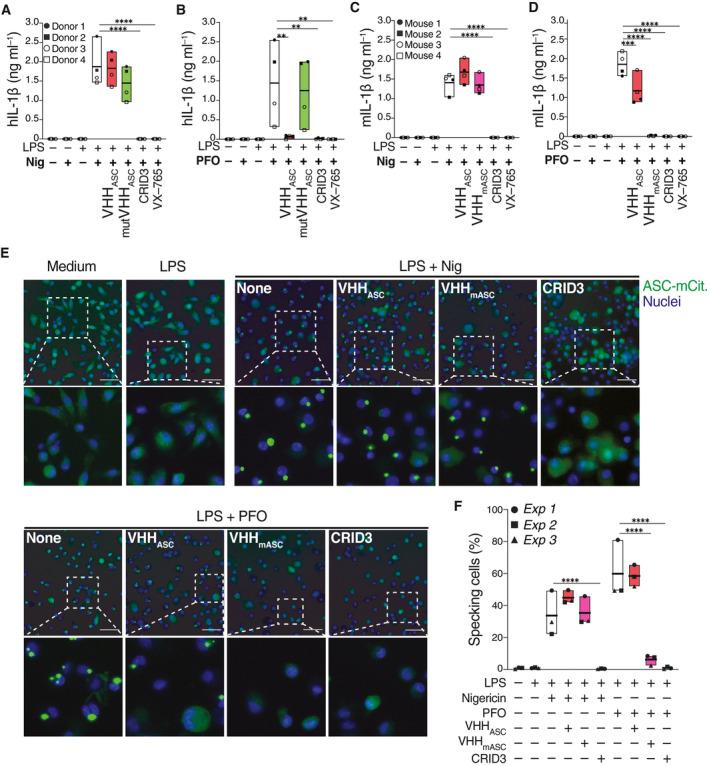
VHH_ASC_ and VHH_mASC_ prevent IL‐1β release and ASC speck formation induced by PFO, but not nigericin in primary human and mouse cells A–D(A, B) Human IL‐1β (hIL‐1β) concentrations in cell‐free supernatants of LPS‐primed (10 ng ml^−1^, 150 min) primary human macrophages that were left untreated, or pre‐incubated with VHH_ASC_ or mutVHH_ASC_ (100 µg ml^−1^), CRID3 (50 µM) or VX‐765 (50 µM) for 30 min before stimulation with (A) nigericin (10 µM), or (B) PFO (30 ng ml^−1^) for 2 h. (C‐D) Mouse IL‐1β (mIL‐1β) concentrations in cell‐free supernatants of LPS‐primed mouse BMDMs (200 ng ml^−1^, 150 min), incubated with VHHs, CRID3 or VX765, before activation with nigericin (10 µM), or PFO (250 ng ml^−1^). Data is combined from two independent experiments, each performed with two donors (A, B) or mice (C, D), represented with individual symbols (4 donors or mice in total). Data is displayed as floating bars with the max/min values and mean (thicker band).E, F(E) Epifluorescence microscopy imaging and (F) quantification of ASC speck formation in BMDMs from ASC‐mCitrine (Green) transgenic mice. Cells were primed with LPS (200 ng ml^−1^, 150 min), pre‐treated with VX‐765 (50 µM, 30 min), then treated with VHH_ASC_, VHH_mASC_ (100 µg ml^−1^) or CRID3 (50 µM) for another 30 min before stimulation with nigericin (top), or PFO (bottom) for 2 h and finally fixed with 4% PFA. Nuclei was stained with DRAQ5 (Blue). Scale bars: 100 μm. Images in (E) are from one representative out of three independent experiments that were quantified in F. Data in F is displayed as floating bars with the max/min values and mean (thicker band). Data information: **P < 0.005; ***P < 0.0002; ****P < 0.0001, One‐way ANOVA, multiple comparison (Tukey test).

**Figure EV2:**
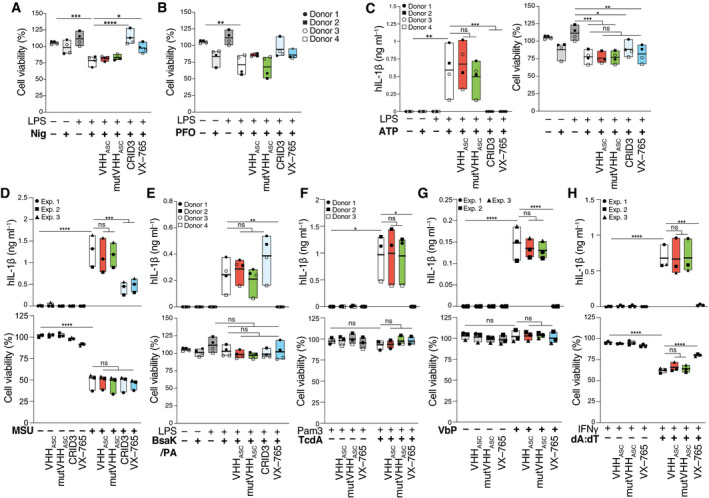
VHH_ASC_ preserves endogenous inflammasome activation in human living cells A, BCell viability (CTB) assay on LPS‐primed primary human macrophages that were left untreated, or pre‐incubated with VHH_ASC_ or mutVHH_ASC_ (100 µg ml^−1^), CRID3 (50 µM) or VX‐765 (50 µM) for 30 min before being activated with (A) nigericin (10 µM), or (B) PFO (30 ng ml^−1^) for 2 h. Data is from the experiments displayed in Fig 3A and B.CHuman IL‐1β (hIL‐1β) concentrations in cell‐free supernatants (left), and cell viability assay (right) of LPS‐primed primary human macrophages that were incubated with VHH_ASC_ or mutVHH_ASC_ (100 µg ml^−1^), CRID3 (50 µM) or VX‐765 (50 µM) for 30 min before being stimulated with 2.5 mM ATP.DhIL‐1β concentrations in cell‐free supernatants (top), and cell viability assay (bottom) of PMA‐differentiated THP‐1 cells treated with VHH_ASC_ or mutVHH_ASC_ (100 µg ml^−1^), CRID3 (10 µM) or VX‐765 (50 µM) for 30 min before 4.5 h stimulation with 250 µg ml^−1^ MSU crystals.EhIL‐1β concentrations in cell‐free supernatants (top), and cell viability assay (bottom) of LPS‐primed primary human macrophages that were incubated with VHH_ASC_ or mutVHH_ASC_ (100 µg ml^−1^), CRID3 (50 µM) or VX‐765 (50 µM) for 30 min before being stimulated with 0.1 µg ml^−1^/0.5 µg ml^−1^ mixture of LFn‐BsaK and PA for 2 h.FhIL‐1β concentrations in cell‐free supernatants (top), and cell viability assay (bottom) of Pam3CysK4‐primed (1 µg ml^−1^) primary human CD14^+^ monocytes that were incubated with VHH_ASC_ or mutVHH_ASC_ (100 µg ml^−1^), or VX‐765 (50 µM) for 30 min before being stimulated with 1 µg ml^−1^ TcdA.GhIL‐1β concentrations in cell‐free supernatants (top), and cell viability assay (bottom) of keratinocyte cells (N‐TERT) that were treated with VHH_ASC_ or mutVHH_ASC_ (100 µg ml^−1^), or VX‐765 (50 µM), then directly stimulated with 30 µM Val‐boroPro (VbP) for 22 h.HhIL‐1β concentrations in cell‐free supernatants (top), and cell viability assay (bottom) of PMA‐differentiated THP‐1 cells treated with IFNγ (500 U ml^−1^) for 16 h and that were incubated with VHH_ASC_ or mutVHH_ASC_ (100 µg ml^−1^), CRID3 (10 µM) or VX‐765 (50 µM) for 30 min before 2 h stimulation with 1 µg ml^−1^ poly(dA:dT) in complex with Lipofectamine 2000. Data information: Data is representative of either two independent experiments, each run with one to two donors (A–C, E, F, 3 or 4 donors in total) or at least 3 independent experiments (D, G, H). Each symbol represents one donor or independent experiment. ^ns^ *P* > 0.05; *P < 0.05; **P < 0.005; ***P < 0.0002; ****P < 0.0001, One‐way ANOVA, multiple comparison (Tukey test). Data is displayed as floating bars with the max/min values and mean (thicker band).

Next, we investigated whether the addition of VHH_ASC_ directly into culture supernatants of inflammasome‐activated cells affects the intracellular processing of caspase‐1 and IL‐1β. We pre‐incubated LPS‐primed primary human macrophages (hMDMs) with VHH_ASC_ or mutVHH_ASC_. As controls, we pre‐treated hMDMs with CRID3 (MCC950) (Coll *et al*, 2015) or VX‐765, two well‐characterized inhibitors of the NRLP3 and caspase‐1, respectively. Cells were then treated with nigericin, for the activation of NLRP3 (Figs 3A and EV2A). As expected, both CRID3 and VX‐765 abrogated the IL‐1β release (Fig 3A) and prevented cell death (Fig EV2A) induced by stimulation with nigericin. Notably, neither VHH_ASC_ nor mutVHH_ASC_ had any effect on IL‐1β secretion or the viability of NLRP3‐activated cells (Figs 3A and EV2A). Similar results were observed in cells stimulated with ATP (NLRP3 in, Fig EV2C), MSU (NLRP3 in PMA‐treated THP‐1, Fig EV2D), a combination of LFn‐BsaK and PA (NLRC4 in hMDMs, Fig EV2E), TcdA (Pyrin in CD14^+^ monocytes, Fig EV2F), Val‐boroPro (NLRP1 in N/TERT‐1 keratinocytes, Fig EV2G), or poly(dA:dT) (AIM2 in PMA‐ and IFNγ‐treated THP‐1, Fig EV2H), indicating that our finding can be generalized to other NLRP3 activators, and other inflammasomes. Based on these findings, we speculated that VHH_ASC_ was unable to cross the intact plasma membrane early enough to prevent inflammasome activation in intact cells. To address this hypothesis, we stimulated LPS‐primed hMDMs with the pore‐forming toxin perfringolysin O (PFO), which forms membrane pores of ~40 nm reminiscent of GSDMD pores (Czajkowsky *et al*, 2004; Mulvihill *et al*, 2018), and was previously shown to induce the potassium efflux‐dependent activation of NLRP3 (Yamamura *et al*, 2019). Supporting our hypothesis, the presence of VHH_ASC_ in the supernatants completely abrogated the release of IL‐1β from PFO‐treated macrophages, similarly to CRID3 and VX‐765 (Fig 3B). The mutant nanobody, mutVHH_ASC_, failed to block IL‐1β release. While IL‐1β release was efficiently blunted in VHH_ASC_‐treated cells, VHH_ASC_ could not restore cell viability induced by PFO (Fig EV2B), likely due to the loss of membrane integrity caused by PFO pores. Supporting this view, neither CRID3 nor VX‐765 could rescue cell viability in PFO‐activated cells (Fig EV2B).

**Figure EV3:**
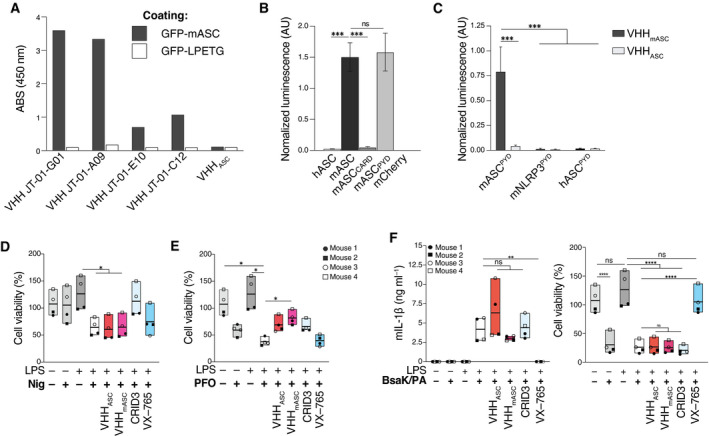
VHH_mASC_ is specific to ASC^PYD^ but retains the same activity as VHH_ASC_ ASpecificity of different single‐domain antibodies (VHHs) probed by ELISA. Recombinant murine ASC as an eGFP fusion or eGFP alone (GFP‐LPETG) as control, were coated onto ELISA plates at 1 µg ml^−1^/well. Wells were incubated with HA‐tagged VHHs (100 nM), anti‐HA‐tag mouse mAb coupled to HRP, and the HRP substrate TMB. Binding was quantified by measuring the absorbance at 450 nm.B, CLysates of HEK 293T cells transiently expressing HA‐tagged VHH_mASC_ or VHH_ASC_ and the indicated bait proteins fused to Renilla luciferase were used to immunoprecipitate VHHs with immobilized anti‐HA antibody. Renilla luciferase activity of the co‐immunoprecipitated proteins was measured and normalized to the input luciferase. Data represents mean values ± SEM from three independent experiments.D, ECell viability (CTB) assay on LPS‐primed (200 ng ml^−1^) primary mouse BMDMs that were left untreated, or pre‐incubated with VHH_ASC_ or VHH_mASC_ (100 µg ml^−1^), CRID3 (50 µM) or VX‐765 (50 µM) for 30 min before being activated with (D) nigericin (10 µM), or (E) PFO (250 ng ml^−1^) for 2 h. Data is from the experiments displayed in Fig 3C and D. Data is displayed as floating bars with the max/min values and mean (thicker band).FMouse IL‐1β (mIL‐1β) concentrations in cell‐free supernatants (left), and cell viability assay (right) of LPS‐primed mouse BMDMs incubated with VHHs, CRID3 or VX–765, before stimulation with 0.1 µg ml^−1^/0.5 µg ml^−1^ mixture of LFn‐BsaK and PA for 2 h. Each symbol represents one mouse. Data is displayed as floating bars with the max/min values and mean (thicker band). Data information: ^ns^ *P* > 0.05; ***P < 0.001; ****P < 0.0001, Two‐way (A, C) or One‐way (B, D–F) ANOVA, multiple comparison (Tukey test).

As shown by the stringent LUMIER assay (Fig EV1), VHH_ASC_ does not efficiently bind murine ASC. We therefore generated mouse‐specific anti‐ASC VHHs (VHH_mASC_) by immunizing an alpaca with murine ASC, and screening for nanobodies able to target mouse ASC. We identified several VHHs specific for murine ASC by phage display and confirmed binding by ELISA assay with recombinant proteins (Fig EV3A). VHH JT‐01‐A09 was selected for further use in this manuscript (VHH_mASC_). We further characterized binding of VHH_mASC_ to mouse ASC with LUMIER assays, which evaluate the cytosolic interaction of HA‐tagged VHH_mASC_ with bait proteins fused to Renilla luciferase. These experiments revealed that VHH_mASC_ functions in the reducing environment of the cytosol, and that VHH_mASC_ binds to the pyrin domain (ASC^PYD^) of mouse ASC, but neither the CARD (ASC^CARD^), nor the human ASC (Fig EV3B). To demonstrate the specificity of VHH_mASC_ to mouse ASC^PYD^, we also tested for binding of VHH_mASC_ to mouse NLRP3^PYD^ or human ASC^PYD^ (Fig EV3C). As expected, VHH_mASC_ exclusively bound mouse ASC^PYD^, but no other PYDs (Fig EV3C). We then tested whether VHH_mASC_ is also capable of inhibiting the formation of ASC specks in mouse bone marrow‐derived macrophages (BMDMs). Similar to the human‐specific VHH_ASC_, VHH_mASC_ had no effect on IL‐1β release or survival of LPS‐primed BMDMs stimulated with nigericin (Figs 3C and EV3D) or BsaK (Fig EV3F). In line with the activity of VHH_ASC_ in human macrophages (Fig 3B), pre‐treatment of murine BMDMs with VHH_mASC_ fully abrogated the PFO‐induced release of IL‐1β to a similar degree to CRID3 or VX‐765 (Fig 3D), and partially rescued cell viability (Fig EV3B). Interestingly, when tested in mouse BMDMs, the anti‐human ASC nanobody (VHH_ASC_) partially impaired the PFO‐induced release of IL‐1β, and rescued cell viability (Figs 3D and EV3B), indicating some limited cross‐reactivity.

Next, we tested the activity of VHH_ASC_ and VHH_mASC_ on BMDMs derived from transgenic mice expressing mouse ASC in fusion with mCitrine (Tzeng *et al*, 2016). This allowed us to study the activity of VHH_ASC_ or VHH_mASC_ on the intracellular formation of ASC‐mCitrine specks (Fig 3E and F). To prevent the formation of GSDMD pores and restrict nanobody access to the cytosol, we treated cells with VX‐765. As expected, primed macrophages treated with nigericin or PFO displayed the typical fluorescent ASC puncta (Fig 3E). Pre‐treatment with CRID3 entirely abrogated the formation of specks in either nigericin‐ or PFO‐treated cells. In cells stimulated with nigericin, pre‐treatment with VHH_ASC_ or VHH_mASC_ had no effect (Fig 3E and F). In contrast, in PFO‐stimulated cells, VHH_mASC_ strongly prevented ASC specks formation, while VHH_ASC_ had no effect, confirming the limited cross‐reactivity between VHH_ASC_ and mouse ASC.

**Figure 4:**
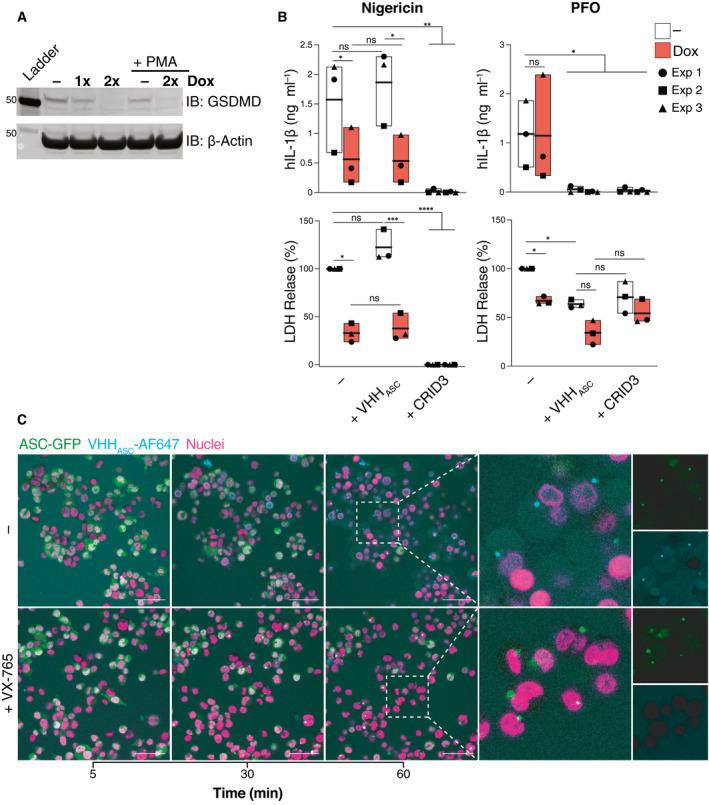
Contribution of GSDMD in the effect of VHH_ASC_ on the release of IL‐1β and cell death induced by PFO or nigericin THP‐1 cells expressing a Dox‐inducible CRISPR‐Cas9 cassette targeting GSDMD were left untreated (–), or treated with 1 µg ml^−1^ Dox for one or two cycles of 72 h (1×, or 2× respectively).
Immunoblot analysis of GSDMD expression following the indicated course of Dox treatment and PMA‐differentiation, as indicated. Data is from one representative of two independent experiments.IL‐1β concentration or percentage of LDH released into cell‐free supernatants of PMA‐differentiated THP‐1 cells that were treated with VHH_ASC_ (200 µg ml^−1^) or CRID3 (25 µM) for 30 min prior to stimulation with nigericin (10 µM, left panels) or PFO (30 ng ml^−1^, right panels) for 2 h. Data is average of experimental duplicates from three independent experiments, each represented by a different symbol. ^ns^ *P* > 0.05; *P < 0.05; **P < 0.01; ***P < 0.001; ****P < 0.0001, Two‐way ANOVA, multiple comparison (Tukey test). Data is displayed as floating bars with the max/min values and mean (thicker band).Live confocal imaging of PMA‐differentiated and nigericin‐treated (10 µM) THP‐1 cells expressing human ASC‐GFP (green) in the presence of AlexaFluor647‐labeled VHH_ASC_ (VHH_ASC_‐AF647, 10 µg ml^−1^, cyan) in the medium. Cells were either left untreated (–) or incubated with VX‐765 (50 µM) for 1 h prior to nigericin stimulation. Nuclei were stained with Hoechst 34580 (magenta). Scale bar: 50 µm. Data is from one representative out of two independent experiments. Source data are available online for this figure.

**Figure EV4:**
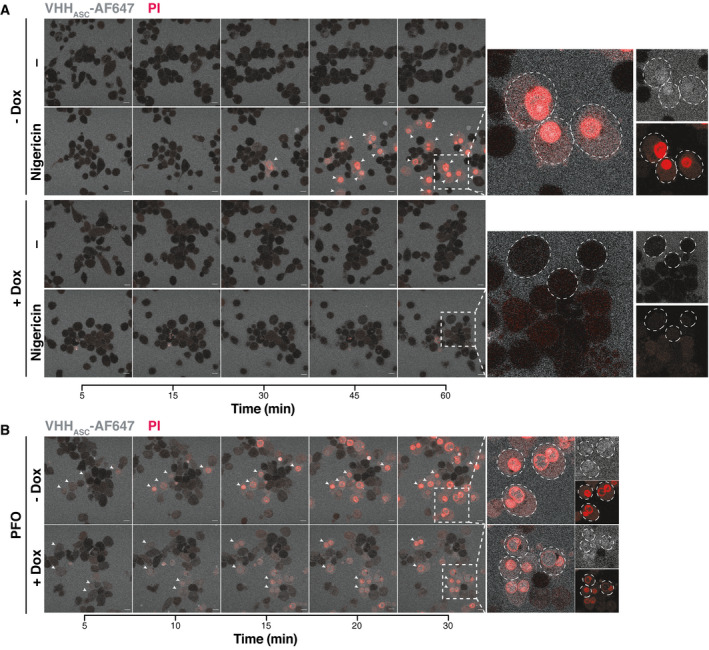
Contribution of GSDMD in the effect of VHHASC on the internalization of VHH_ASC_ in cells stimulated with PFO or nigericin THP‐1 cells expressing a Dox‐inducible CRISPR‐Cas9 cassette targeting GSDMD gene were left untreated (–) or treated with 1 µg ml^−1^ Dox for one or two cycles of 72 h (1×, or 2× respectively).
A, BConfocal microscopy images of GSDMD competent (–Dox) or GSDMD‐KO (+Dox) cells primed with PMA and that were either left untreated or treated with (A) nigericin (10 µM) or (B) PFO (30 ng ml^−1^) for the indicated periods of time and in the presence of AlexaFluor647‐labeled VHH_ASC_ (VHH_ASC_‐AF647, 10 µg ml^−1^, blue) and propidium iodide (PI, 3.33 µg ml^−1^, red). White arrows indicate of cells with intracellular VHH_ASC_‐AF647 signal. Dashed circles define boundaries of representative cells. Data is from one representative out of three independent experiments. Scale bar: 10 µm. Also see Movie EV1.

Together, our data suggest that membrane pores are required for VHHs to access the cytosol of inflammasome‐activated cells. To consolidate these findings, we tested the contribution of GSDMD in the effect of VHH_ASC_. For this, we used THP‐1 cells expressing a doxycycline (Dox)‐inducible CRISPR‐Cas9 cassette as well as a sgRNA targeting GSDMD (Budden *et al*, 2021; Fig 4). Doxycycline treatment initiated GSDMD knockout and efficiently abrogated GSDMD expression (Fig 4A). As expected, GSDMD deficiency strongly attenuated the release of IL‐1β and LDH from nigericin‐activated cells (Fig 4B, left panels), but had minor impact on the release of IL‐1β from PFO‐treated cells (Fig 4B, upper right panel). Irrespective of GSDMD expression, VHH_ASC_ blunted the release of IL‐1β induced by PFO (Fig 4B, upper right panel), while it had no effect on nigericin‐activated cells (Fig 4B, upper left panel). As expected, CRID3 treatment completely blocked IL‐1β and LDH release in response to both nigericin and PFO (Fig 4B, upper panels). PFO‐treated GSDMD‐KO cells released lower amounts of LDH compared with GSDMD competent cells, indicating that LDH release induced by PFO partly depends on GSDMD (Fig 4B, lower right panel). The presence of VHH_ASC_ further decreased the release of LDH in both GSDMD competent and knockout cells (Fig 4B, lower right panel). To study the kinetic of VHH_ASC_ uptake by inflammasome‐activated cells, we performed live imaging on PMA‐primed and nigericin‐stimulated ASC‐GFP expressing THP‐1 cells in the presence of low dose (10 µg ml^−1^) of fluorescently‐labeled VHH_ASC_ (VHH_ASC_‐AF647) directly applied into the culture supernatants (Fig 4C). Inflammasome activation allowed the entry of VHH_ASC_‐AF647 which co‐localized to endogenous ASC‐GFP specks. VHH_ASC_‐AF647 staining of GFP specks was prevented by the caspase‐1 inhibition with VX‐765 (Fig 4C). Confirming the requirement for GSDMD for VHH_ASC_ entry into cells, deletion of GSDMD (+Dox) prevented both cell death (propidium iodide staining) and VHH_ASC_‐AF647 internalization (Fig EV4A and Movie EV1). Notably, upon stimulation with PFO, VHH_ASC_‐AF647 rapidly entered the cells (~ 5 min) in a manner that was independent of GSDMD expression (Fig EV4B and Movie EV1). Together, these findings confirm that early access to the cell cytosol correlates with the ability of VHH_ASC_ to interfere with ASC specks formed from inflammasome activation inside cells. Hence, in our previous experiments, PFO pores and not GSDMD allowed VHH_ASC_ access to the cytosol to target endogenous ASC and prevent inflammasome activation and IL‐1β release. These experiments also demonstrate that upon stimulation with conventional inflammasome activators (e.g., nigericin), VHH_ASC_ predominantly targets ASC specks in the extracellular space, or exposed in pyroptotic cells, and that GSDMD‐dependent membrane permeabilization is required for targeting intracellular ASC. We could also demonstrate the species specificity of VHH_ASC_, and discovered a new mouse‐specific nanobody targeting ASC^PYD^ with inhibitory properties similar to VHH_ASC_.

### VHH_ASC_ targets ASC specks in inflammasome‐activated cells undergoing pyroptosis

**Figure 5:**
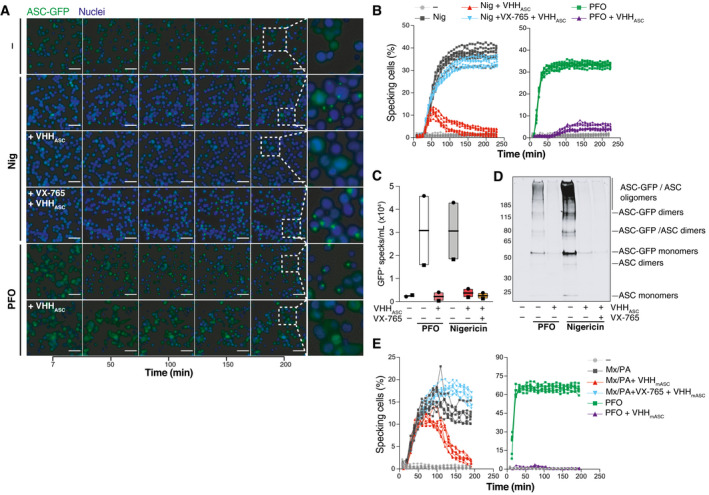
Pyroptosis allows VHH_ASC_ to access the cytosol and target ASC specks AWide‐field fluorescence microscopy imaging of live PMA‐differentiated and nigericin‐ (10 µM, top) or PFO‐activated (25 ng ml^−1^, bottom) THP‐1 expressing human ASC‐GFP. Cells were incubated with VHH_ASC_ (200 µg ml^−1^) alone or in the presence of the caspase‐1 inhibitor VX‐765 (50 µM). Nuclei were stained with DRAQ5 (blue). Cells were imaged live with a CellDiscoverer 7 microscope. For each condition, a total of 8 positions within 2 wells (2 × 4 images/well) were imaged. Data is maximal intensity projections from Z‐stacks. Scale bars: 50 µm. See also Movie EV2.BGraphic representation of the percent of specking cells over time. Maximal intensity projections from Z‐stacks were generated for each image set before the number of cells and specks per field were calculated using CellProfiler. (A) represents images from one experiment out of three independent experiments of which percentage of specking cells data is represented in (B) with each line representing one field‐of‐view.C, D(C) Flow cytometry quantification, and (D) immunoblotting analysis of ASC‐GFP oligomers (DSS cross‐linked) in extracellular specks recovered from supernatants of cells treated as in A. Data in (C) is from two independent experiments (each represented by a different symbol). And in (D) is from one out of at least two independent experiments. Data is displayed as floating bars with the max/min values and mean (thicker band).EMouse BMDMs expressing ASC‐mCitrin and primed with LPS (200 ng ml^−1^) for 3 h were pre‐treated with VHH_mASC_ (200 µg ml^−1^) alone or in the presence of the caspase‐1 inhibitor VX‐765 (50 µM) for 30 min. Cells were then treated with a mixture of LFn‐MxiH and PA (Mx/PA, 100 ng ml^−1^/1 µg ml^−1^) or PFO (250 ng ml^−1^) and imaged as in (A) for 3 h. Data is a graphic representation of specking cells over time calculated as in (B). Data is from one experiment out of three independent experiments. See also Movie EV6. Source data are available online for this figure.

**Figure EV5:**
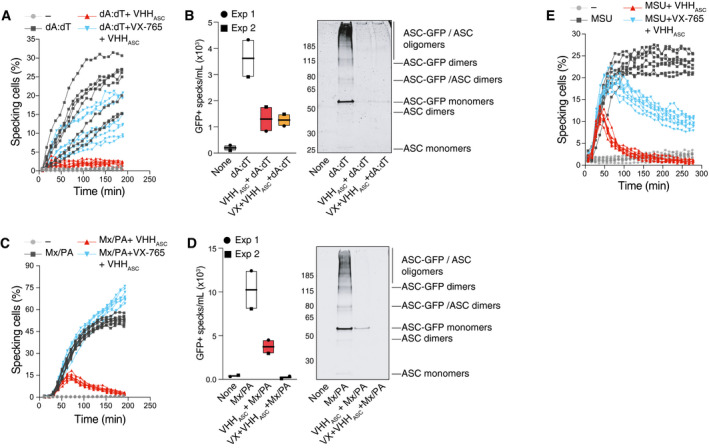
VHH_ASC_ disrupts post‐pyroptotic ASC specks PMA‐differentiated (50 ng ml^−1^) THP‐1 ASC‐GFP cells were treated with IFNγ (500 U ml^−1^) for 16 h. Then, cells were pre‐incubated with VHH_ASC_ (100 µg ml^−1^), or VX‐765 (50 µM) for 30 min before being activated with poly(dA:dT) (dA:dT, 1 µg ml^−1^) and imaged live with a CellDiscoverer 7 microscope as described in Fig 4 over 3 h. Data is a graphic representation of the percent of specking cells over time. Maximal intensity projections from Z‐stacks were generated for each image set (8 images per condition) before the number of cells and specks per field were calculated using CellProfiler. Also see Movie EV3.Flow cytometric quantification (left), or western‐blot (right, following DSS cross‐linking) assessement of GFP^+^ specks in the supernatants of cells pre‐treated with VHH_ASC_ or VX‐765 and then activated with poly(dA:dT) for 3.5 h. Data in left panel is displayed as floating bars with the max/min values and mean (thicker band).Flow cytometric quantification or western‐blotting of GFP+ specks released by PMA‐differentiated THP‐1 ASC‐GFP cells pre‐incubated with VHH_ASC_ (100 µg ml^−1^), or VX‐765 (50 µM) for 30 min before stimulation with LFn‐MxiH/PA (100 ng ml^−1^/1 µg ml^−1^) for 3 h and imaged live with a CellDiscoverer 7 microscope. Also see Movie EV4.GFP^+^ specks detected by flow cytometry (left) or western‐blot (right, following DSS cross‐linking) in the supernatants of cells pre‐treated with VHH_ASC_ or VX‐765 and then activated with LFn‐MxiH/PA for 3.5 h. Data in left panel is displayed as floating bars with the max/min values and mean (thicker band).PMA‐differentiated THP‐1 ASC‐GFP cells were pre‐incubated with VHH_ASC_ (100 µg ml^−1^), or VX‐765 (50 µM) for 30 min before stimulation with MSU crystals (250 µg ml^−1^) for 4.5 h and imaged live with a CellDiscoverer 7 microscope. Imaging and quantifications were done as in (A). Also see Movie EV5. Source data are available online for this figure.

Having shown that ectopic treatment with VHH_ASC_ does not prevent the initial assembly of ASC specks in cells treated with nigericin, we wondered whether VHH_ASC_ could engage with their intracellular targets after the onset of pyroptosis. We therefore performed live cell imaging on PMA‐differentiated THP‐1 macrophages expressing fluorescent ASC‐GFP and followed the dynamics of ASC speck formation upon treatment with nigericin or PFO (Fig 5A and B and Movie EV2). As expected, nigericin and PFO induced a rapid increase in the proportion of cells assembling ASC specks that plateaued after 100 or 40 min, respectively (Fig 5A and B and Movie EV2). The presence of VHH_ASC_ in PFO‐treated cells almost entirely abrogated the formation of ASC specks, confirming that the rapid access of VHH_ASC_ to the cytosol (Fig EV4B) is allowed by PFO pores (Fig 5B, right panel and Movie EV2). In contrast, the proportion of nigericin‐treated cells containing ASC‐GFP specks in the presence of VHH_ASC_ initially increased, peaking at 50 min (Fig 5B, left panel and Movie EV2), but decreased to levels close to untreated cells at later time points (Fig 5B, left panel and Movie EV2). These findings indicate that the GSDMD‐dependent membrane perforation granting VHH_ASC_ access to the cytosol (Figs 4C and EV4A) enabled interference with ASC specks inside pyroptotic cells. Further supporting this conclusion and previous data (Fig 4C), caspase‐1 inhibition with VX‐765 prevented VHH_ASC_ from targeting and disassembling cytosolic ASC‐GFP specks in nigericin‐treated cells (Fig 5B, left panel and Movie EV2). Hence, GSDMD‐dependent plasma membrane perforation allows VHH_ASC_ to target exposed ASC specks in pyroptotic cells without affecting pre‐pyroptotic IL‐1β maturation (Zhou & Abbott, 2021). To exclude that the decreased number of specks detected in the presence of VHH_ASC_ was not due to the release of specks into the extracellular space (Baroja‐Mazo *et al*, 2014; Franklin *et al*, 2014), we quantified ASC specks in the culture supernatants (Fig 5A and B) by flow cytometry, or the formation of ASC oligomers by DSS‐cross‐linking and immunoblotting (Fig 5C and D and Appendix Fig S1). Consistent with the ability of VHH_ASC_ to disassemble ASC oligomers, supernatants of cells treated with nigericin or PFO in the presence of VHH_ASC_ had decreased amounts of GFP‐positive specks (Fig 5C and D and Appendix Fig S1B). These findings were reproducible in cells stimulated with poly(dA:dT) (Fig EV5A and B and Movie EV3), MxiH (Fig EV5C and D and Movie EV4), or MSU (Fig EV5E and Movie EV5) confirming the broad effects of the VHH_ASC_ against ASC specks assembled by AIM2, NLRC4, and different NLRP3 triggers. In cells treated with nigericin (NLRP3), poly(dA:dT) (AIM2), and MxiH (NLRC4), the inhibitory effect of VHH_ASC_ was lost when caspase‐1 (and thus GSDMD pore formation) was inhibited with VX‐765. A notable exception were MSU‐treated cells, in which prolonged incubation with VHH_ASC_ decreased the number of ASC‐GFP speck even in the presence of VX‐765, (Fig EV5E and Movie EV5). This suggested that prolonged exposure to MSU crystals enabled the entry of VHH_ASC_ into cells, likely due to crystal‐mediated disruption of cellular membranes (Hornung *et al*, 2008; Duewell *et al*, 2010).

As VHH_ASC_ can access ASC specks in the cytosol of nigericin‐treated cells at later time points, we wondered whether delayed addition of VHH_ASC_ to culture supernatants could affect the IL‐1β output of cells stimulated with nigericin for a longer period. We treated LPS‐primed human primary macrophages with nigericin for 90 min, then added either VHH_ASC_ or CRID3 for another 7 h (Appendix Fig S2). Notably, neither VHH_ASC_ nor CRID3 had any effect on the output of IL‐1β from these cells. Indeed, cells treated for extended times released similar amounts of IL‐1β as in the first 90 min of nigericin‐activation (Appendix Fig S2), indicating that the maximal IL‐1β production is reached early during inflammasome activation in primary human macrophages. This would explain why pre‐treatment with VHH_ASC_ had no effect on IL‐1β release in nigericin‐treated macrophages (Fig 3), while it still disassembled post‐pyroptotic ASC speck (Fig 5).

Finally, we tested whether VHH_mASC_ can target post‐pyroptotic specks upon stimulation of NLRC4 (MxiH) or NLRP3 (PFO) in mouse macrophages (Fig 5E and Movie EV6). Similar to the activity of VHH_ASC_ in nigericin‐treated human macrophages (Fig 5B), the presence of VHH_mASC_ initially allowed for the initial formation of ASC‐mCitrine specks inside stimulated cells (Fig 5E, left panel). However, VHH_mASC_ induced a decrease in the proportion of cells containing ASC‐mCitrine specks at later time points. This effect was abolished by VX‐765, confirming the requirement for pyroptosis to allow VHH_mASC_ access to the cytosol (Fig 4E, left panel). In contrast, in cells stimulated with PFO, no assembly of ASC‐GFP specks was observed in the presence VHH_mASC_ (Fig 4E, right panel), as seen in human cells (Fig 4B, right panel). Taken together, these data demonstrate that both VHH_ASC_ and VHH_mASC_ can target and disassemble ASC specks in pyroptotic cells, while preserving initial IL‐1β secretion. They further demonstrate that VHH_ASC_ and VHH_mASC_ can target inflammasomes that recruit ASC either via its CARD or via its PYD, indicating that the activity of VHH_ASC_ and VHH_mASC_ resides at the interface between ASC molecules rather than between ASC and the inflammasome sensor.

### VHH_mASC_ ameliorates inflammation and clinical disease in gout

Next, we tested the inhibitory activity of a mouse‐specific VHH_mASC_ *in vivo* in the inflammasome‐dependent inflammatory model of gouty arthritis, induced by the intra‐articular injection of monosodium urate (MSU) crystals (Martinon *et al*, 2006). We first used a prophylactic approach where mice were given an intra‐peritoneal injection of VHH_mASC_ or PBS (as vehicle) 1 h prior to challenge with MSU crystals (Appendix Fig S3A). We measured the clinical and immunological parameters of gout, such as joint swelling, pain sensitivity, leukocyte infiltration, and tissue levels of pro‐inflammatory markers (Martinon *et al*, 2006). As expected, mice treated with MSU had a marked decrease in mechanical withdrawal threshold, indicating an increased sensitivity to painful stimuli when compared with vehicle‐treated or unchallenged VHH_mASC_‐treated animals (Appendix Fig S3B). Notably, the systemic administration of VHH_mASC_ significantly reduced the nociceptive behavior (Appendix Fig S3B) and joint swelling (Appendix Fig S3C) in MSU‐challenged animals. The MSU challenge also resulted in the infiltration of granulocytes (CD45^+^/Ly6G^+^) and inflammatory monocytes (CD45^+^/Ly6G^−^/Ly6C^+^) into the synovial space, which were markedly reduced in animals pre‐treated with VHH_mASC_ (Appendix Fig S3D and Appendix Fig S4D). In line with an anti‐inflammatory effect of VHH_mASC_, pre‐treatment with VHH_mASC_ strongly decreased the MSU‐induced tissue concentrations of IL‐1β and IL‐6 while it only partially decreased TNFα levels (ANOVA, *P* = 0.29) (Appendix Fig S3E).

Together with our observations that ASC‐specific VHHs target ASC following pyroptosis (Figs 3 and 4), these findings suggest that VHH_mASC_ administered systemically can reach the inflamed tissue to target ASC specks exposed to the extracellular space. VHHmASC may thus inhibit the amplification of the inflammation induced by ASC specks exposed to the extracellular space. To validate these findings, we also tested the efficacy of local injection of VHH_mASC_ together with MSU crystals into the joints. Recapitulating their systemic effect, local injection of VHH_mASC_ reversed the MSU‐induced nociception and edema (Appendix Fig S4A and B). Cell infiltration, especially of granulocytes, were reduced in animals treated with VHH_mASC_ together with MSU, when compared with those treated with MSU alone (Appendix Fig S4C).

**Figure 6:**
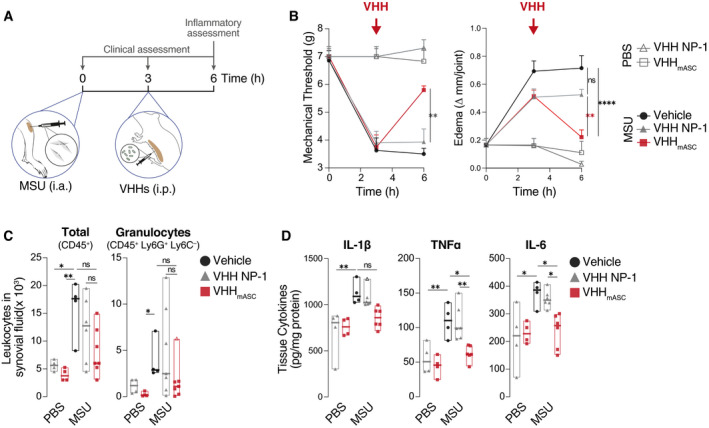
VHH_mASC_ ameliorates MSU gouty inflammation Schematic representation of the experimental setting used for the MSU‐gout model. Mice were injected i.a with 100 µg of monosodium urate (MSU) crystals into the knee. After 3 h, mice were treated intraperitonially (i.p.) with VHH_mASC_, VHH NP‐1 as unrelated nanobody (both 5 mg kg^−1^), or vehicle (PBS).Mechanical allodynia threshold and edema were evaluated at 3 and 6 h post‐MSU challenge. Error bars represent SEM from biological replicates: t0, *n* = 3 for all groups; t3 and t6: PBS + VHH NP‐1 and PBS + VHH_mASC_, *n* = 3 (Mechanical threshold) or *n* = 4 (Edema); MSU + Vehicle, *n* = 4; MSU + VHH NP‐1, *n* = 7; MSU + VHHmASC, *n* = 7.Flow cytometric assessment of infiltrating total leukocytes (CD45^+^) and granulocytes (CD45^+^ Ly6G^+^ Ly6C^−^) recovered in the synovial fluid of the knee joints of mice treated as in (A). Data in (C) is displayed as floating bars with the max/min values and mean (thicker band). Biological replicates are: PBS + VHH NP‐1, *n* = 4; PBS + VHH_mASC_, *n* = 4; MSU + Vehicle, *n* = 4; MSU + VHH NP‐1, *n* = 7; MSU + VHH_mASC_, *n* = 7.ELISA of IL‐1β, TNFα and IL‐6 in tissue homogenates of knee joints of mice treated as in (A). Data in (C) is displayed as floating bars with the max/min values and mean (thicker band). Biological replicates are PBS + VHH NP‐1, *n* = 4; PBS + VHH_mASC_, *n* = 4; MSU + Vehicle, *n* = 4; MSU + VHH NP‐1, *n* = 7; MSU + VHH_mASC_, *n* = 7. Data information: ^ns^ *P* > 0.05; *P < 0.05; **P < 0.005, One‐way ANOVA, multiple comparison (Tukey test). Data with non‐normal distribution were tested with Krustal‐Wallis test and multiple comparison using Dunn’s test. Outlier in (D, IL‐1β) was determined by the ROUT method and are represented with Δ.

We next tested whether VHH_mASC_ treatment could ameliorate gout after MSU inflammation is established. We thus induced disease by i.a. injection of MSU crystals and treated the mice with systemic injection of VHH_mASC_ (i.p.) after 3 h (Fig 6A). As controls, we employed both a vehicle treatment and VHH NP‐1 (Ashour *et al*, 2015), an unrelated nanobody against the nucleoprotein of influenza A virus. Strikingly, VHH_mASC_ treatment rapidly ameliorated pain sensitivity and joint swelling (6 h), when compared with the vehicle‐ or VHH NP‐1‐treated groups (Fig 6B). VHH_mASC_ treatment additionally decreased total infiltrated leukocytes and granulocytes (Fig 6C, ANOVA, *P* = 0.059 and *P* = 0.18, respectively) and the tissue concentrations of TNFα, IL‐6 and IL‐1β (Fig 6D, ANOVA, *P* = 0.056). Hence, both preventive and delayed administration of VHH_mASC_ have a strong therapeutic benefit in gout, demonstrating that a substantial fraction of MSU inflammation is driven by ASC specks exposed to the extracellular space. These findings provide the first evidence for the *in vivo* relevance of ASC specks, and support their potential as target for treatment of inflammasome‐dependent diseases. The inhibitory effect measured following systemic exposure suggests that VHH_mASC_ penetrates tissues in time to prevent the amplification of the inflammation induced by MSU crystals.

### VHH_mASC_ ameliorates antigen‐induced arthritis *in vivo*

**Figure 7:**
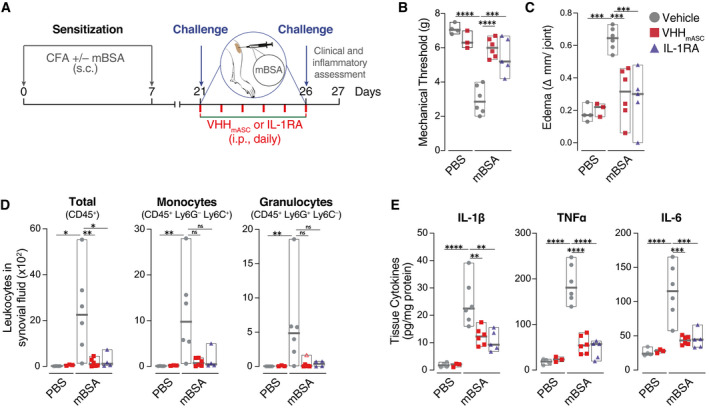
VHH_mASC_ ameliorates antigen‐induced arthritis ASchematic representation of the experimental setting used for the mBSA‐induced arthritis model. Mice were injected sub‐cutaneously (s.c.) with mBSA (500 µg/animal) in an emulsion containing 1 mg ml^−1^ Freund’s adjuvant on day 0 and day 7. Control mice received injections lacking mBSA. Mice were then immunized with an intra‐articular injection of mBSA (100 µg, right knee) on days 21 and 26. From day 21 until day 26, mice were treated daily with an intra‐peritoneal (i.p.) injection of VHH_mASC_ (5 mg kg^−1^), IL‐1RA (50 µg kg^−1^), or vehicle (PBS).B, CMechanical allodynia threshold (B) and edema (C) were evaluated on day 27. Data is displayed as floating bars with the max/min values and mean (thicker band). Biological replicates are: PBS + Vehicle, *n* = 4; PBS + VHH_mASC_, *n* = 3; mBSA + Vehicle, *n* = 6; mBSA + VHH_mASC_, *n* = 6; mBSA + IL‐1RA, *n* = 5.DFlow cytometric assessment of infiltrating total leukocytes (CD45+), granulocytes (CD45^+^ Ly6G^+^ Ly6C^−^), and inflammatory monocytes (CD45^+^ Ly6G^−^ Ly6C^+^) recovered in the synoival fluid of the knee joints of mice treated as in (A). Data is displayed as floating bars with the max/min values and mean (thicker band). Biological replicates are: PBS + Vehicle, *n* = 4; PBS + VHH_mASC_, *n* = 3; mBSA + Vehicle, *n* = 6; mBSA + VHH_mASC_, *n* = 6; mBSA + IL‐1RA, *n* = 5.EELISA of IL‐1β, TNFα and IL‐6 in tissue homogenates of knee joints of mice treated as in (A). Data is displayed as floating bars with the max/min values and mean (thicker band). Biological replicates are: PBS + Vehicle, *n* = 4; PBS + VHH_mASC_, *n* = 3; mBSA + Vehicle, *n* = 6; mBSA + VHH_mASC_, *n* = 6; mBSA + IL‐1RA, *n* = 5. Data information: ^ns^ *P* > 0.05; *P < 0.05; **P < 0.01; ***P < 0.001; ****P < 0.0001, One‐way ANOVA, multiple comparison (Tukey test). Data with non‐normal distribution were tested with Krustal–Wallis test and multiple comparison using Dunn’s test. Outliers were determined by the ROUT method and are represented with Δ.

Finally, to test the activity of VHH_mASC_ in a chronic model of inflammatory disease, we used the well‐established mBSA‐induced arthritis model (Fig 7A; Pinto *et al*, 2010, 2015). We immunized mice with a mixture of methylated bovine serum albumin (mBSA) and Freund's adjuvant (*M. tuberculosis* extract) injected sub‐cutaneously on days 0 and 7. Joint inflammation was induced by intra‐articular injection (i.a.) of mBSA on days 21 and 26. Mice were treated daily with either VHH_mASC_, IL‐1 receptor antagonist (IL‐1RA) or vehicle between challenges. On day 27, we measured clinical and inflammatory parameters (Fig 7A). As expected, i.a. treatment with mBSA induced an increased sensitivity to painful stimuli (Fig 7B), joint swelling (Fig 7C), increased infiltration of leukocytes such as monocytes and granulocytes (Fig 7D), as well as an increase concentration of cytokines (Fig 7E), when compared with vehicle or VHH_mASC_‐only conditions. In line with its activity in MSU‐induced gouty arthritis (Fig 6), VHH_mASC_ rescued the nociceptive sensitization as well as the joint swelling phenotypes induced by challenge with i.a. mBSA (Fig 7B and C). VHH_mASC_ treatment also abrogated the infiltration of pro‐inflammatory cells into the joint (Fig 7D) and strongly reduced the concentration of pro‐inflammatory cytokines in the tissue (Fig 7E). Strikingly, in all readouts, VHH_mASC_ showed a similar, or better, activity to the benchmark treatment for arthritis, anakinra (IL‐1RA, Fig 7B and E). Together, these data conclusively demonstrate the efficacy of VHH_mASC_ for the treatment of chronic arthritis *in vivo* and further suggest an important post‐pyroptotic role for ASC specks in the development of RA.

## Discussion

Research on autoimmune and inflammatory diseases has been advancing quickly, and the last years have seen major investments in novel approaches to target inflammasomes and IL‐1 cytokines. In particular, several NLRP3 inhibitors are now in clinical trials for a wide array of diseases (Carroll, 2018; Ben, 2019; Jonson & Jonson, 2021). These events follow the successful clinical application of small molecules, decoy receptors, and humanized Abs targeting IL‐1β or its signaling receptor, which became standard treatments against autoinflammatory syndromes (Kuijk *et al*, 2007; Calligaris *et al*, 2008; van der Hilst *et al*, 2016), and have also shown clinical benefits against conditions such as atherosclerosis (Ridker *et al*, 2017a), lung cancer (Ridker *et al*, 2017b), gout (Janssen *et al*, 2019), and RA (Ruscitti *et al*, 2019; Chauhan *et al*, 2020). However, a major concern surrounding blocking therapies targeting innate immunity molecules with such essential functions in host defense is that they considerably increase the risk of infections (Ottaviani *et al*, 2013; Ridker *et al*, 2017a). For example, the risk of serious opportunistic infections and cancer is increased in patients with rheumatologic diseases treated with interleukin inhibitors (Bilal *et al*, 2019). Moreover, recent off target effects of the small molecule inhibitor of NLRP3, MCC950 (or CRID3), have been reported, which may jeopardize its chances for clinical application (Kennedy *et al*, 2021).

In this study, we present camelid‐derived nanobodies as viable alternatives to target inflammasome complexes (ASC specks) that outlive pyroptotic cells and propagate inflammation in the extracellular space (Baroja‐Mazo *et al*, 2014; Franklin *et al*, 2014). Nanobodies directed against human (VHH_ASC_) and mouse ASC (VHH_mASC_) supplied to the culture medium efficiently blocked the inflammatory and prion‐like activities of ASC specks after pore formation in pyroptotic cells, while preserving initial IL‐1β release. Importantly, mouse‐specific VHH_mASC_ largely abolished the clinical manifestations of MSU‐induced gout, and AIA, providing the first evidence for the *in vivo* relevance of extracellular inflammasomes, and revealing the potential of VHH_ASC_ against acute and chronic inflammatory diseases.

ASC is an interesting therapeutic target as it plays a central role in inflammasome activation, but also displays inflammasome‐independent functions (Ellebedy *et al*, 2011; Guo & Dhodapkar, 2012; Tsuchiya *et al*, 2014; Franklin *et al*, 2017; Venegas *et al*, 2017; Friker *et al*, 2020). Recent reports have shown that extracellular ASC specks can seed amyloid aggregates such as amyloid‐β plaques at the root of Alzheimer's disease (Venegas *et al*, 2017; Friker *et al*, 2020). Hence, targeting ASC specks inside the brain using VHH_ASC_ may have clinical relevance in AD. In a mouse model of Alzheimer's disease, intra‐cranial delivery of anti‐ASC Abs prevented the ASC speck‐induced aggregation of Aβ in the hippocampus of APP/PS1 mice injected with Aβ seeds from APP/PS1 brain lysates (Venegas *et al*, 2017). Of note, no inflammatory readouts were assessed in this model, and the effect of anti‐ASC Abs in neuroinflammation remains to be determined. Other attempts to target ASC in disease have generated conflicting results. While partial benefits in brain and spinal cord injury models have been reported (de Rivero Vaccari *et al*, 2008; Desu *et al*, 2020), our previous attempt to target ASC specks using conventional Abs worsened inflammation in silica‐induced peritonitis, likely due to increased Fc‐mediated opsonization of extracellular specks (Franklin *et al*, 2014). As VHHs lack the Fc domains that interact with immune cells, they offer an alternative to target inflammasomes without engaging immune cells.

Furthermore, VHH_ASC_ are the first biologicals able to disassemble pre‐formed supramolecular organizing centers (SMOCs), the signaling organelles of the innate immune system. This feature highlight their potential as tools to investigate the structures and composition of other SMOCs, such as the Myddosome, and the poorly defined Triffosome that assemble downstream of TLR activation, or MAVS filaments formed after activation of RIG‐I‐like receptors (Kagan *et al*, 2014).

In our study, we observed that VHH_ASC_ requires cytosolic access to target intracellular inflammasomes, which is prevented by caspase‐1 inhibition or genetic ablation of GSDMD. Recent studies have placed the formation of GSDMD membrane pores upstream of some NLPR3 triggers. Indeed, human caspase‐4/5 (or mouse caspase‐11) and caspase‐8 were shown to activate GSDMD, leading to potassium efflux and downstream NLRP3 activation (Orning *et al*, 2018; Demarco *et al*, 2020; Santos *et al*, 2020). In these settings, the presence of VHH_ASC_ would most likely prevent full oligomerization of ASC specks and therefore inhibit the release of IL‐1, downstream of caspase‐1 activation. Further experiments are required to define the impact of VHH_ASC_ (or VHH_mASC_) on caspase‐8‐ or caspase‐4/11‐dependent anti‐microbial defenses.

The similarity with which VHH_ASC_ and VHH_mASC_ affect active ASC specks is informative for the contribution of ASC^CARD^ or ASC^PYD^ in the assembly of ASC specks. In fact, both CARD‐ and PYD‐mediated interactions between ASC molecules contribute to the overall structure of inflammasomes (Cai *et al*, 2014; Dick *et al*, 2016; Schmidt *et al*, 2016b). The destabilization we observed validates a role of ASC^CARD^ in the maintenance of the quaternary structure of ASC specks. Furthermore, the disruption of existing ASC specks by targeting either ASC^CARD^ or ASC^PYD^ with VHH_ASC_ or VHH_mASC_, respectively, suggests some reversibility as well as the vulnerability of both CARD‐CARD and PYD‐PYD interactions to interference, enabling both VHH_ASC_ or VHH_mASC_ to bind ASC and counteract inflammation. Our experiments targeting inflammasomes formed downstream of the NLRP3 or NLRC4 activation using either VHH_ASC_ or VHH_mASC_ demonstrate that it is sufficient to interfere with the interaction between ASC molecules rather than between ASC and the sensor. Indeed, while NLRP3 recruits ASC via PYD‐PYD interactions, NLRC4 recruits ASC via its CARD. In either case, both CARD‐ or PYD‐targeting VHH_ASC_ or VHH_mASC_, respectively, could disassemble ASC specks seeded by NLRP3 or NLRC4, indicating that ASC‐ASC interactions are critical for inflammation. In our previous work (Schmidt *et al*, 2016b), we found that VHH_ASC_ can stabilize the formation of PYD filaments when present prior to ASC oligomerization. Here, we could not detect such filaments when targeting already assembled ASC oligomers with VHH_ASC_. This could be explained by a different stoichiometry between ASC and VHH_ASC_ in our experiments. Smaller filaments would also be hard to detect with the kinetic and imaging resolution used in our experiments.

It is tempting to speculate that the use of VHH_ASC_ could also destabilize the structure of ASC oligomers in existing Aβ plaques in AD, as it does for fully functional inflammasomes. Interference with ASC oligomerization may also break the observed circle of inflammatory exacerbation fed by VHH‐accessible ASC specks. A major hurdle to target the CNS is the low penetrance of protein drugs through the blood‐brain barrier (BBB). It remains unknown whether VHH_ASC_ is able to cross the BBB and penetrate the brain. However, recently established single‐domain antibodies that cross the BBB by engaging cargo molecules of transcytosis may overcome this barrier (Gao *et al*, 2020; Wouters *et al*, 2020; Stocki *et al*, 2021). Once established, these could be fused to VHH_ASC_ to promote transport of bivalent nanobodies across the BBB. The ever‐growing toolbox of nanobody modifications such as multivalent nanobodies, Fc fusions, or fusion with albumin‐binding domains or albumin nanobodies could also be applied to VHH_ASC_ to enhance stability and half‐life in the circulation (Kontermann, 2016). Similar strategies could direct VHHs to specific cells or tissues. Whether the inhibitory function of VHH_ASC_ shown in this study remains within such a construct would need to be tested. Additionally, the *in vivo* activity of the human‐specific VHH (VHH_ASC_) would benefit from additional experiments using, for example, transgenic mice expressing human ASC.

To achieve a prolonged exposure with VHH_ASC_, the use of nanobody‐secreting bacteria strains or gene therapy may become interesting avenues for delivery in patients (Gurbatri *et al*, 2020). Nevertheless, it is important to note that nanobodies are already used in the clinic to treat thrombotic thrombocytopenic purpura and thrombosis (e.g., caplacizumab, HERCULES trial; Scully *et al*, 2019; Volker *et al*, 2020).

Gout and RA are painful and debilitating inflammatory diseases affecting the joints and other organs (Dalbeth *et al*, 2019). Current therapies include managing acute attacks using nonsteroidal anti‐inflammatory drugs (NSAIDs), corticosteroids, colchicine (gout), or disease‐modifying immunosuppressant drugs (DMARDs, RA). The growing appreciation of the NLRP3 inflammasome activation and bioactive IL‐1β release in acute gouty inflammation or in the more chronic rheumatoid arthritis has prompted studies of agents blocking the IL‐1β receptor or soluble IL‐1β (canakinumab, rilonacept, and anakinra). An NLRP3 inhibitors (Dapansutrile) has shown promising results against gout flares in early clinical trials (Kluck *et al*, 2020). While these therapies are improving acute and chronic symptoms in the clinic, our study raises the possibility of targeting post‐pyroptotic inflammasomes directly with nanobodies. Our findings suggest that the inflammatory response initiated by MSU crystals or intra‐articular antigen challenge include several rounds of amplification involving accessible ASC specks. Interestingly, VHH_mASC_ rescued the phenotype of AIA mice to the same extent than the benchmark drug anakinra. This is of significant importance because, although VHH_mASC_ and anakinra have a similar therapeutic effect in this model, their targets and mechanisms of action are different. Indeed, anakinra targets the IL‐1R and thus blocks IL‐1β‐induced downstream signaling and prevents recruitment of immune cells such as neutrophils, thus decreasing inflammation. VHH_mASC_ not only decreased swelling and pain sensitivity in AIA mice, but it also inhibited production of IL‐1β and infiltration of immune cells. This suggests that post‐pyroptotic ASC specks play an important role in the development of AIA by promoting the production of IL‐1β, probably through several rounds of amplification. Importantly, inhibiting the amplification of inflammation induced by extracellular ASC specks will still permit the initial intracellular activation of inflammasomes, essential to the immune defense against microbes. We thus describe a clinical scenario that is highly desirable for therapeutic control of inflammasome activation. Hence, nanobodies against human ASC may be a radically new alternative to other therapies that broadly target inflammasome downstream signals leaving patients with increased risks of infections.

## Materials and Methods

### Study design

In this study, we evaluated the anti‐inflammatory potency of VHH_ASC_ or VHH_mASC_ against un‐regulated inflammasomes, more specifically against extracellular inflammasomes. We first conducted *in vitro* studies using immortalized cell lines and when necessary, using primary cells. Human PBMCs were isolated from buffy coats obtained from the blood bank of the University hospital of Bonn, with consent of healthy donors and according to protocols accepted by the institutional review board of the University of Bonn (local ethics votes Lfd. Nr. 075/14). To study the activity of VHH_mASC_ *in vivo*, we used a well‐established model of MSU‐induced gout disease. These experiments were performed on male C57BL/6 wild‐type mice weighing between 18 and 22 g. They were housed in temperature‐controlled rooms (22–25°C) and given water and food *ad libitum* at the animal facility in the Department of Pharmacology of Ribeirao Preto Medical School, University of Sao Paulo, Brazil. Animal husbandry and procedures were in accordance with the guidelines of the Animal Ethics Committee of the Ribeirao Preto Medical School (241/2018). Group size were determined using the 3R principle.

### Reagents

Cell culture reagents (e.g., PBS, DMEM, RPMI, Trypsin‐EDTA) were from Gibco, Thermo Fisher Scientific. Stimuli were as follow: LPS (UltraPure, Invivogen), nigericin (Thermo Fisher Scientific), PFO (Biotrend), LFn‐BsaK, LFn‐MxiH and PA were produced in‐house as described previously (19), and uric acid used for in‐house production of MSU crystals (Sigma‐Aldrich). Inhibitors used were CRID3 (Tocris) and VX‐765 (Selleckchem). Antibodies were as follow: anti‐ASC polyclonal antibody (AL177, Adipogen, 50 µg ml^−1^) or anti‐ASC monoclonal antibody (N‐15, Santa Cruz, 1:500), anti‐Caspase‐1 p20 antibody (Casper‐1, Adipogen, 1:1,000), anti‐IL‐1β antibody (BAF401, R&D Systems, 1:1,000), and anti‐GSDMD (L60, Cell Signaling Technology, 1:1,000). DRAQ5 was from Thermo Fisher Scientific. For FACS, Abs used were anti‐CD45‐BV421, Ly6G‐FITC and Ly6C‐PerCP (BD Biosciences, 1:100). HTRF for human or mouse IL‐1β were from CisBio. ELISA kits were used to detect IL‐1β, TNFα, IL‐6 or CXCL1 (DuoSet, R&D Systems). Cell viability was measured with the Cell Titer Blue kit (CTB, Promega).

### Cell lines

THP‐1 cells (ATCC TIB‐202) expressing human ASC‐GFP and *Pycard* ^−/−^ immortalized macrophages (iMøs) expressing human ASC‐mTurquoise were generated via retroviral transduction and fluorescence‐activated cell sorting of TagGFP^+^ or mTurquoise^+^ cells, respectively. *Pycard* ^−/−^ iMøs were described previously (Franklin *et al*, 2014). Cell lines are routinely tested for contamination with Mycoplasma.

### Alpaca immunization and panning for novel VHH_mASC_

Heavy chain‐only antibodies against murine ASC (mASC) were elicited by immunizing one alpaca five times with a mixture of proteins including GFP‐mASC as a 1:1 (v/v) mixture with GERBU Adjuvant Fama (GERBU Biotechnik GmbH). GFP‐mASC was kindly provided by Liman Zhang and Hao Wu (Harvard Medical School, Boston, MA, USA). Alpaca immunizations were conducted at the research farm of preclinics GmbH (Bruchhausen‐Vilsen, Germany), according to animal procedures approved by the state of Lower Saxony. A VHH plasmid library in the M13 phagemid vector pJSC was generated as described before (Maass *et al*, 2007; Koenig *et al*, 2021). In brief, RNA from peripheral blood lymphocytes was extracted and used as a template to generate cDNA using three sets of primers (random hexamers, oligo(dT), and primers specific for the constant region of the alpaca heavy chain gene). VHH coding sequences were amplified by PCR using VHH‐specific primers, cut with AscI and NotI, and ligated into pJSC linearized with the same restriction enzymes. E. coli TG1 (Agilent) cells were electroporated with the ligation reaction and the obtained ampicillin‐resistant colonies harvested, pooled, and stored as glycerol stocks.

Mouse ASC‐specific VHHs were obtained by phage display and panning with a protocol modified from (Schmidt *et al*, 2016b). *Escherichia coli* TG1 cells containing the VHH library were infected with helper phage VCSM13 to produce phages displaying the encoded VHHs as pIII fusion proteins. Phages in the supernatant were purified and concentrated by precipitation; phages displaying GFP‐specific VHHs were removed in a depletion step with GFP immobilized to the bottom of tissue culture flasks. Phages presenting mASC‐specific VHHs were enriched using biotinylated GFP‐mASC immobilized to MyOne Streptavidin T1 Dynabeads (ThermoFisher Scientific). The obtained phages were used to infect E. coli ER2837 and subjected to a second round of panning. 96 E. coli ER2837 colonies yielded in the second panning were grown in 96‐well plates and VHH expression induced with IPTG. VHHs released into the supernatant were tested for specificity using ELISA plates coated with GFP or GFP‐mASC; bound VHHs were detected with HRP‐coupled rabbit anti‐E‐Tag antibodies (Bethyl) and the chromogenic substrate TMB. The amino acid sequence of VHH_mASC_ (JT‐01‐A09) is: QVQLVETGGG MVHPGGSLRL SCAASGFTFS EYGMTWVRQA PGKGPEWVSR INSSGGYTVY RASVKGRFTV SRDNAKNTLY LQMNSLKPED TALYYCARTT NWETRLSQGT QVTVSS.

### LUMIER assay

Protein interactions in transfected HEK 293T cells were quantified using the LUMIER assay according to a protocol modified from Schmidt *et al* (2016a). HEK 293T cells in 24‐wells were transfected with 0.5 μg bait expression vectors (pCAGGS VHH‐HA) and 0.5 μg prey expression vectors (pEXPR hASC‐Renilla, pEXPR mASC‐Renilla, pEXPR mASC^CARD^‐Renilla, or pEXPR hASC^PYD^‐Renilla, all derived from pcDNA3‐ccdB‐Renilla, a kind gift of Mikko Taipale, Susan Lindquist lab, Whitehead Institute) using Lipofectamine 2000 (Thermo Fisher Scientific). 2 days post‐transfection, cells were lysed in 120 μl LUMIER IP buffer (50 mM Hepes pH 7.9, 150 mM NaCl, 2 mM EDTA pH 8.0, 0.5% Triton X‐100, 5% glycerol, protease inhibitor cocktail (Roche)). 90 μl of the lysates were transferred to blocked LUMITRAC™ 600 plates (Greiner) coated with mouse anti‐HA.11 and incubated at 4°C for 3 h. After extensive washing steps with IP buffer, incubated wells (or 10 μl lysate) were incubated with Coelenterazine‐containing Renilla luciferase substrate mix (BioLux Gaussia Luciferase Assay Kit, New England BioLabs) and light emission quantified using a SpectraMax M3 microplate reader (Molecular Devices). Renilla luciferase activity in the immunoprecipitation samples was normalized by Renilla luciferase activity in the lysates.

### Expression and purification of recombinant VHH proteins

Recombinant VHH proteins were produced as previously described (Schmidt *et al*, 2016b; Koenig *et al*, 2021). In short, proteins were expressed in *E. coli* WK6 strain transformed with pHEN6‐based expression vectors. Cultures in terrific broth (TB) complemented with 100 µg ml^−1^ ampicillin were grown at 37°C until OD of 0.6. Protein expression was induced by addition of 1 mM IPTG and cultures grown at 30°C for 16 h. Periplasmic extracts were generated from cell pellets by osmotic shock involving incubation with TES buffer (200 mM Tris pH 8.0, 0.65 mM EDTA, 0.5 M sucrose) for 1 h, followed by dilution in 0.25× TES buffer and further incubation overnight at 4°C. Periplasmic extracts were cleared by centrifugation at 10.000 x g. Proteins were isolated using Ni‐NTA agarose beads (Qiagen), followed by size‐exclusion chromatography with a HiLoad 16/600 Superdex 75 pg column (Cytiva).

### *In vitro* generation of ASC specks

ASC specks were prepared *in vitro* as previously described (Fernandes‐Alnemri *et al*, 2007; Franklin *et al*, 2014). In short, iMøs expressing human ASC‐mTurquoise were lysed in CHAPS buffer (20 mM HEPES‐KOH, pH 7.5, 5 mM MgCl_2_, 0.5 mM EGTA, 0.1 mM PMSF and 0.1% CHAPS) by passing cells 25 times through a 20G needle. “S100” lysates were prepared at 4°C by centrifugation of the cell lysate at 20,000 *g* for 8 min followed by centrifugation at 100,000 *g* for 30 min. Lysates were finally filtered through a 0.22 µm PVDF filter spin column (Millipore). Cleared lysates were then incubated at 37°C for approximately 45 min. Specks were isolated by centrifugation at 660 *g* for 8 min, then washed with CHAPS buffer.

### Generation of bone marrow‐derived macrophages and stimulation

Bone marrow of C57BL/6 WT or ASC‐mCitrine‐expressing transgenic mice was flushed from isolated tibia and femur. Cells were then incubated for 6 days in the presence of L929 culture supernatants generated in‐house. Adherent differentiated macrophages were detached from their culture vessel using PBS supplemented with 5 mM EDTA and 2% FBS. Cells were seeded in 384‐well plates at 4 × 10^4^ cells per well. Cells were finally incubated at 37°C, 5% CO_2_ overnight, prior to stimulation. Cells were primed with 200 ng ml^−1^ LPS, then stimulated overnight with *in vitro*‐generated ASC specks (200 µg ml^−1^) that had been pre‐incubated 15 min at room temperature with either VHH_ASC_ or mutVHH_ASC_ (200 µg ml^−1^), or anti‐ASC pAb (50 µg ml^−1^). Prior to stimulation with nigericin (10 µM), PFO (250 ng ml^−1^), or the combination of the rod protein from *Burkholderia pseudomallei* (Bsak, fused to lethal factor N‐terminus) and *Bacillus anthracis* protective antigen (PA, 0.1 µg/ml/0.5 µg/ml) (Figs 3 C and D and Appendix Fig S1A and B), cells were primed with 200 ng/ml LPS for 2:30 h followed by 30‐min incubation with VHH_ASC_ or VHH_mASC_ (100 µg ml^−1^), CRID3 (50 µM) or VX‐765 (50 µM). For microscopy experiments (Fig 3E and F), cells were primed with LPS (200 ng ml^‐1^) for 2 h and then treated with the caspase‐1 inhibitor VX‐765 (50 µM) for 30 min. VHH_ASC_ or mutVHH_ASC_ (200 µg ml^−1^), or CRID3 (25 µM) were added to the cells 30 min prior to addition of PFO (250 ng ml^−1^) or nigericin (10 µM). After 2 h stimulation, cells were fixed with 4% PFA (in PBS) and nuclei stained with DRAQ5.

### Differentiation and stimulation of human M‐CSF‐differentiated macrophages

Human macrophages were generated as previously described (Rolfes *et al*, 2020). In short, PBMCs were isolated from buffy coats using a density gradient (Ficoll‐Paque Plus, GE Healthcare) followed by positive selection of monocytes using a CD14 MACS bead kit (Miltenyi Biotech). Cells were then differentiated into macrophages with 500 U ml^−1^ rhM‐CSF (Immunotools) for 3 days at 37°C, 5% CO_2_. Adherent cells were harvested using PBS complemented with 2% FBS and 2 mM EDTA and seeded into a 384‐well plate at 4 × 10^4^ cells per well, covered with a Breath‐Easy plate seal (Sigma‐Aldrich) and incubated at 37°C, 5% CO_2_ overnight. Cells were primed with 10 ng ml^−1^ LPS for 2.5 h before addition of VHH_ASC_ (100 µg ml^−1^), mutVHH_ASC_ (100 µg ml^−1^), CRID3 (50 µM) or VX‐765 (50 µM) and further incubation for 30 min. Cells were stimulated either with PFO (30 ng ml^−1^), nigericin (10 µM), ATP (2.5 mM, with pH equilibration with a final concentration of 3.75 mM NaOH), or the combination of LFn‐BsaK and PA (0.1 µg ml^−1^/0.5 µg ml^−1^) for 2 h at 37°C. For nigericin stimulation at 7 h (Appendix Fig S2), LPS‐primed cells were first stimulated with nigericin for 90 min. VHH_ASC_ (100 µg ml^−1^), mutVHH_ASC_ (100 µg ml^−1^), or CRID3 (50 µM) were then added and cells were further incubated for a total of 7 h at 37°C, 5% CO_2_. As control for the 90‐min time point, cell‐free supernatants were harvested directly after 90 min. Cell‐free supernatants were collected and IL‐1β concentration was measured by HTRF. CTB assay was performed on the remaining living cells following the manufacturer's recommendations. For activation of NLRP3 with MSU crystals, PMA‐differentiated (20 nM, 16 h) THP‐1 macrophages were pre‐treated with VHH_ASC_ and mutVHH_ASC_ (100 µg ml^−1^), CRID3 (10 µM) or VX‐765 (50 µM) 30 min prior to addition of MSU crystals (250 µg ml^−1^).

### Stimulation of other inflammasomes and challenge with ASC‐targeting VHHs

The pyrin inflammasome was stimulated for 90 min with TcdA (1 µg ml^−1^) in CD14^+^ monocytes isolated with MACS beads (Miltenyi Biotech) following the manufacturer’s instructions and primed with Pam3CysK4 for 3 h (1 µg ml^−1^). AIM2 was stimulated for 2 h with poly(dA:dT) (1 µg ml^−1^) transfected using Lipofectamine 2000 (Thermo Fisher Scientific) in PMA‐differentiated (50 ng ml^−1^, 16 h) and IFNγ‐treated (500 U ml^−1^, 16 h) THP‐1 (ASC‐GFP) cells. As previously described, in monocytes and THP‐1, VHH_ASC_ and mutVHH_ASC_ (100 µg ml^−1^) or VX‐765 (50 µM) were added 30 min prior to inflammasome stimulation. NLRP1 was stimulated for 22 h with Val‐boroPro (VbP, 30 µM) in N‐TERT keratinocytes. Here, VHHs and VX‐765 were added just before Val‐boroPro.

### Stimulation of GSDMD knockout THP‐1 cells

Doxycycline (Dox)‐inducible CRISPR‐Cas9 GSDMD knockout (GSDMD‐KO) THP‐1 cells were generously provided Seth Masters, Melbourne University. Knockout was performed as previously described (Budden *et al*, 2021). GSDMD‐KO was confirmed using SDS‐PAGE and immunoblotting. THP‐1 cells were differentiated overnight with 20 nM PMA at 37°C, 5% CO_2_. Cells were then harvested using trypsin and seeded into a 96‐well microscopy plate at a density of 5 × 10^4^ cells per well. PMA‐differentiated THP‐1 cells were allowed to adhere at least 30 min. Cells were treated with VHH_ASC_ (200 µg ml^−1^) or CRID3 (25 µM) and incubated for 30 min. Cells were then stimulated either with PFO (30 ng ml^−1^) or nigericin (10 µM) for 2 h at 37°C. Cell‐free supernatants were collected and IL‐1β concentration was measured by HTRF and LDH release was measured using a CyQUANT LDH cytotoxicity assay (ThermoFisher Scientific) following the manufacturer's recommendations. Data are represented as percentage of LDH release normalized to the maximal release (100%) from cells treated with Triton‐X100 solution (0.5% v/v in PBS) and background subtraction of signal from untreated cells (0%).

For analysis of the entry of VHH_ASC_ into the cell cytosol, CRISPR‐Cas9 GSDMD THP‐1 cells treated with doxycycline as described above were primed overnight with PMA (20 nM). Medium was replaced with imaging medium (RPMI free of phenol‐red, 10% FBS and 30 mM HEPES) before cells were treated with fluorescently‐labeled VHH_ASC_ (VHH_ASC_‐AlexaFluor647, 10 µg ml^−1^). Cells were then either left unstimulated, or stimulated with nigericin (10 µM) or PFO (25 ng ml^−1^) and imaged live on a SP8 confocal microscope (Leica) for 60 min, one time frame every 2.5 min. To detect cell death, propidium iodide (3.33 µg ml^−1^) was added to the cells before stimulation.

### Live imaging of ASC‐GFP specks

THP‐1 ASC‐GFP cells were differentiated overnight with 20 nM PMA at 37°C, 5% CO_2_. Cells were then harvested using trypsin and seeded into a 96‐well microscopy plate at a density of 5 × 10^4^ cells per well. Differentiated THP‐1 cells were allowed to adhere for 2 h. Nuclei were stained using DRAQ5 (1:1,500 dil.) and VHH_ASC_ (200 µg ml^−1^) or a combination of VHH_ASC_ and VX‐765 (50 µM) were added to the cells followed by 30 min incubation at 37°C, 5% CO_2_. PFO (25 ng ml^−1^) or nigericin (10 µM) were added to the cells followed by centrifugation at 340 *g* for 1 min. The plate was then transferred into a CellDiscoverer 7 microscope (Zeiss) already set for imaging and the inner chamber equilibrated at 37°C and 5% CO_2_. Imaging was started 7 min post‐stimulation and each frame was taken every 7 min for a total of 3h30. For each condition, a total of 8 positions within 2 wells (2 × 4 images/well) were imaged using 6 Z‐slices per image. Maximal intensity projections were generated for each image set before the number of cells and specks per field were calculated using Cell Profiler software.

### Isolation and analysis of released ASC specks from stimulated cells

THP‐1 cells expressing ASC‐GFP were differentiated overnight in the presence of 20 nM PMA. Cells were then detached from their culture dish using trypsin and transferred into 6‐well plates (3 × 10^6^ cells per well). Once cells adhered to the well, they were stimulated as indicated for 3.5 h. Cell‐free supernatants containing specks were harvested by centrifugation of the culture supernatants twice at 340 *g* for 5 min. An aliquot of the supernatant was used for FACS analysis using the gating strategy presented in Appendix Fig S1A. Specks contained in the remaining supernatants were sedimented at 5,000 *g* for 10 min and washed in HEPES buffer (20 mM Hepes pH 7.4, 150 mM NaCl, 10% glycerol). The oligomeric state of specks was analyzed by DSS cross‐linking, SDS‐PAGE and immunoblotting.

### DSS cross‐linking of ASC specks

Specks were incubated with the indicated dose of VHH_ASC_, mutVHH_ASC_ or anti‐ASC pAb for 1 h at RT. DSS was added to a final concentration of 2 mM and further incubated at RT. Cross‐linking reaction was halted by mixing with electrophoresis samples buffer (either LDS‐sample buffer complemented with reducing buffer – ThermoFisher Scientific; or provided for WES readout by ProteinSimple) and heating at 95°C for 5 min. Samples were then run on WES following manufacturer's instruction or run for Western blot on a 4–12% SDS‐PAGE (ThermoFisher Scientific – using MOPS buffer, ThermoFisher Scientific) and transferred onto a PVDF membrane (Immobilon FL, Merck‐Millipore) prior to blocking with 3% BSA (w/v) in TBS buffer and detection of ASC distribution (monomeric to oligomeric state) using a monoclonal anti‐ASC antibody (N‐15).

### MSU‐induced gout model

The experiments were performed on male C57BL/6 wild‐type animals. Animal allocation to groups was randomized. For therapeutical dosage, mice were treated with knee intra‐articular (i.a.) injection of 100 µg monosodium urate (MSU) crystal in 10 µl endotoxin‐free PBS 3 h prior to intra‐peritoneal (i.p.) injection with VHH_mASC_ (5 mg kg^−1^) or VHH52 (targeting the nucleocapsid of influenza virus, 5 mg kg^−1^) as unrelated nanobody control. For preventive dosage, mice were injected with vehicle or VHH_mASC_ (5 mg kg^−1^, i.p.) 1 h prior to knee i.a. injection of 100 µg monosodium urate (MSU) crystal in 10 µl endotoxin‐free PBS. Alternatively, VHH_mASC_ (2 mg kg^−1^) was injected i.a. together with MSU crystals. Mechanical allodynia threshold was measured using a von Frey testing device at 3 h and 6 h post‐MSU injection. Additionally, edema was evaluated by measuring the size of the joint before and 6 h after MSU injection using a digital caliper. Phenotypical readouts were conducted blinded. (Δ) represents the difference between measurement at 6 h and t‐0 h. Leukocyte infiltration was analyzed by flow cytometry using surface markers for granulocytes (CD45^+^/Ly6G^+^) and inflammatory monocytes (CD45^+^/Ly6G^−^/Ly6C^+^). Harvesting of synovial fluid was performed by four injections of 2.5 µl PBS in each joint. Lavages were then collected in 50 µl PBS placed in a 96‐well plate. Cells were then pelleted and resuspended in staining mix. Joint tissue was frozen in liquid nitrogen, crushed with pestle and homogenized in 500 µl PBS supplemented with protease inhibitors. A panel of pro‐inflammatory cytokines (IL‐1β, TNFα, and IL‐6) in the tissue homogenate were determined by ELISA (R&D Systems).

### Antigen‐induced arthritis (AIA) model

The experiment was performed as described by Pinto *et al* (2010, 2015) using male C57BL/6 wild‐type animals. Animal allocation to groups was randomized. Briefly, mice were sensitized with subcutaneous injection of 500 µg of methylated bovine serum albumin (mBSA) mixed with 100 µg Freund's adjuvant (Mycobacterium tuberculosis) on days 0 and 7. Control mice received injections of Freund's adjuvant without mBSA in saline (vehicle). Arthritis was induced by intra‐articular injection of mBSA (100 µg/cavity) into the right knee joint on days 21 and 26. During arthritis induction (day 21–26), mice received daily intra‐peritoneal injections of VHH_mASC_ (5 mg/kg), IL1RA (50 µg/kg), or saline (vehicle). After 24 h of challenge (on day 27), we assessed nociceptive mechanical threshold using electronic von Frey, and measured knee joint thickness using a caliper, as described (Pinto *et al*, 2010). Phenotypical readouts were conducted blinded. (Δ) represents the difference between the basal thickness (day 0) and the thickness on day 27. Immune cell infiltration and tissue cytokine concentrations were measured as described for the gout model.

### Statistical analysis

Experiments were run with the indicated numbers of repeats (please see figure legends). For *in vivo* experiments, mice were attributed to each group randomly at the beginning of the experiment. Wherever possible, readouts were carried out blindly by a different experimentator, especially for phenotypical readouts. For statistical analysis, data were first tested for normal (Gaussian) distribution. Normal distributed data were analyzed using one‐way ANOVA with multiple comparison (Tukey test). Non‐gaussian distributed data were first tested for the presence of outliers using the ROUT method (in figures, represented with Δ). Gaussian distribution was then again evaluated. Data with non‐normal distribution were tested with Krustal‐Wallis test and multiple comparison using Dunn's test. Statistical significance is showed as follow: ^ns^ *P* > 0.05; *P < 0.05; **P < 0.005; ***P < 0.0002; ****P < 0.0001.

## Author contributions

**Damien Bertheloot:** Formal analysis; Supervision; Investigation; Visualization; Methodology; Writing—original draft; Writing—review and editing. **Carlos WS Wanderley:** Investigation. **Ayda H Schneider:** Investigation. **Lisa DJ Schiffelers:** Investigation. **Jennifer D Wuerth:** Investigation. **Jan MP Tödtmann:** Investigation. **Salie Maasewerd:** Investigation. **Ibrahim Hawwari:** Investigation. **Fraser Duthie:** Investigation. **Cornelia Rohland:** Investigation. **Lucas S Ribeiro:** Investigation. **Lea‐Marie Jenster:** Investigation. **Nathalia Rosero:** Investigation. **Yonas M Tesfamariam:** Investigation. **Fernando Q Cunha:** Resources; Investigation; Writing ‐ review and editing. **Florian I Schmidt:** Conceptualization; Resources; Investigation; Methodology; Writing—review and editing. **Bernardo S Franklin:** Conceptualization; Resources; Data curation; Software; Formal analysis; Supervision; Funding acquisition; Validation; Investigation; Visualization; Methodology; Writing—original draft; Project administration; Writing—review and editing.

In addition to the CRediT author contributions listed above, the contributions in detail are:

Conceptualization: FIS and BSF. Methodology: DB, CWSW, JDW, FD, FQC, FIS, and BSF. Formal Analysis: DB, FIS, FQC, and BSF. Investigation: DB, CWSW, AHS, JDW, SM, JT, LS, IH, FD, CR, LSR, LJ, NR, YMT, FIS, and BSF. Visualization: DB and BSF. Writing – original draft: DB and BSF. Writing – review & editing: DB, CWSW, FQC, FIS, and BSF. Funding acquisition: BSF. Project administration: BSF. Supervision: DB, FQC, FIS, and BSF.

## Disclosure and competing interests statement

DB is a past employee and shareholder of IFM Therapeutics (unrelated to this work). FIS is a cofounder and shareholder of Dioscure Therapeutics SE as well as a consultant and shareholder of IFM Therapeutics (unrelated to this work). All other authors declare that they have no conflict of interest.

**The paper explained**

**Problem**

Inflammasomes are central signaling hubs of the innate immune system. These cytosolic sensors patrol the intracellular environment of a cell for signs of pathogen invasion of other stressors. Emerging evidence indicate that inflammasomes also have roles in the extracellular space. Activation of inflammasomes in different immune cells results in a lytic form of cell death called pyroptosis. Upon lysis, pyroptotic cells spill their intracellular content, including these multimeric inflammasome platforms, into the extracellular space. Extracellular ASC specks have been reported in the circulation and in inflamed tissues in different disease states. However, little is known about their contribution to the development of inflammation and disease.

**Results**

Using camelid‐derived single‐domain antibodies (nanobodies, VHHs), we demonstrate that extracellular ASC specks that remain after the demise of pyroptotic cells mediate a substantial part of the inflammation in two models of arthritis. VHH directed against ASC (VHH_ASC_) target and disrupt inflammasome complexes (ASC specks) in pyroptotic cells preventing their inflammatory and prion‐like extracellular functions. Furthermore, we show that VHH_ASC_ can only access cytosolic ASC specks through membrane pores formed after the demise of inflammasome‐activated cells, thus, without compromising early cytokine production (IL‐1β), important for host defense.

**Impact**

Biotherapies aimed against pro‐inflammatory cytokines, such as IL‐1β, are currently used against Rheumatoid arthritis (RA) and other inflammasome‐related diseases. Given its essential role in host defense, IL‐1‐blockade increases the risk of serious infections in patients. Hence, the use of nanobodies against the inflammasome adaptor ASC is an attractive therapeutic alternative to overcome immunosuppression as it preserves inflammasome‐activation and IL‐1β production, while blocking the extracellular functions of inflammasomes that persist in inflamed tissues. Nanobodies targeting ASC are thus tailored to modulate the host response to inflammation and limit chronic disease.

## Supporting information



Appendix

Expanded View Figures PDF

Movie EV1

Movie EV2

Movie EV3

Movie EV4

Movie EV5

Movie EV6

Source Data for Expanded View

Source Data for Figure 1

Source Data for Figure 2

Source Data for Figure 4

Source Data for Figure 5

## Data Availability

This study includes no dataset requiring the deposition of primary data into a public database.
