# Supplementary material for: Nanobodies dismantle post‐pyroptotic ASC specks and counteract inflammation in vivo
Source: EMBO Mol Med. 2022 Apr 19;14(6):e15415. doi: 10.15252/emmm.202115415 (PMC9174887; doi:10.15252/emmm.202115415)
Supplement: Supplementary file 1 — Appendix [file EMMM-14-e15415-s001.pdf]

Single-domain antibodies against ASC disassemble post-pyroptotic inflammasomes and reveal their role in inflammatory diseases

Damien Bertheloot[1], Carlos W. de Souza Wanderley[2,3], Ayda Henriques Schneider[2,3], Lisa Schiffelers[1], Jennifer D. Wuerth[1], Jan Tödtmann[4], Salie Maasewerd[1], Ibrahim Hawwari[1], Fraser Duthie[1], Cornelia Rohland[1], Lucas S. Ribeiro[1], Lea Jenster[1], Nathalia Rosero[1], Yonas Mehari Tesfamariam[1], Fernando Q. Cunha[2,3], Florian I. Schmidt[1, 4] and Bernardo S. Franklin[1]

Affiliations:

[1]Institute of Innate Immunity, Medical Faculty, University of Bonn, 53127 Bonn, NRW, Germany.

[2]Center for Research in Inflammatory Diseases (CRID), Ribeirao Preto Medical School, University of Sao Paulo, Brazil.

[3]Department of Pharmacology, Ribeirao Preto Medical School, University of Sao Paulo, Brazil.

[4]Core Facility Nanobodies, Medical Faculty, University of Bonn, 53127 Bonn, Germany

Correspondence: d.berthellot@uni-bonn.de, fschmidt@uni-bonn.de, franklin@uni-bonn.de

Running Title: VHH_{ASC} targets extracellular ASC specks.

Appendix Table of Contents

1. **Appendix Figures S1 -** VHH_{ASC} disrupts released ASC specks
2. **Appendix Figures S2 -** Maximum IL-1β secretion is reached early in inflammasome activation of primary human macrophages
3. **Appendix Figures S3 -** VHH_{mASC} ameliorates MSU gouty inflammation
4. **Appendix Figures S4 -** Local VHH_{mASC} ameliorates MSU gouty inflammation

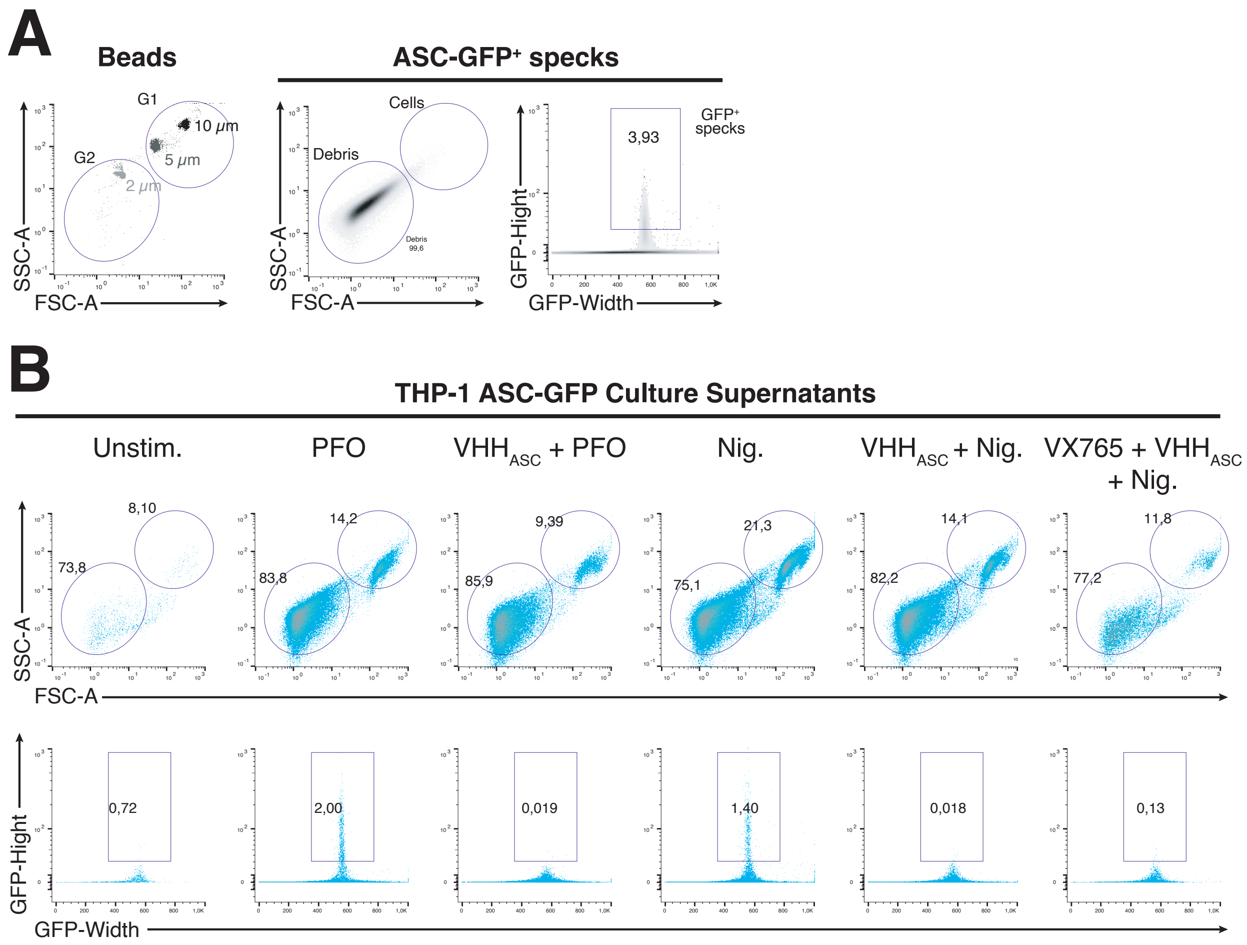

Appendix Figure S1. VHH$_{ASC}$ disrupts released ASC specks (**A**) Flow cytometry plots representing the method used to analyse GFP^{+} specks from cell-reduced supernatants. Beads of 10, 5 or 2 μm diameter were used as size "calibrator". (**B**) Example of flow cytomerty analysis of supernatants from PMA-differentiated THP-1 cells expressing ASC-GFP and left untreated, or pre-incubated with VHH$_{ASC}$ (100 μg ml^{-1}), or VX-765 (50 μM) for 30 min before activation with PFO (30 ng ml^{-1}) or nigericin (10 μM) for 3.5h.

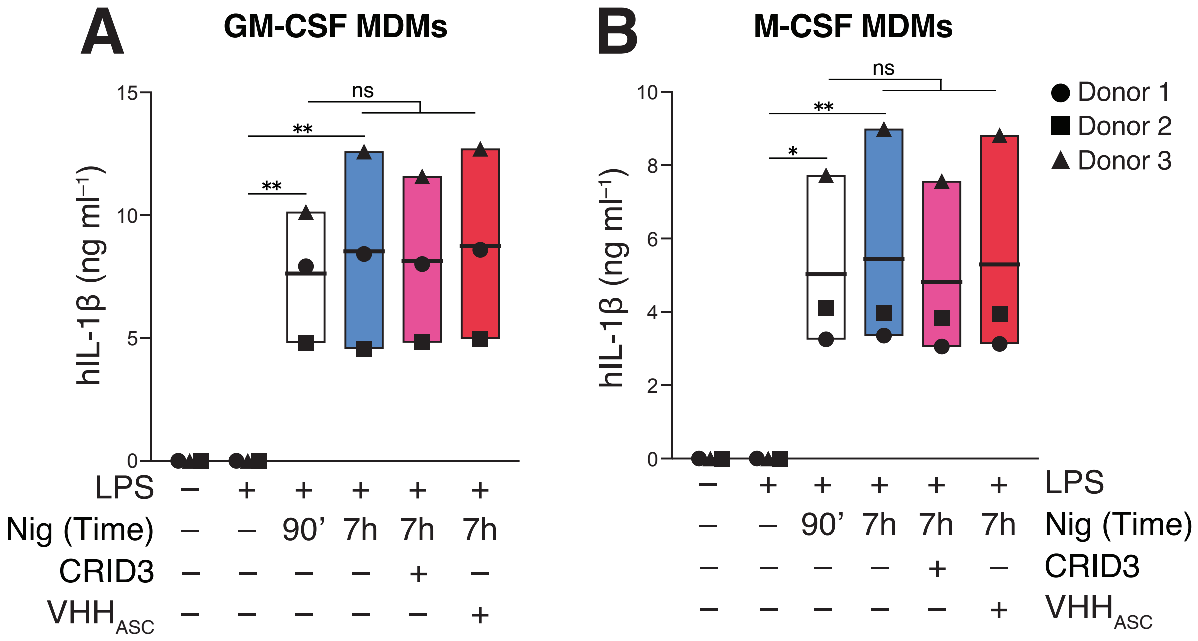

Appendix Figure S2. Maximum IL-1β secretion is reached early in inflammasome activation of primary human macrophages. (**A**) IL-1β levels measured in cell-free supernatants of primary human macrophages (hMDMs) at 90 min (90’) or 7 hours (7h) of inflammasome activation. Cells were generated by differenciating primary human monocytes with GM-CSF (**A**) or M-CSF (**B**) from three blood donors. hMDMs were primed with LPS (10 ng ml^{-1}) for 3 hours, followed by stimulation with nigericin (10 μM) for the indicated time. In some conditions, CRID3 (2.5 μM) or VHH_{ASC} (200 μg ml^{-1}) was added to cells after 90 min of nigericin-stimulation and IL-1β levels were assessed at 7 hours post stimulation. Data is from two experiment with a total of three blood donors. Each symbol represents one donor. ns, p > 0.05; *, p<0.05; **, p<0.005, One-way ANOVA, multiple comparison (Tukey test).

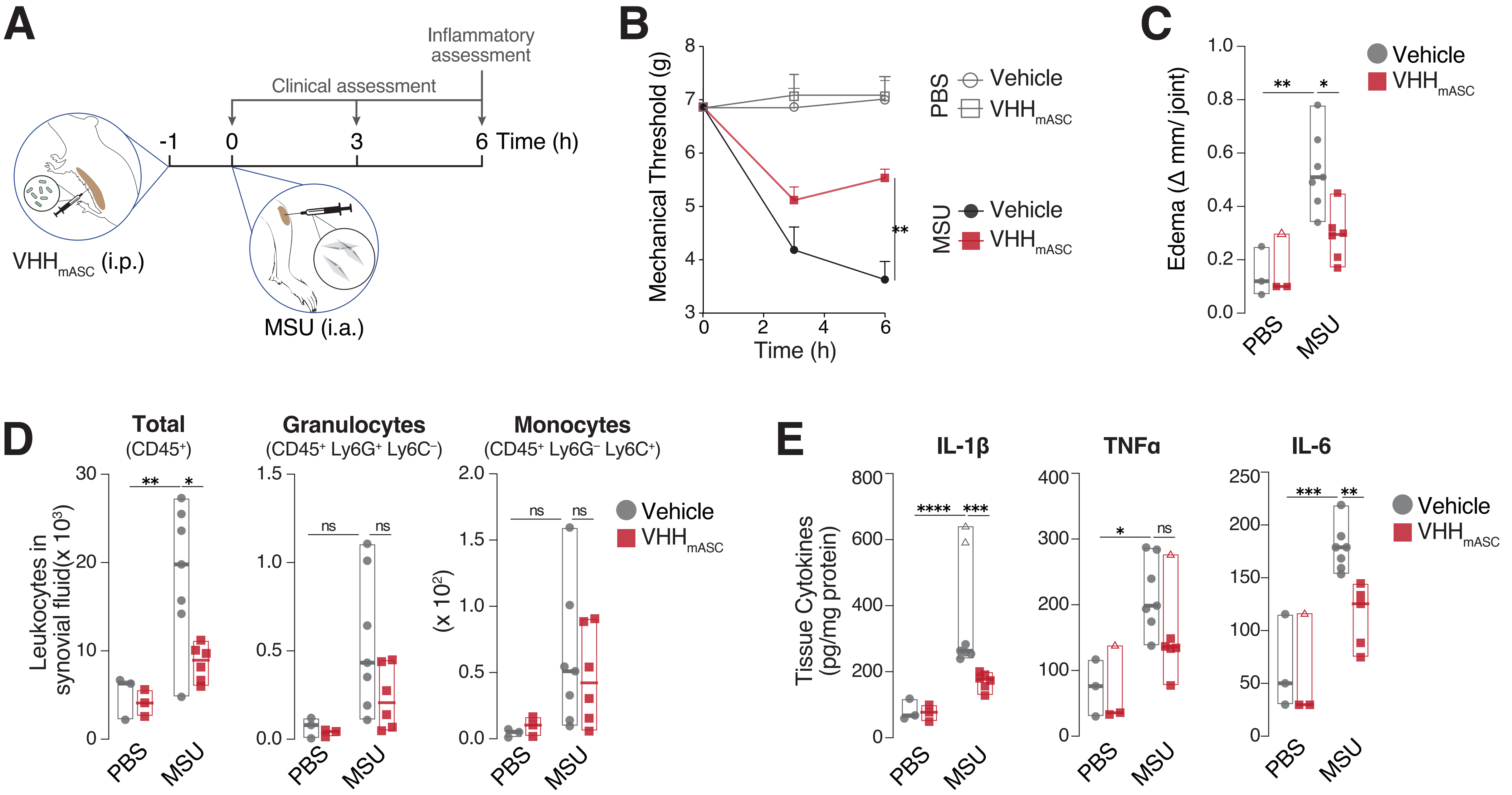

Appendix Figure S3. VHH$_{mASC}$ ameliorates MSU gouty inflammation. (A) Schematic representation of the experimental setting used for the MSU-gout model. Mice were injected intraperitonially (i.p.) with VHH$_{mASC}$ (5 mg/kg) or vehicle (PBS) 1h prior to the intra-articular (i.a) injection of 100 μg of monosodium urate (MSU) crystals into the knee. Mechanical allodynia threshold (**B**) was evaluated at 3 and 6 hour post-challenge and edema (**C**) was evaluated at 6 hour post-challenge. (**D**) Flow cytometric assessment of infiltrating total leukocytes (CD45$^+$), granulocytes (CD45$^+$ Ly6G$^+$ Ly6C$^-$), and inflammatory monocytes (CD45$^+$ Ly6G$^-$ Ly6C$^+$) recovered in the synoival fluid of the knee joints of mice treated as in (A). (**E**) ELISA of IL-1β, TNFα and IL-6 in tissue homogenates of knee joints of mice treated as in (A). ns, $p > 0.05$; *, $p<0.05$; **, $p<0.005$; ***, $p<0.0002$, One-way ANOVA, multiple comparison (Tukey test). Data with non-normal distribution were tested with Krustal-Wallis test and multiple comparison using Dunn's test. Outliers were determined by the ROUT method and are represented with Δ.

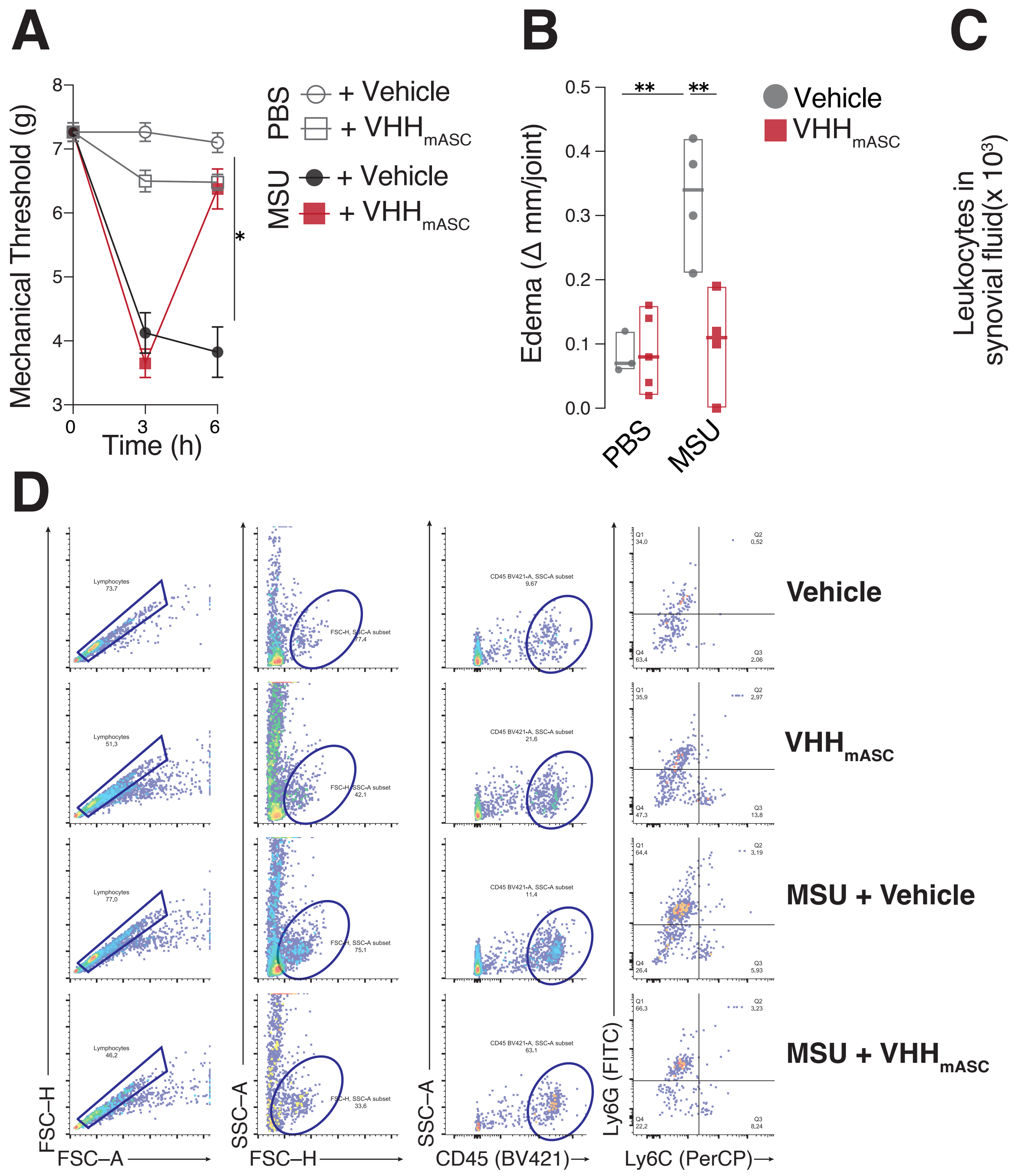

Appendix Figure S4. Local VHH$_{mASC}$ ameliorates MSU gouty inflammation. Wild-type C57BL/6 mice were injected intra-articularly into the knee joint with VHH$_{mASC}$ (2 mg/kg), or Vehicle together with 100 μg monosodium urate (MSU) crystals dissolved in 10 μL endotoxin free PBS. Mechanical allodynia threshold (**B**) was evaluated at 3 and 6 hour post-challenge and edema (**C**) was evaluated at 6 hour post-challenge. (**C**) Flow cytometric quantification of total infiltrating leukocytes(CD45$^+$), granulocytes (CD45$^+$ Ly6G$^+$ Ly6C$^-$), and inflammatory monocytes (CD45$^+$ Ly6G$^-$ Ly6C$^+$) recovered in the synoival fluid of the knee joints of mice treated as in **A-B**. (**D**) Gating strategy used for flow cytometric quantification of infiltrating cells showed in **Fig. 5D**.
