# Supplementary material for: Nanobodies dismantle post‐pyroptotic ASC specks and counteract inflammation in vivo
Source: EMBO Mol Med. 2022 Apr 19;14(6):e15415. doi: 10.15252/emmm.202115415 (PMC9174887; doi:10.15252/emmm.202115415)
Supplement: Supplementary file 2 — Expanded View Figures PDF [file EMMM-14-e15415-s004.pdf]

Expanded View Figures

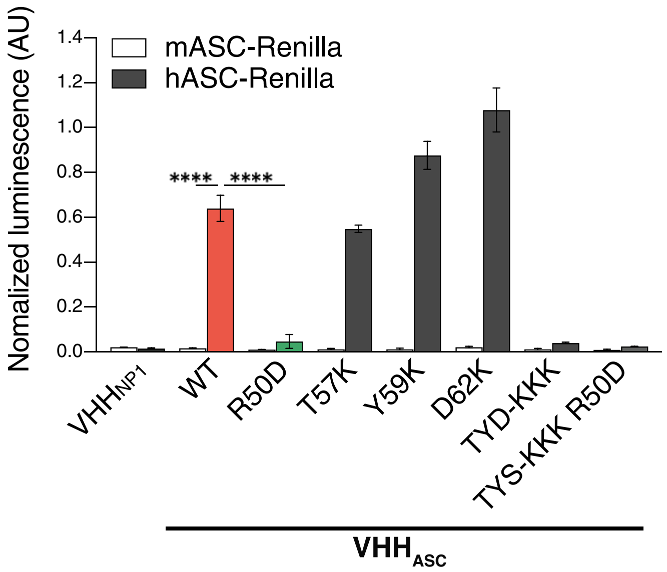

Figure EV1. Binding activity and species specificity of ASC VHHs.

Lysates of HEK 293T cells transiently expressing HA-tagged VHH_{ASC} (wild-type or point mutants), an unrelated control (VHH_{NP1}) and the indicated bait proteins fused to Renilla luciferase were used to immunoprecipitate VHHs with immobilized anti-HA antibody. Renilla luciferase activity of the co-immunoprecipitated proteins was measured and normalized to the input luciferase. Data represents mean values ± SEM from three independent experiments. ****$P < 0.0001$, One-way ANOVA, multiple comparison (Tukey test). 'TYD-KKK' indicates the triple mutant T57K, Y59K, and D62K.

© 2022 The Authors

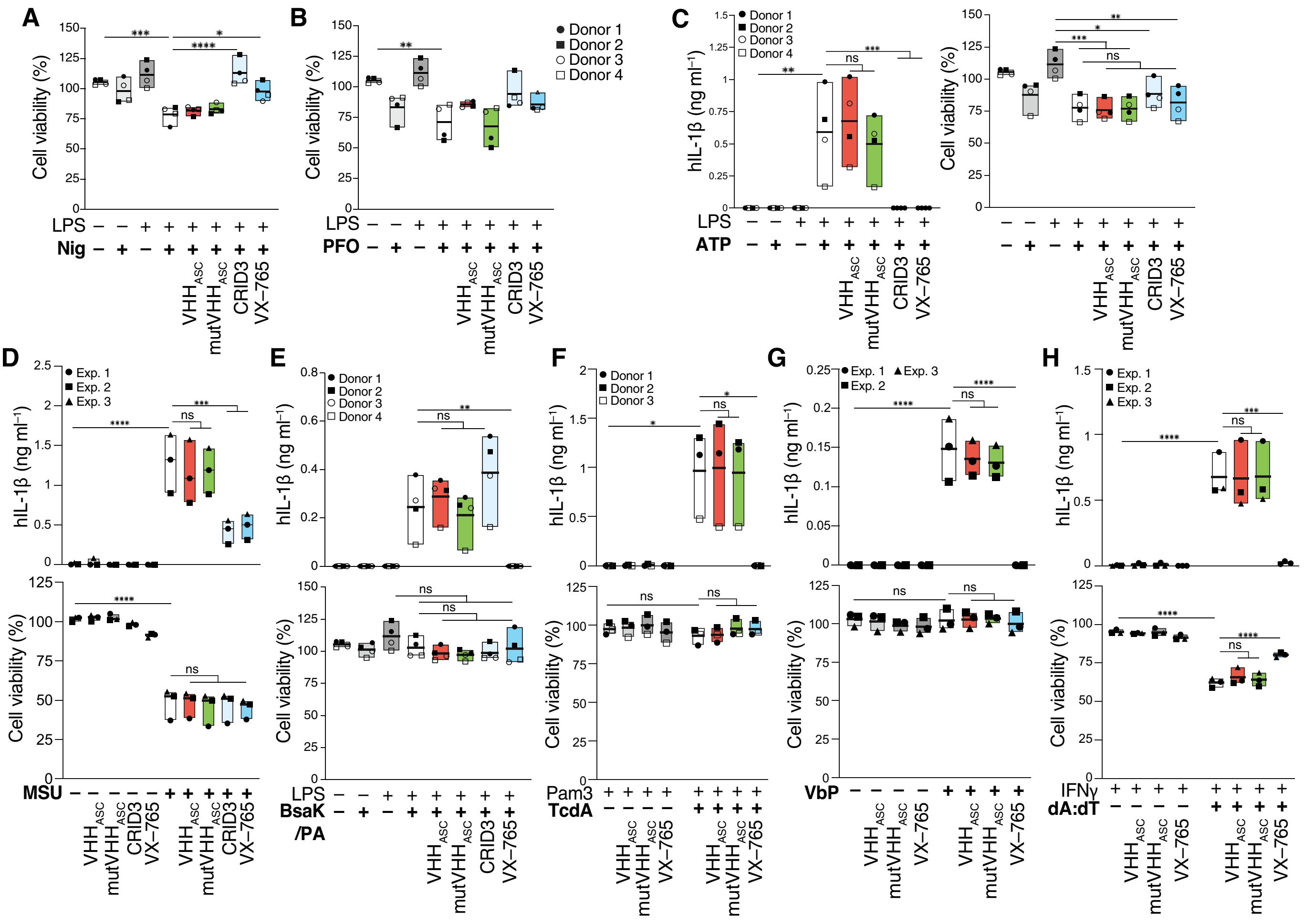

Figure EV2. VHH_{ASC} preserves endogenous inflammasome activation in human living cells.

A, B Cell viability (CTB) assay on LPS-primed primary human macrophages that were left untreated, or pre-incubated with VHH_{ASC} or $mutVHH_{ASC}$ (100 μg ml^{-1}), CRID3 (50 μM) or VX-765 (50 μM) for 30 min before being activated with (A) nigericin (10 μM), or (B) PFO (30 ng ml^{-1}) for 2 h. Data is from the experiments displayed in Fig 3A and B.

C Human IL-1β (hIL-1β) concentrations in cell-free supernatants (left), and cell viability assay (right) of LPS-primed primary human macrophages that were incubated with VHH_{ASC} or $mutVHH_{ASC}$ (100 μg ml^{-1}), CRID3 (50 μM) or VX-765 (50 μM) for 30 min before being stimulated with 2.5 mM ATP.

D hIL-1β concentrations in cell-free supernatants (top), and cell viability assay (bottom) of PMA-differentiated THP-1 cells treated with VHH_{ASC} or $mutVHH_{ASC}$ (100 μg ml^{-1}), CRID3 (10 μM) or VX-765 (50 μM) for 30 min before 4.5 h stimulation with 250 μg ml^{-1} MSU crystals.

E hIL-1β concentrations in cell-free supernatants (top), and cell viability assay (bottom) of LPS-primed primary human macrophages that were incubated with VHH_{ASC} or $mutVHH_{ASC}$ (100 μg ml^{-1}), CRID3 (50 μM) or VX-765 (50 μM) for 30 min before being stimulated with 0.1 μg ml^{-1}/0.5 μg ml^{-1} mixture of LFn-BsaK and PA for 2 h.

F hIL-1β concentrations in cell-free supernatants (top), and cell viability assay (bottom) of Pam3CysK4-primed (1 μg ml^{-1}) primary human $CD14^{+}$ monocytes that were incubated with VHH_{ASC} or $mutVHH_{ASC}$ (100 μg ml^{-1}), or VX-765 (50 μM) for 30 min before being stimulated with 1 μg ml^{-1} TcdA.

G hIL-1β concentrations in cell-free supernatants (top), and cell viability assay (bottom) of keratinocyte cells (N-TERT) that were treated with VHH_{ASC} or $mutVHH_{ASC}$ (100 μg ml^{-1}), or VX-765 (50 μM), then directly stimulated with 30 μM Val-boroPro (VbP) for 22 h.

H hIL-1β concentrations in cell-free supernatants (top), and cell viability assay (bottom) of PMA-differentiated THP-1 cells treated with IFNγ (500 U ml^{-1}) for 16 h and that were incubated with VHH_{ASC} or $mutVHH_{ASC}$ (100 μg ml^{-1}), CRID3 (10 μM) or VX-765 (50 μM) for 30 min before 2 h stimulation with 1 μg ml^{-1} poly(dA:dT) in complex with Lipofectamine 2000.

Data information: Data is representative of either two independent experiments, each run with one to two donors (A–C, E, F, 3 or 4 donors in total) or at least 3 independent experiments (D, G, H). Each symbol represents one donor or independent experiment. $^{ns}P > 0.05$; $*P < 0.05$; $**P < 0.005$; $***P < 0.0002$; $****P < 0.0001$, One-way ANOVA, multiple comparison (Tukey test). Data is displayed as floating bars with the max/min values and mean (thicker band).

© 2022 The Authors

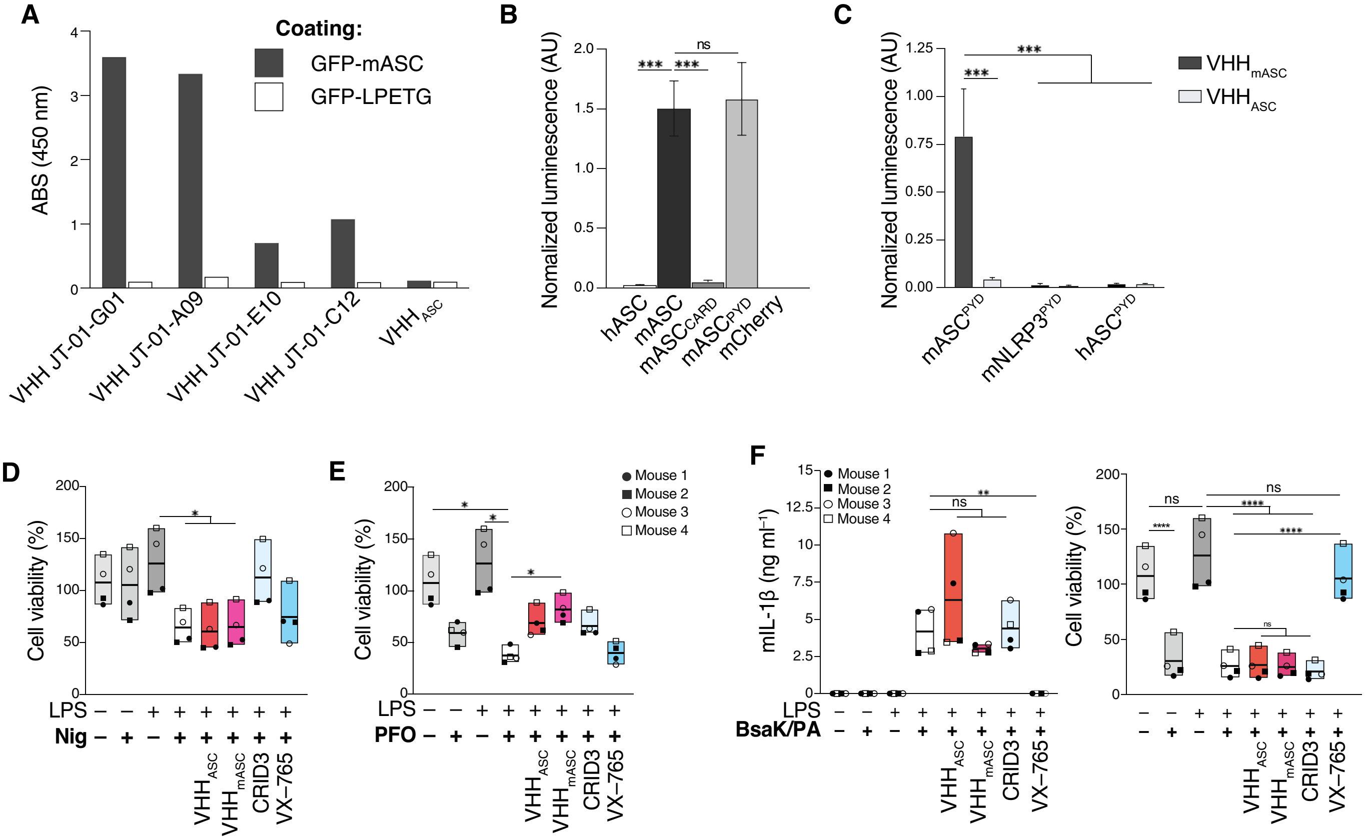

Figure EV3. VHH_{mASC} is specific to ASC^{PYD} but retains the same activity as VHH_{ASC}.

A Specificity of different single-domain antibodies (VHHs) probed by ELISA. Recombinant murine ASC as an eGFP fusion or eGFP alone (GFP-LPETG) as control, were coated onto ELISA plates at 1 μg ml^{-1}/well. Wells were incubated with HA-tagged VHHs (100 nM), anti-HA-tag mouse mAb coupled to HRP, and the HRP substrate TMB. Binding was quantified by measuring the absorbance at 450 nm.

B, C Lysates of HEK 293T cells transiently expressing HA-tagged VHH_{mASC} or VHH_{ASC} and the indicated bait proteins fused to Renilla luciferase were used to immunoprecipitate VHHs with immobilized anti-HA antibody. Renilla luciferase activity of the co-immunoprecipitated proteins was measured and normalized to the input luciferase. Data represents mean values ± SEM from three independent experiments.

D, E Cell viability (CTB) assay on LPS-primed (200 ng ml^{-1}) primary mouse BMDMs that were left untreated, or pre-incubated with VHH_{ASC} or VHH_{mASC} (100 μg ml^{-1}), CRID3 (50 μM) or VX-765 (50 μM) for 30 min before being activated with (D) nigericin (10 μM), or (E) PFO (250 ng ml^{-1}) for 2 h. Data is from the experiments displayed in Fig 3C and D. Data is displayed as floating bars with the max/min values and mean (thicker band).

F Mouse IL-1β (mIL-1β) concentrations in cell-free supernatants (left), and cell viability assay (right) of LPS-primed mouse BMDMs incubated with VHHs, CRID3 or VX–765, before stimulation with 0.1 μg ml^{-1}/0.5 μg ml^{-1} mixture of LFn-BsaK and PA for 2 h. Each symbol represents one mouse. Data is displayed as floating bars with the max/min values and mean (thicker band).

Data information: $^{ns}P > 0.05$; $^{***}P < 0.001$; $^{****}P < 0.0001$, Two-way (A, C) or One-way (B, D–F) ANOVA, multiple comparison (Tukey test).

© 2022 The Authors

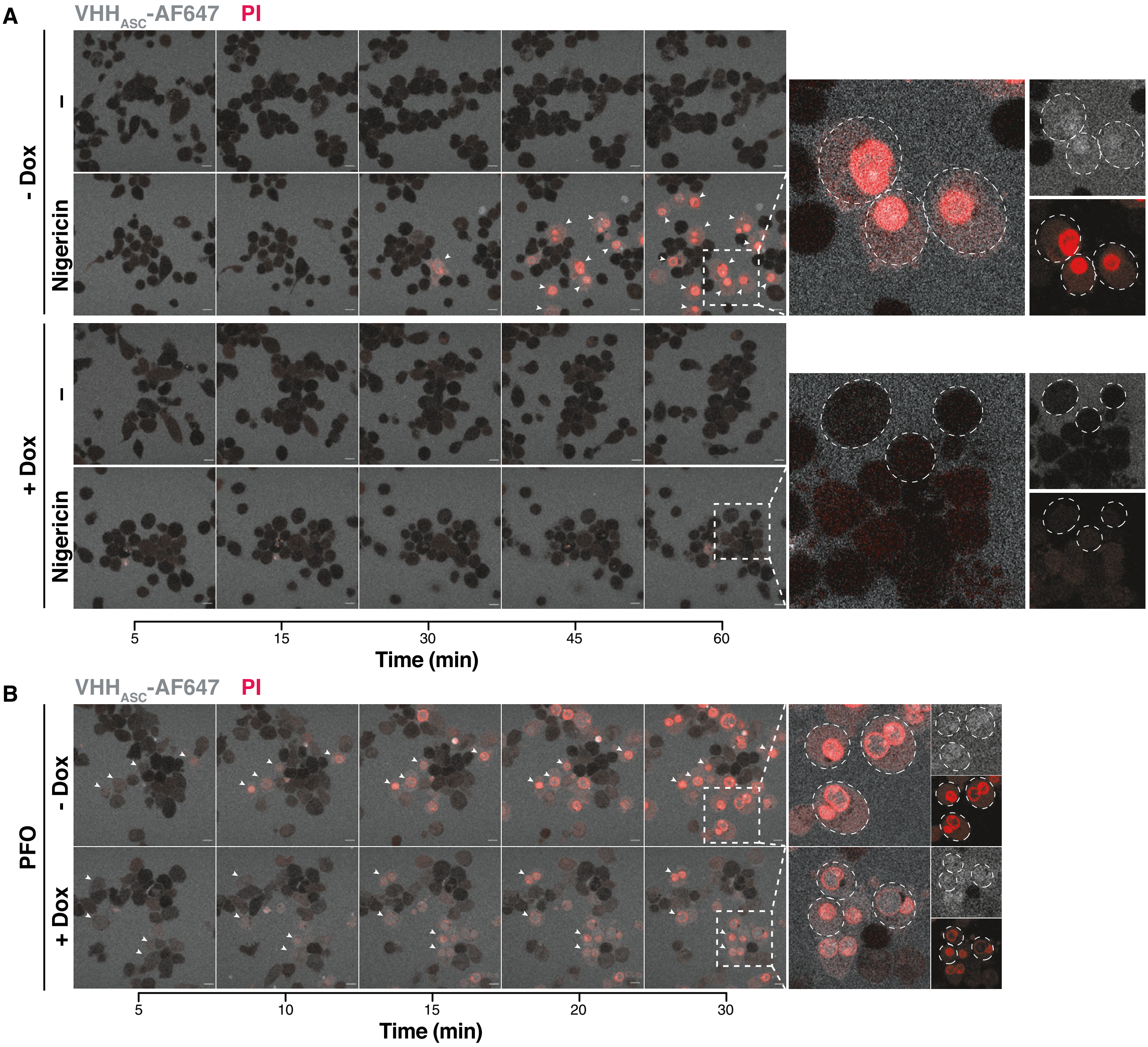

Figure EV4. Contribution of GSDMD in the effect of VHHASC on the internalization of VHH$_{ASC}$ in cells stimulated with PFO or nigericin.

THP-1 cells expressing a Dox-inducible CRISPR-Cas9 cassette targeting GSDMD gene were left untreated (–) or treated with 1 μg ml^{-1} Dox for one or two cycles of 72 h (1×, or 2× respectively).

A, B Confocal microscopy images of GSDMD competent (–Dox) or GSDMD-KO (+Dox) cells primed with PMA and that were either left untreated or treated with (A) nigericin (10 μM) or (B) PFO (30 ng ml^{-1}) for the indicated periods of time and in the presence of AlexaFluor647-labeled VHH$_{ASC}$ (VHH$_{ASC}$-AF647, 10 μg ml^{-1}, blue) and propidium iodide (PI, 3.33 μg ml^{-1}, red). White arrows indicate of cells with intracellular VHH$_{ASC}$-AF647 signal. Dashed circles define boundaries of representative cells. Data is from one representative out of three independent experiments. Scale bar: 10 μm. Also see Movie EV1.

© 2022 The Authors

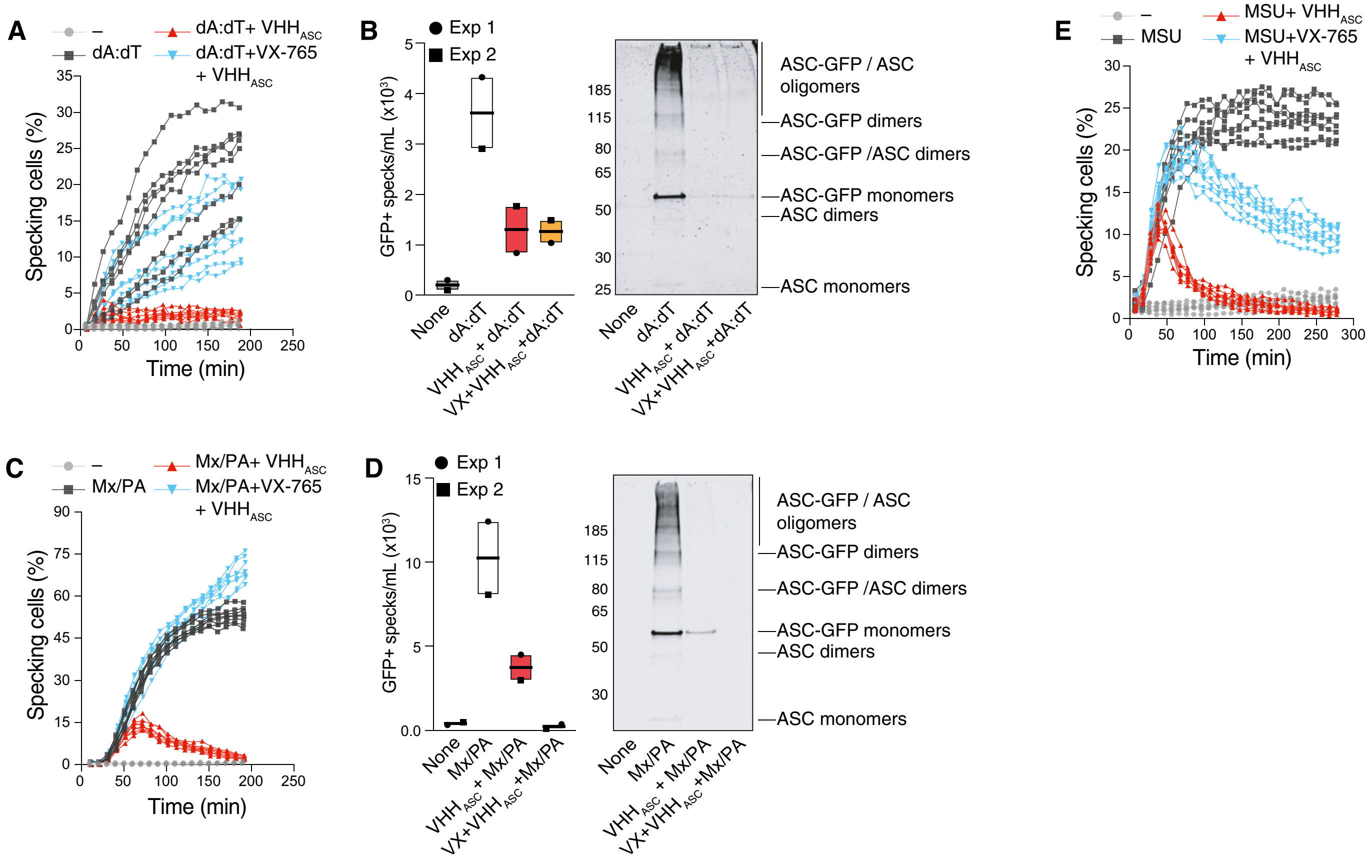

Figure EV5. VHH$_{ASC}$ disrupts post-pyroptotic ASC specks.

A PMA-differentiated (50 ng ml^{-1}) THP-1 ASC-GFP cells were treated with IFNγ (500 U ml^{-1}) for 16 h. Then, cells were pre-incubated with VHH$_{ASC}$ (100 μg ml^{-1}), or VX-765 (50 μM) for 30 min before being activated with poly(dA:dT) (dA:dT, 1 μg ml^{-1}) and imaged live with a CellDiscoverer 7 microscope as described in Fig 4 over 3 h. Data is a graphic representation of the percent of specking cells over time. Maximal intensity projections from Z-stacks were generated for each image set (8 images per condition) before the number of cells and specks per field were calculated using CellProfiler. Also see Movie EV3.

B Flow cytometric quantification (left), or western-blot (right, following DSS cross-linking) assessement of GFP^{+} specks in the supernatants of cells pre-treated with VHH$_{ASC}$ or VX-765 and then activated with poly(dA:dT) for 3.5 h. Data in left panel is displayed as floating bars with the max/min values and mean (thicker band).

C Flow cytometric quantification or western-blotting of GFP+ specks released by PMA-differentiated THP-1 ASC-GFP cells pre-incubated with VHH$_{ASC}$ (100 μg ml^{-1}), or VX-765 (50 μM) for 30 min before stimulation with LFn-MxiH/PA (100 ng ml^{-1}/1 μg ml^{-1}) for 3 h and imaged live with a CellDiscoverer 7 microscope. Also see Movie EV4.

D GFP^{+} specks detected by flow cytometry (left) or western-blot (right, following DSS cross-linking) in the supernatants of cells pre-treated with VHH$_{ASC}$ or VX-765 and then activated with LFn-MxiH/PA for 3.5 h. Data in left panel is displayed as floating bars with the max/min values and mean (thicker band).

E PMA-differentiated THP-1 ASC-GFP cells were pre-incubated with VHH$_{ASC}$ (100 μg ml^{-1}), or VX-765 (50 μM) for 30 min before stimulation with MSU crystals (250 μg ml^{-1}) for 4.5 h and imaged live with a CellDiscoverer 7 microscope. Imaging and quantifications were done as in (A). Also see Movie EV5.

Source data are available online for this figure.

© 2022 The Authors
