# Supplementary material for: Nanobodies dismantle post‐pyroptotic ASC specks and counteract inflammation in vivo
Source: EMBO Mol Med. 2022 Apr 19;14(6):e15415. doi: 10.15252/emmm.202115415 (PMC9174887; doi:10.15252/emmm.202115415)
Supplement: Supplementary file 3 — Movie EV1 [file EMMM-14-e15415-s012.zip › Movie EV1_Legend.docx]

**Single-domain antibodies against ASC disassemble post-pyroptotic inflammasomes and reveal their role in inflammatory diseases**

Damien Bertheloot^1^, Carlos W. de Souza Wanderley^2,3^, Ayda Henriques Schneider^2,3^, Lisa Schiffelers^1^, Jennifer D. Wuerth^1^, Jan Tödtmann^4^, Salie Maasewerd^1^, Ibrahim Hawwari^1^, Fraser Duthie^1^, Cornelia Rohland^1^, Lucas S. Ribeiro^1^, Lea Jenster^1^, Nathalia Rosero^1^, Yonas Mehari Tesfamariam^1^, Fernando Q. Cunha^2,3^, Florian I. Schmidt^1, 4^ and Bernardo S. Franklin^1^

**Affiliations:**

^1^Institute of Innate Immunity, Medical Faculty, University of Bonn, 53127 Bonn, NRW, Germany.

^2^Center for Research in Inflammatory Diseases (CRID), Ribeirao Preto Medical School, University of Sao Paulo, Brazil.

^3^Department of Pharmacology, Ribeirao Preto Medical School, University of Sao Paulo, Brazil.

^4^Core Facility Nanobodies, Medical Faculty, University of Bonn, 53127 Bonn, Germany

Correspondence: [d.berthellot@uni-bonn.de](mailto:d.berthellot@uni-bonn.de), [fschmidt@uni-bonn.de](mailto:fschmidt@uni-bonn.de), [franklin@uni-bonn.de](mailto:franklin@uni-bonn.de)

**Running Title:** VHH_ASC_ targets extracellular ASC specks.

**Movie EV1: Contribution of GSDMD in the effect of VHHASC on the internalization of VHH_ASC_ in cells stimulated with PFO or nigericin.**

THP-1 cells expressing a Dox-inducible CRISPR-Cas9 cassette targeting GSDMD gene were treated with 1 µg ml-1 Dox for one or two cycles of 72 h (1x, or 2x respectively). As control, cells were left untreated and cultured in parallel (–). Confocal microscopy images of GSDMD competent (–Dox) or GSDMD-KO (+Dox) cells primed with PMA and that were either left untreated or treated with (A) nigericin (10 µM) or (B) PFO (30 ng ml–1) for the indicated periods of time and in the presence of AlexaFluor647-labeled VHH_ASC_ (VHH_ASC_-AF647, 10 µg ml^–1^, grey) and propidium iodide (PI, 3.33 µg ml^–1^, red). Scale bars represent 10 µm and time stamps represent time after addition of PFO or nigericin (h:min).
