# Supplementary figures and images for: Nanobodies dismantle post‐pyroptotic ASC specks and counteract inflammation in vivo
Source: EMBO Mol Med. 2022 Apr 19;14(6):e15415. doi: 10.15252/emmm.202115415 (PMC9174887; doi:10.15252/emmm.202115415)

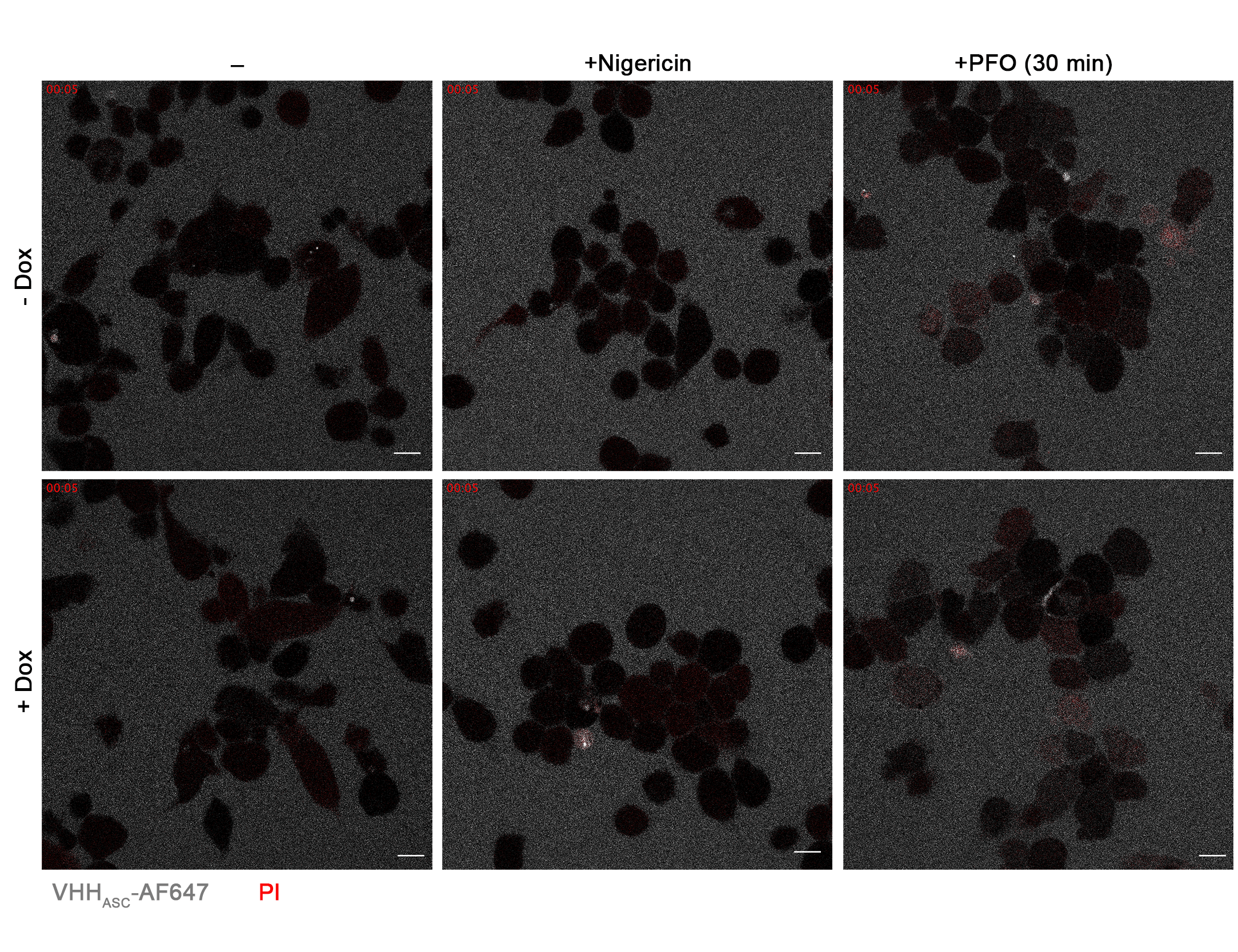

Supplement: Supplementary file 3 — Movie EV1 [file EMMM-14-e15415-s012.zip › Movie EV1.gif]

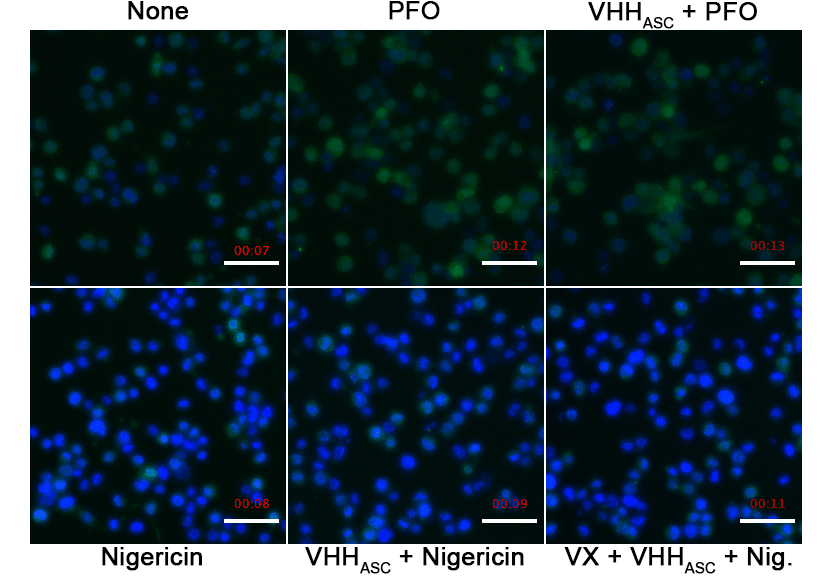

Supplement: Supplementary file 4 — Movie EV2 [file EMMM-14-e15415-s008.zip › Movie EV2.gif]

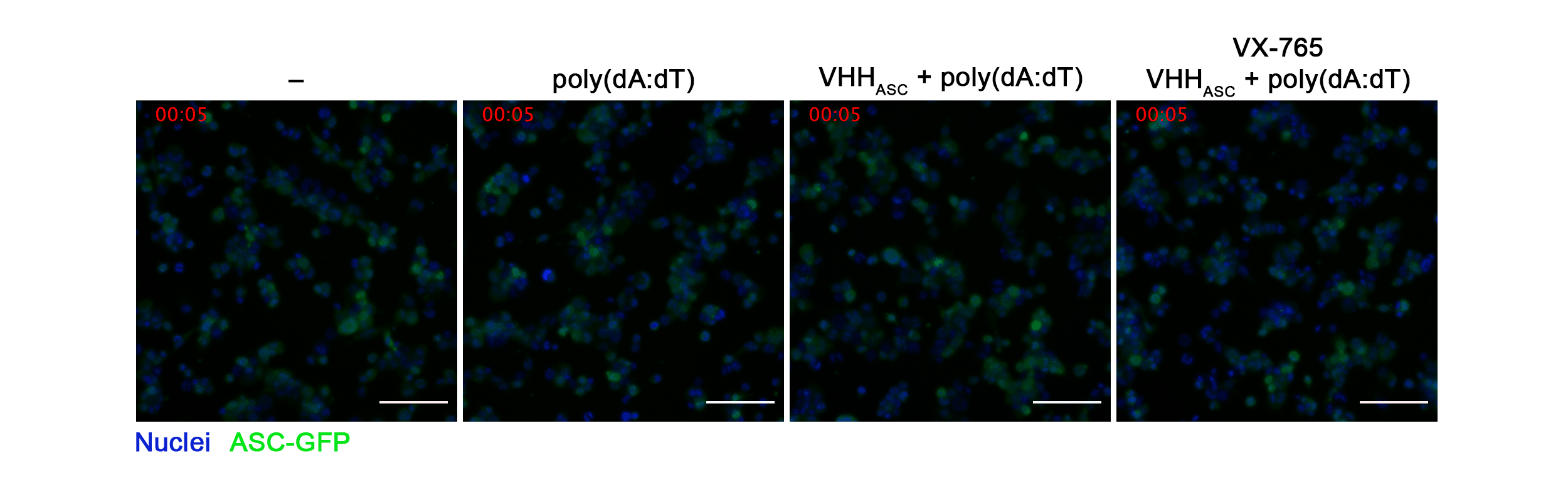

Supplement: Supplementary file 5 — Movie EV3 [file EMMM-14-e15415-s006.zip › Movie EV3.gif]

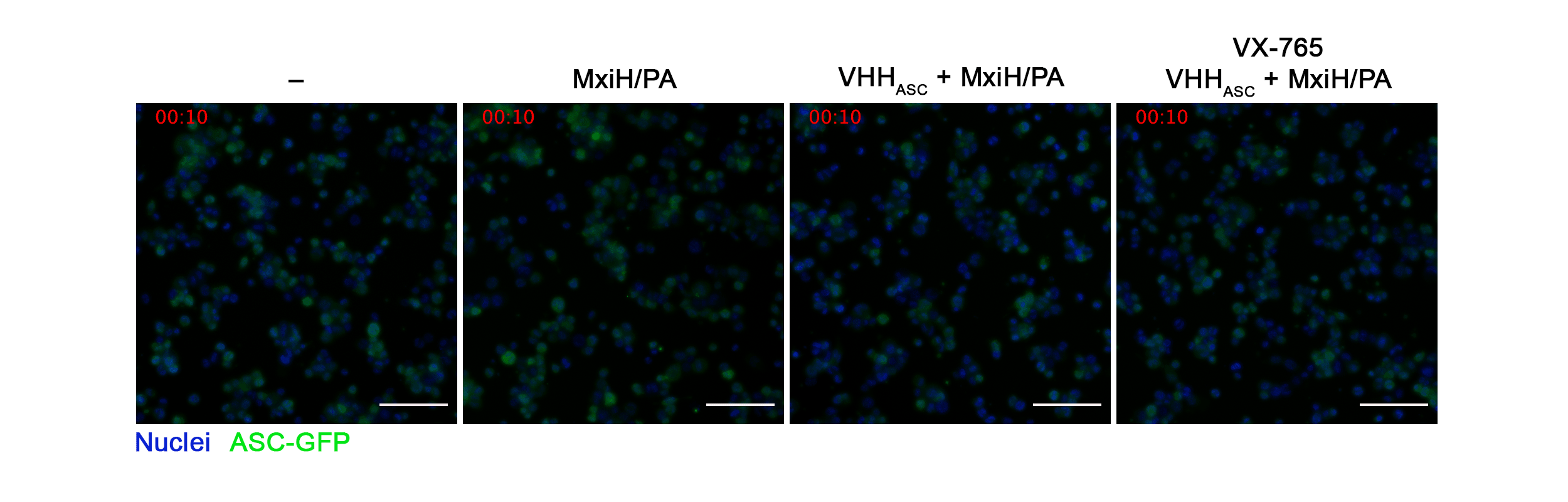

Supplement: Supplementary file 6 — Movie EV4 [file EMMM-14-e15415-s014.zip › Movie EV4.gif]

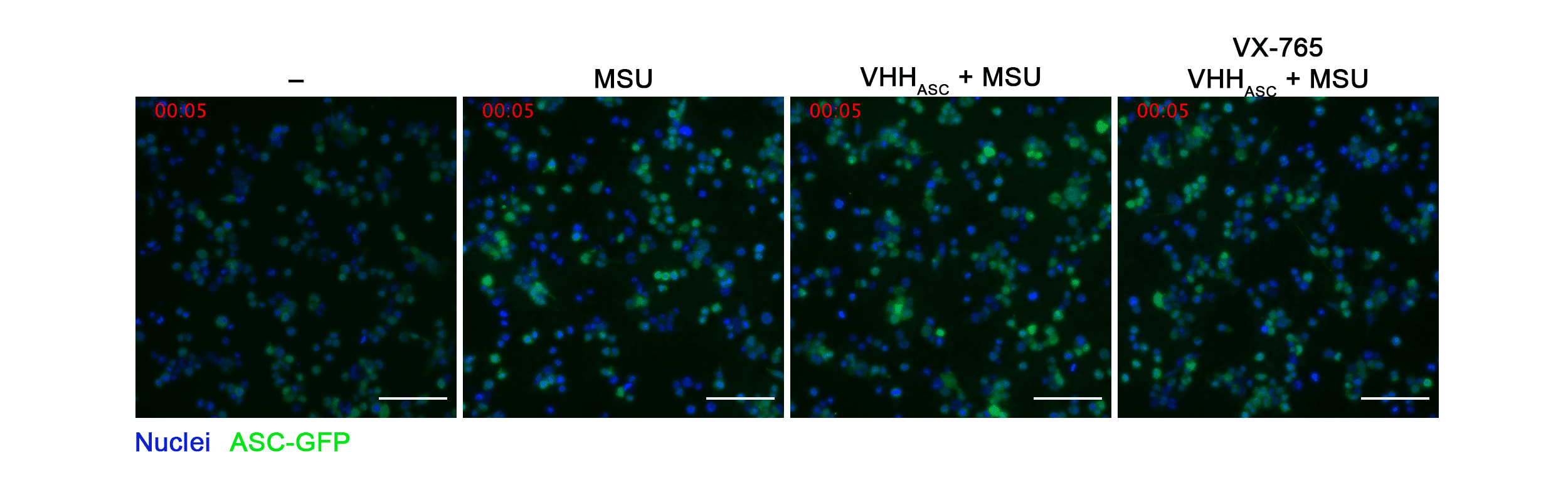

Supplement: Supplementary file 7 — Movie EV5 [file EMMM-14-e15415-s002.zip › Movie EV5.gif]

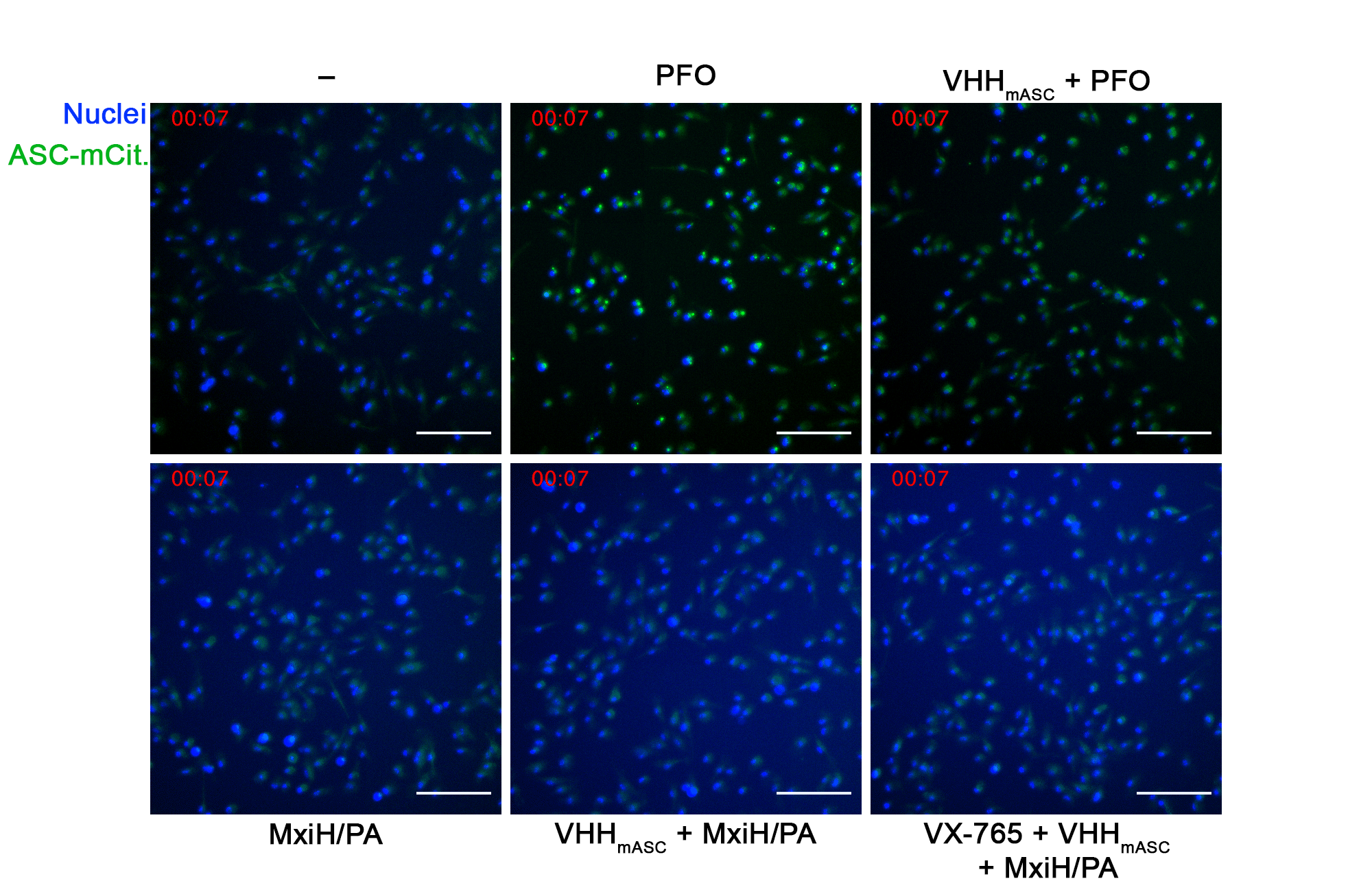

Supplement: Supplementary file 8 — Movie EV6 [file EMMM-14-e15415-s003.zip › Movie EV6.gif]

**Original uncropped Western-blot images - Figure EV5D**

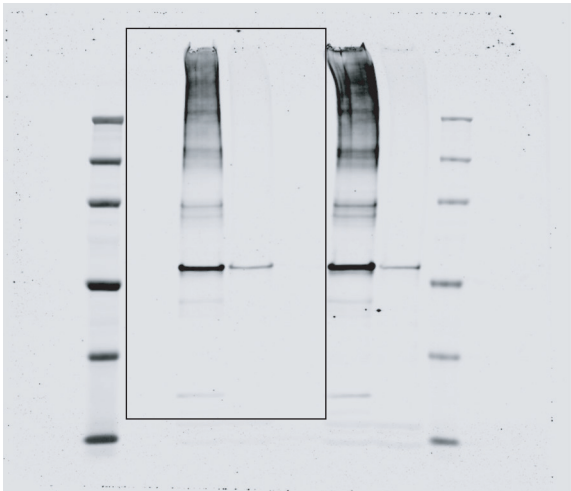

IB: ASC

Supplement: Supplementary file 9 — Source Data for Expanded View [file EMMM-14-e15415-s013.zip › Original_Uncropped_WB_Figure_EV5D.pdf]

**Original uncropped Western-blot images - Figure EV5B**

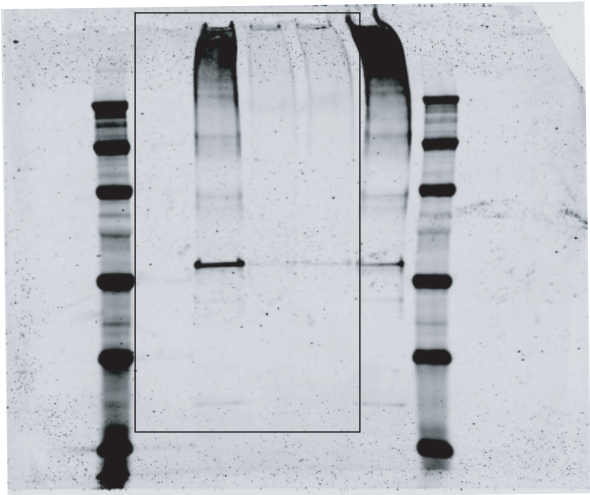

IB: ASC

Supplement: Supplementary file 9 — Source Data for Expanded View [file EMMM-14-e15415-s013.zip › Original_Uncropped_WB_Figure_EV5B.pdf]

**Original uncropped Western-blot images - Figure 1B**

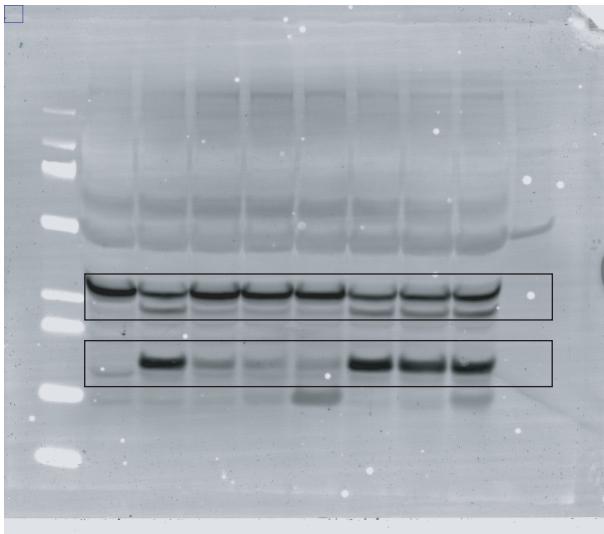

IB: IL-1 $\beta$

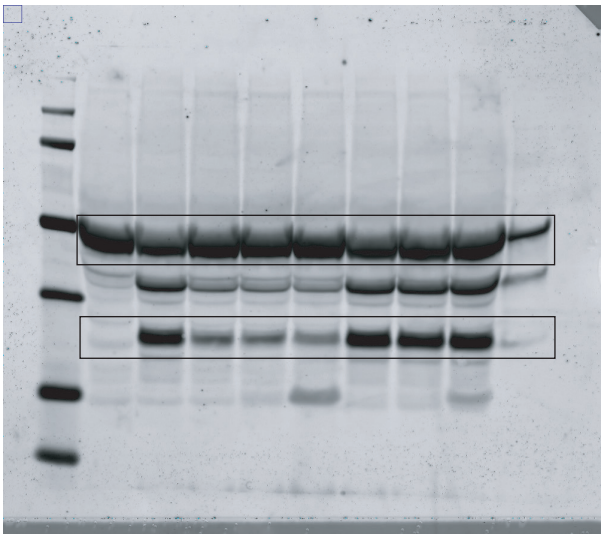

IB: Caspase-1

Supplement: Supplementary file 10 — Source Data for Figure 1 [file EMMM-14-e15415-s005.pdf]

Original uncropped Western-blot images - Figure 2C

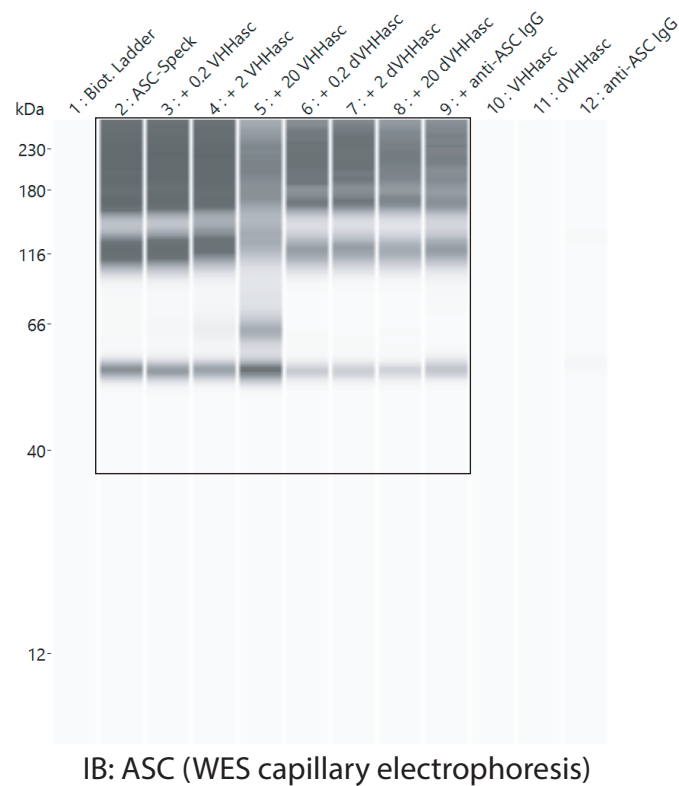

Supplement: Supplementary file 11 — Source Data for Figure 2 [file EMMM-14-e15415-s010.pdf]

Original uncropped Western-blot images - Figure 4A

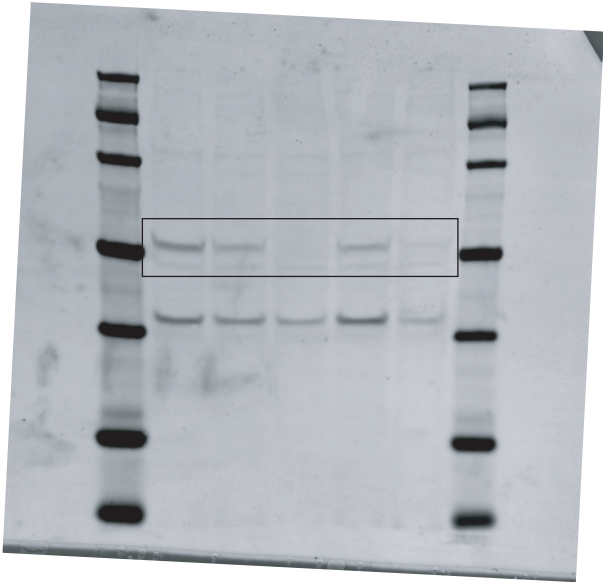

IB: GSDMD

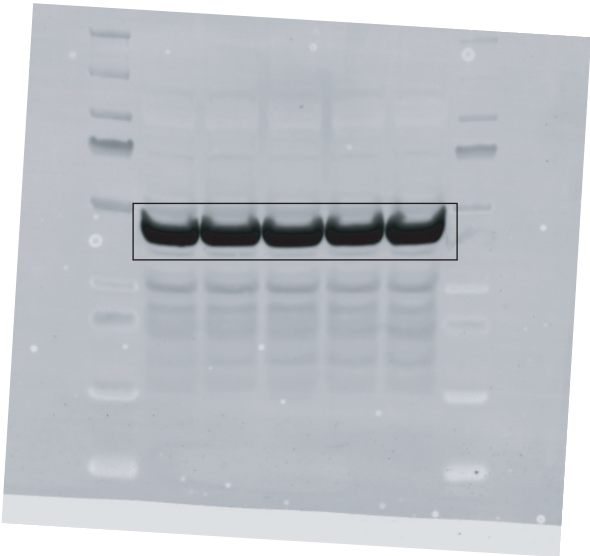

IB:  $\beta$ -Actin

Supplement: Supplementary file 12 — Source Data for Figure 4 [file EMMM-14-e15415-s007.pdf]

**Original uncropped Western-blot images - Figure 5D**

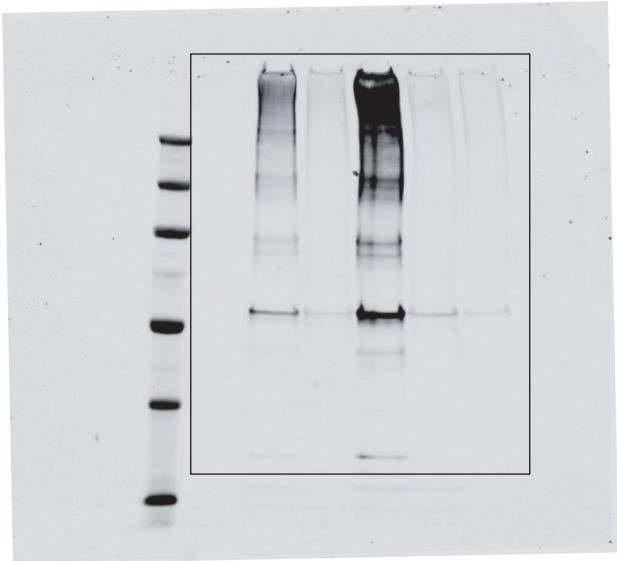

IB: ASC

Supplement: Supplementary file 13 — Source Data for Figure 5 [file EMMM-14-e15415-s011.pdf]
