# Supplementary material for: Nanobodies dismantle post‐pyroptotic ASC specks and counteract inflammation in vivo
Source: EMBO Mol Med. 2022 Apr 19;14(6):e15415. doi: 10.15252/emmm.202115415 (PMC9174887; doi:10.15252/emmm.202115415)
Supplement: Supplementary file 5 — Movie EV3 [file EMMM-14-e15415-s006.zip › Movie EV3_Legend.docx]

**Single-domain antibodies against ASC disassemble post-pyroptotic inflammasomes and reveal their role in inflammatory diseases**

Damien Bertheloot^1^, Carlos W. de Souza Wanderley^2,3^, Ayda Henriques Schneider^2,3^, Lisa Schiffelers^1^, Jennifer D. Wuerth^1^, Jan Tödtmann^4^, Salie Maasewerd^1^, Ibrahim Hawwari^1^, Fraser Duthie^1^, Cornelia Rohland^1^, Lucas S. Ribeiro^1^, Lea Jenster^1^, Nathalia Rosero^1^, Yonas Mehari Tesfamariam^1^, Fernando Q. Cunha^2,3^, Florian I. Schmidt^1, 4^ and Bernardo S. Franklin^1^

**Affiliations:**

^1^Institute of Innate Immunity, Medical Faculty, University of Bonn, 53127 Bonn, NRW, Germany.

^2^Center for Research in Inflammatory Diseases (CRID), Ribeirao Preto Medical School, University of Sao Paulo, Brazil.

^3^Department of Pharmacology, Ribeirao Preto Medical School, University of Sao Paulo, Brazil.

^4^Core Facility Nanobodies, Medical Faculty, University of Bonn, 53127 Bonn, Germany

Correspondence: [d.berthellot@uni-bonn.de](mailto:d.berthellot@uni-bonn.de), [fschmidt@uni-bonn.de](mailto:fschmidt@uni-bonn.de), [franklin@uni-bonn.de](mailto:franklin@uni-bonn.de)

**Running Title:** VHH_ASC_ targets extracellular ASC specks.

**Movie EV3: VHH_ASC_ targets ASC specks in AIM2 inflammasome-activated cells undergoing pyroptosis.**

Nuclei of PMA-differentiated THP-1 macrophages expressing human ASC-GFP (green) were stained using DRAQ5 (blue). VHH_ASC_ (100 µg ml^–1^) or a combination of VHH_ASC_ and VX-765 (VX, 50 µM) were added to the cells followed by 30 min incubation at 37ºC, 5% CO_2_. poly(dA:dT) (dA:dT, 1 µg ml^−1^) was then added 5 min prior to beginning of live imaging on a CellDiscoverer 7 microscope with the inner chamber equilibrated at 37ºC and 5% CO_2_. A total of 8 positions within 2 wells (2x4 images/well) were imaged for 3h. Data are representative kinetic of maximal projection images as gif file and are from one experiment out of three independent experiments. Scale bars represent 100 µm and time stamps represent time after addition of poly(dA:dT) (h:min).
