# Supplementary material for: Nanobodies dismantle post‐pyroptotic ASC specks and counteract inflammation in vivo
Source: EMBO Mol Med. 2022 Apr 19;14(6):e15415. doi: 10.15252/emmm.202115415 (PMC9174887; doi:10.15252/emmm.202115415)
Supplement: Supplementary file 8 — Movie EV6 [file EMMM-14-e15415-s003.zip › Movie EV6_Legend.docx]

**Single-domain antibodies against ASC disassemble post-pyroptotic inflammasomes and reveal their role in inflammatory diseases**

Damien Bertheloot^1^, Carlos W. de Souza Wanderley^2,3^, Ayda Henriques Schneider^2,3^, Lisa Schiffelers^1^, Jennifer D. Wuerth^1^, Jan Tödtmann^4^, Salie Maasewerd^1^, Ibrahim Hawwari^1^, Fraser Duthie^1^, Cornelia Rohland^1^, Lucas S. Ribeiro^1^, Lea Jenster^1^, Nathalia Rosero^1^, Yonas Mehari Tesfamariam^1^, Fernando Q. Cunha^2,3^, Florian I. Schmidt^1, 4^ and Bernardo S. Franklin^1^

**Affiliations:**

^1^Institute of Innate Immunity, Medical Faculty, University of Bonn, 53127 Bonn, NRW, Germany.

^2^Center for Research in Inflammatory Diseases (CRID), Ribeirao Preto Medical School, University of Sao Paulo, Brazil.

^3^Department of Pharmacology, Ribeirao Preto Medical School, University of Sao Paulo, Brazil.

^4^Core Facility Nanobodies, Medical Faculty, University of Bonn, 53127 Bonn, Germany

Correspondence: [d.berthellot@uni-bonn.de](mailto:d.berthellot@uni-bonn.de), [fschmidt@uni-bonn.de](mailto:fschmidt@uni-bonn.de), [franklin@uni-bonn.de](mailto:franklin@uni-bonn.de)

**Running Title:** VHH_ASC_ targets extracellular ASC specks.

**Movie EV6: VHH_mASC_ targets ASC specks in NLRC4 or NLRP3 inflammasome-activated mouse cells.**

Nuclei of mouse bone marrow-derived macrophages expressing human ASC-mCitrin (green) were stained using DRAQ5 (blue). LFn-MxiH/PA (100 ng ml^–1^/1 µg ml^–1^) or PFO (250 ng ml^–1^) were then added 5 min prior to beginning of live imaging on a CellDiscoverer 7 microscope with the inner chamber equilibrated at 37ºC and 5% CO_2_. A total of 8 positions within 2 wells (2x4 images/well) were imaged for 3h. Data are representative kinetic of maximal projection images as gif file and are from one experiment out of three independent experiments. Scale bars represent 100 µm and time stamps represent time after addition of LFn-MxiH/PA or PFO (h:min).
