# Supplementary material for: Nanobodies dismantle post‐pyroptotic ASC specks and counteract inflammation in vivo
Source: EMBO Mol Med. 2022 Apr 19;14(6):e15415. doi: 10.15252/emmm.202115415 (PMC9174887; doi:10.15252/emmm.202115415)
Supplement: Supplementary file 14 [file EMMM-14-e15415-s009.pdf]

EMBO Molecular Medicine

Nanobodies dismantle post-pyroptotic ASC specks and counteract inflammation in vivo

Damien Bertheloot, Carlos Wanderley, Ayda Schneider, Lisa Schiffelers, Jennifer Wuerth, Jan Tödtmann, Salie Maasewerd, Ibrahim Hawwari, Frasier Duthie, Cornelia Rohland, Lucas Ribeiro, Lea Jenster, Nathalia Rosero, Yonas Tesfamariam, Fernando Q Cunha, Florian Schmidt, and Bernardo Franklin
DOI: 10.15252/emmm.202115415

Corresponding authors: Bernardo Franklin (franklin@uni-bonn.de) , Florian Schmidt (fschmidt@uni-bonn.de), Damien Bertheloot (d.bertheloot@uni-bonn.de)

Review Timeline:

Submission Date:	10th Nov 21
Editorial Decision:	2nd Dec 21
Revision Received:	18th Mar 22
Editorial Decision:	28th Mar 22
Revision Received:	29th Mar 22
Accepted:	31st Mar 22

Editor: Zeljko Durdevic

Transaction Report:

(Note: With the exception of the correction of typographical or spelling errors that could be a source of ambiguity, letters and reports are not edited. Depending on transfer agreements, referee reports obtained elsewhere may or may not be included in this compilation. Referee reports are anonymous unless the Referee chooses to sign their reports.)

2nd Dec 2021

Dear Dr. Franklin,

Thank you for the submission of your manuscript to EMBO Molecular Medicine. We have now received feedback from the three reviewers who agreed to evaluate your manuscript. As you will see from the reports below, the referees acknowledge the interest of the study but also raise important critique that should be addressed in a major revision. Particular attention should be given to a better explanation of the camelid-derived single-domain antibodies against ASC (VHHASC) targeting ASC-CARD domain and dissembling ASC specks and to clarifying whether the VHHASC can be used as pan-inflammasome blockers as suggested by the referee #1.

Further consideration of a revision that addresses reviewers' concerns in full will entail a second round of review. EMBO Molecular Medicine encourages a single round of revision only and therefore, acceptance or rejection of the manuscript will depend on the completeness of your responses included in the next, final version of the manuscript. For this reason, and to save you from any frustrations in the end, I would strongly advise against returning an incomplete revision.

We would welcome the submission of a revised version within three months for further consideration. Please let us know if you require longer to complete the revision.

Please use this link to login to the manuscript system and submit your revision: https://embomolmed.msubmit.net/cgi-bin/main.plex

I look forward to receiving your revised manuscript.

Yours sincerely,

Zeljko Durdevic

Zeljko Durdevic
Editor
EMBO Molecular Medicine

*****

***** Reviewer's comments *****

Referee #1 (Remarks for Author):

The MS submitted by Bertheloot et al. demonstrate the satisfactory use of a nanobody anti-ASC able to impair inflammasome signaling by dissembling ASC oligomers or specks. This nanobody is effective in impairing NLRP3 inflammasome activation when is intracellularly delivered and is also able to dissemble already formed ASC specks (either extracellularly or intracellularly when the nanobody enters the cell during pyroptosis). Then the nanobody was tested in two in vivo models of gout and antigen-induced arthritis. The therapeutical use of nanobodies is increasing over other small drug-like molecules due to their ability to easily combine with other nanobodies, it relatively short live in the organism, its low impact activating the immune system as they lack Fc domains and above all their target specificity. This study is quite exciting as is the first demonstration of the feasibility to specifically target ASC therapeutically for deleterious effects of the aberrant inflammasome activation, however several issues should be clarified to improve the broad impact of the study:

1. The use of nanobodies against ASC would be a pan-inflammasome blocker as it could potentially target several inflammasome sensors that use ASC to signal. This is stated in the title and abstract where there is no explicit mention to a specific inflammasome. However the authors mainly focus the results on the NLRP3 inflammasome. They show a single experiment demonstrating that the nanobody has not effect on NLRC4 activation (Fig S2E), but in these conditions it neither blocked NLRP3 signaling (Fig 3A) as it could not enter into targeted cells, being these assays performed measuring released IL-1b. The authors then show that the nanobody anti-ASC decrease the number of cells with ASC-specks after NLRP3 activation with nigericin or PFO (Fig. 4A,B). The authors should also assess the effect of the anti-ASC VHH on ASC-specking induced with other NLRP3-activators as MSU crystals (mimicking and additionally supporting the gouty animal model used) or imiquimod, as well as after other inflammasome activation: AIM2, Pyrin, NLRP1 and NLRC4, measuring released IL-1b and intracellular ASC-specking. These experiments will give the answer if this anti-ASC nanobody could be considered as a pan-inflammasome inhibitor.

2. The authors reported in 2014 that pyroptosis also induce release of ASC specks from the cells. In Fig 4b, the decrease of intracellular ASC-specks could be also due to an increase of the release of ASC specks from the cells. Could the authors demonstrate that the presence of the nanobody also decreases the release of ASC-specks? This could be done not only for NLRP3 activation, but also for other inflammasomes as is suggested in the previous point.

3. The authors already published in 2016 that the VHH anti-human ASC resulted in the formation of large ASC filaments due to PYD-PYD interactions, since this nanobody bind to the CARD domain and blocked CARD-CARD interactions important for the formation of the ASC speck by the ASC filaments. In the Fig 2A, one would expect that the nanobody would result in ASC-mTurquoise filaments from ASC-GFP seed specks. Could the authors explain why this is not the case and implement this into the discussion. This is a critical point that should be explained.

4. The novel VHH anti-mouse ASC developed in this study binds to the PYD domain of ASC and therefore targets a different domain when compared to the anti-human ASC nanobody. Could the authors show that this nanobody has no binding affinity to the PYD of NLRP3 or the PYD of other inflammasome sensors? As in Fig 3E,F the effect of this nanobody has been assessed using NLRP3 activation with PFO and would be interesting to know that this is not due to the nanobody binding to NLRP3 PYD and blocking the initial seed of ASC. What is the effect of this nanobody in a CARD-driving inflammasome, as the NAIP/NLRC4 inflammasome? Would be also able to block its signaling?

5. Regarding internalization of the nanobody to the cells, the authors show indirect data suggesting that in fact the internalization

of the nanobody is required to block intracellular ASC-specks. However, this has not been directly shown, and the authors should show intracellular detection of the nanobody with the different cellular activation used (nigericin, PFO, nigericin+VX765, PFO+VX765), i.e. by specific staining, FACS or other biochemical analysis of cellular fractions. Would not be possible to deliver the nanobody intracellularly, similarly that when flagellin is delivered into the cells for NAIP/NLRC4 activation, and check that once intracellularly, the nanobody could block ASC speck formation from the starting and IL-1b release will be prevented.

6. Regarding the animal model of gout, since in vitro it has been shown that the nanobody could target extracellular ASC specks and dissemble them and this is a late pyroptotic-related event, would not be better to treat gout once the flare has been established? The right control in this model would require to i.p. administrate either a mutant VHH-ASC or an irrelevant VHH to compare and not PBS. It might be that the presence of the nanobody (independently of its target) could have some effect in the establishment of the gouty model. Similarly, this model has been shown quite specific of NLRP3 by using NLRP3-KO mice, but would be interesting to compare the effect of the nanobody against ASC with the ASC-KO mice.

7. Similarly, in the antigen-induced arthritis model, the right control would be a non-sense VHH. In this model authors should explain why blocking IL-1 with IL-1RA has a similar output than blocking extracellular ASC, since the authors show in the in vitro data that with the presence of the nanobody anti-ASC IL-1b is still released from the cells and even one of the conclusions of the study is that this therapy could target ASC, but not IL-1. While IL-1 is important for host defense, clinical data using anakinra and other IL-1 targeting therapies demonstrate that IL-1 blocking is not inducing immunosuppression of the patients and have a clear therapeutic benefit. This should be discussed and conclusion of the study modified accordingly, as the authors show that their nanobodies present a similar effect than IL-1RA.

Minors:

1. Figure 1B: the purified ASC specks seems to come from macrophage lysates and therefore present some pro-caspase-1 as well as some p20 caspase-1. My suggestion is that this experiment would be more clean by using recombinant ASC-specks produced in cells lacking caspase-1, as in HEK293 cells or similar. What was the reason not to use a ASC speck without pro-caspase-1?

2. Figure 3A-D: The legend of the y axe of the graphs state hIL-1b or mIL-1b, to differentiate human and mouse IL-1b, while this acronym is not explained in the figure legend, I recommend to state in the top of the graph human MDMs or mouse BMDMs and in the axe state just IL-1b. Of note, mIL-1b could be confused with "mature" IL-1b after caspase-1 processing of the pro-IL-1b.

3. Some experimental panels show data from N=2 replicates, from these is difficult to conclude robust effects since statistic cannot be performed. I would suggest to increase at least to N=3 all the experimental replicates to gain statistical power.

4. Full uncropped Western blots from all panel figures of the paper should be shown in supplementary figures.

5. Figure 1A: The ratio of ASC-specks/cell (not only the concentration) used should be stated in the figure legend.

6. It is not clear for both animal models how leukocytes have been assessed from the synovial fluid of healthy (not swelling) joints. In healthy conditions there is almost any synovial fluid to collect, could the authors clarify the methods employed for this in the MS.

7. Authors state in the abstract and introduction (lines 44-46) that accumulation of ASC specks occurs in tissues of patients. Some references of primary data showing this should be added.

8. The authors should describe well the term "inflammasome" in the introduction (i.e. in line 9), are they referring to the ASC-speck, or just the oligomerization of the sensor inflammasome protein, or the sensor+ASC+caspase-1 into a large multiprotein complex?

9. When discussing the effect of the nanobody in the gouty model, the authors should mention that early clinical trials with a NLRP3 inhibitor (Dapansutrile) show efficacy in the reduction of joint pain in gout flares (PMID: 33005902).

10. The first paragraph of the discussion stating the different investments of companies in the field of NLRP3 inhibitors is out of the scope and just a general sentence stating the pharma interest in the development of inflammasome blocking drugs would be sufficient.

11. Figure S1A and C are not in order related to their first appearance in the text, please fix. The results of the different panels of Fig S1 should be better explained in the text, not just state the final conclusion of the experiment as they represent different assays to the one shown in the main figures and the different conditions of the graphs used in the panels of Fig S1 are not easy to interpret.

12. Please adjust alignment of the x-axe labels of Figure S2, in some panels the symbols are moved respect to the bars.

Referee #2 (Comments on Novelty/Model System for Author):

The study is based on relevant and well-established pre-clinical (murine) models of gouty inflammation and antigen-induced inflammatory arthritis. These in vivo mouse studies are complemented by mechanistic in vitro experiments with primary human macrophages and human myeloid cell lines to establish that the camelid anti-ASC antibodies (VHH-Asc) can suppress ASC-driven inflammasome signaling in living cells and by disassembling extracellular ASC complexes released from pyroptotic cells.

Referee #2 (Remarks for Author):

General Comments: This is a well-designed and appropriately interpreted study that describes the ability of camelid-derived anti-ASC antibodies (VHH-Asc) to suppress ASC-driven inflammasome signaling in living cells and by disassembling extracellular ASC complexes released from pyroptotic cells. The potential clinical/therapeutic relevance is supported by the use of well-established pre-clinical (murine) models of gouty inflammation and antigen-induced inflammatory arthritis. These in vivo mouse studies are complemented by mechanistic in vitro experiments that establish the suppressive effects of the VHH-ASC on: 1) inflammasome signaling in primary human macrophages; 2) ASC-speck accumulation in human myeloid leukocyte cell lines and murine bone marrow-derived macrophages expressing fluorescence-tagged ASC constructs; and 3) biochemical/ biophysical analyses of recombinant ASC assembling into prionoid-like complexes. Overall, the data support the ability of the VHH-ASC reagents to limit ASC inflammasome-dependent inflammatory pathology by predominantly attenuating the positive "feed-forward" amplification triggered by pyroptotic release of ASC complexes into extracellular compartments, phagocytotic uptake of those complexes by naive bystander (or recruited) myeloid leukocytes, and consequent "seeding" of inflammasome activation in the latter leukocytes. Importantly, this attenuation of the feed-forward amplification cascade occurs downstream of, or after, an initial burst of mature IL-1b release from the initial compartment of inflammasome-active cells. This minimizes the potential risk of increased microbial infection due to loss of host-protective early phase IL-1b production, as occurs with therapeutics that directly target IL-1b or initial upstream inflammasome activation, while targeting the later phases of amplified IL-1b production that contribute to chronic host-debilitating inflammation. Only a few technical or mechanistic issues require clarification or extension.

Specific Comments:

1. (Minor) In the legend for Figs 5B/5C, the authors note that the clinical assessments were performed at 3- and 6-hours post-challenge. While these two time points are shown in panel B (mechanical pain threshold), data from only a single point in shown in panel C (edema). I assume the latter edema measurements were taken at 6 hrs, but this needs to be specified in the figure legend. The same issue applies to the data and figure legend for Figs S5A and B.

2.Fig S2C: the authors should comment on why VX-765 effectively rescues viability of BMDM challenged with BsaK/PA (Panel F- to stimulate NLRC4 inflammasomes) but not nigericin (Panel C- to stimulate NLRP3 inflammasomes).

3. Fig 4A/B: These data suggest that early phase NLRP3 inflammasome activation (by nigericin) induces GSDMD pores which facilitate influx and cytosolic accumulation of VHH-ASC in amounts sufficient to disassemble ASC complexes and thereby inhibit/reverse sustained inflammasome signaling and (presumed) production/assembly of plasma membrane GSDMD pores. This raises two intriguing and significant questions that should be addressed by a few experiments: a) can GSDMD pores indeed facilitate direct influx of extracellular VHH-ASC? b) are these cells that have accumulated cytosolic VHH-ASC (after early-phase plasma membrane GSDMD assembly) viable and protected against on-going pyroptotic progression?

Referee #3 (Comments on Novelty/Model System for Author):

I selected medium for medical impact as this is proof-of-concept study using preclinical models, however the concept has a translational potential once it is decided which conditions to target.

Referee #3 (Remarks for Author):

The study by Bertheloot et al. describes that single-domain antibodies targeting human or mouse ASC are able to disassemble preformed ASC specks when specks are not protected by intact plasma membrane. They first demonstrate that previously characterized nanobody VHHASC targeting human ASC-CARD domain but not its nonbinding variant inhibits seeded ASC polymerization, caspase-1 processing and IL-1b in vitro. As VHHASC binds poorly to mouse ASC the authors further developed VHHmASC that binds to PYD domain of mouse ASC. Further they demonstrate that nanobodies cannot enter intact cells thus mostly act on extracellular ASC specks or in cells which were severely porated (with perfringolysin O). In cells undergoing pyroptosis, VHHmASC disassembles specks, yet no decrease of IL-1b is observed. The highlight of the paper is that VHHmASC decreased inflammation and improved clinical parameters in vivo mouse models of gout and arthritis.
This is a well-designed study and I find it exciting for several reasons. It demonstrates that at least secreted ASC specks can be disassembled, suggesting that targeting other complexes where protein conformation is retained. Amelioration of disease in in

vivo models provides a proof-of -concept for usage of nanobodies in treating inflammation and suggests that extracellular ASC specks might have a prominent role in inducing inflammation in chronic diseases. Drugs that stop inflammasomes at the stage of ASC and dissembling those particles might be prove useful in many chronic diseases.

I have some minor comments:

1. I find it surprising that VHHASC that targets CARD-CARD domain interactions is so efficient in disrupting ASC specks in vitro, particularly since it has been shown that this nanobody blocks crosslinking of ASCPYD filaments when expressed in the cytosol of cells. So I would expect that ASC specks would become less compact but not totally disassembled. On the other hand anti-ASC polyclonals are not effective at all in the same setup-why? I would expect that affinity is comparable-is it the size or simply that polyclonals do not target segments that engage in interacting surfaces. The authors demonstrated that VHHmASC binds PYD domain. Do they have any indication which segment is targeted? They cite several previous studies that showed the presence of extracellular ASC specks and the presence of anti-ASC antibodies in chronic patients that were not protective but rather enhanced inflammation by Fc-mediated ASC particle uptake. It is likely that antibody responses to ASC aggregates are raised against surface epitopes, and thus the key to presented nanobodies' success is mostly due to targeting the right epitopes with high affinity.
2. Have the authors considered more traditional approaches for increasing efficiency of plasma membrane or BBB transfer such as fusion of nanobodies with cell penetrating peptides?
3. How have the authors decided on the amount of VHH used in in vivo models?
4. Typos: page 3, line 27-reference is in superscript; page 5, line 113: verbs should be in plural or singular; page 20: lines 564 and 565- are ER2738 and ER2837 really two different bacterial strains or a typo?, figure 5 caption, line 4 synovial

Point-by-point response to the Reviewer's comments:

Dear Dr. Durdevic, Dear Reviewers,

Firstly, we would like to thank the editor and the editorial board to consider our manuscript entitled "Single-domain antibodies against ASC disassemble post-pyroptotic inflammasomes and reveal their role in inflammatory diseases". We would also like to thank the reviewers for their time and efforts judging our manuscript.
In the following pages, we provide a point-by-point response to the Reviewer's comments. We are confident that the extensive new data sets and specific answers address the points raised by the reviewers and have overall improved the manuscript substantially.

Reviewer 1:
The MS submitted by Bertheloot et al. demonstrate the satisfactory use of a nanobody anti-ASC able to impair inflammasome signaling by dissembling ASC oligomers or specks. This nanobody is effective in impairing NLRP3 inflammasome activation when is intracellularly delivered and is also able to dissemble already formed ASC specks (either extracellularly or intracellularly when the nanobody enters the cell during pyroptosis). Then the nanobody was tested in two in vivo models of gout and antigen-induced arthritis. The therapeutical use of nanobodies is increasing over other small drug-like molecules due to their ability to easily combine with other nanobodies, it relatively short live in the organism, its low impact activating the immune system as they lack Fc domains and above all their target specificity. This study is quite exciting as is the first demonstration of the feasibility to specifically target ASC therapeutically for deleterious effects of the aberrant inflammasome activation, however several issues should be clarified to improve the broad impact of the study:

Response: We thank this Reviewer for the kind words about our work and for highlighting its potential implications. We did our best to fully address all the points raised by this reviewer. We are also grateful for his/her comprehensive assessment of our manuscript and are confident that the new datasets will improve our manuscript's quality.

Question 1: The use of nanobodies against ASC would be a pan-inflammasome blocker as it could potentially target several inflammasome sensors that use ASC to signal. This is stated in the title and abstract where there is no explicit mention to a specific inflammasome. However, the authors mainly focus the results on the NLRP3 inflammasome. They show a single experiment demonstrating that the nanobody has no effect on NLRC4 activation (Fig S2E), but in these conditions it neither blocked NLRP3 signaling (Fig 3A) as it could not enter into targeted cells, being these assays performed measuring released IL-1b. The authors then show that the nanobody anti-ASC decrease the number of cells with ASC-specks after NLRP3 activation with nigericin or PFO (Fig. 4A,B). The authors should also assess the effect of the anti-ASC VHH on ASC-specking induced with other NLRP3-activators as MSU crystals (mimicking and additionally supporting the gouty animal model used) or imiquimod, as well as after other inflammasome activation: AIM2, Pyrin, NLRP1 and NLRC4, measuring released IL-1b and intracellular ASC-specking. These experiments will give the answer if this anti-ASC nanobody could be considered as a pan-inflammasome inhibitor.

Response: The reviewer raises a very important point, as it is true, and relevant, that by targeting ASC, anti-ASC VHHs (VHH_{ASC}) could have potential application against the activation of other ASC-dependent inflammasomes.

Our study already included data showing that VHH_{ASC} does not prevent IL-1β release from cells activated with the NLRP3 activator Nigericin (**Fig. 3A, C**), nor with the NLRC4 activator LFn-BsaK/PA (now **Figure EV2E** and **Figure EV5C-D** of the revised manuscript). We demonstrated that VHH_{ASC} requires early access to the cytosol to target ASC and prevent IL-1β release. This was only achieved when we used the bacterial toxin PFO, which has been shown to form large pores in the plasma membrane (~40 nm) and to trigger the NLRP3-dependent release of IL-1β (Yamamura et al., 2019). Our aim in this study was to target extracellular and/or post-pyroptotic ASC specks, as targeting of intracellular ASC by endogenous expression of VHH_{ASC} has been sufficiently described (Schmidt et al., 2016).

As requested by this Reviewer, we have now included further data showing the effect of VHH_{ASC} on the release of IL-1β, and in the viability of cells stimulated with ATP or MSU crystals (NLRP3, new **Figures EV2C** and **EV2D** of the revised manuscript), TcdB (Pyrin, new **Figure EV2F**), Val-boroPro (NLRP1**, Figure EV2G**) and poly(dA:dT) (AIM2, **Figure EV2H**). In these experiments, VHH_{ASC} did not inhibit release of IL-1β induced by the different stimuli, indicating that it does not interfere with the early IL-1-driven host immune responses before it can enter pyroptotic cells.

However, we have also included new data demonstrating that VHH_{ASC} can disrupt post-pyroptotic ASC specks after GSDMD pore formation activated by various triggers: THP-1 cells expressing ASC-GFP were stimulated with poly(dA:dT) (AIM2) and LFn-MxiH/PA (NLRC4) (new Figures EV5A-B and EV5C-D of the revised manuscript). We reproducibly observed that ASC specks disappeared over time when GSDMD pores were formed. We also analyzed the activity of VHH_{ASC} against MSU-induced ASC specks by live imaging (Figure EV5E) and surprisingly found that specks formation was suppressed even in the absence of caspase-1 activity. THP-1 cells do not express NLRP1 (rather CARD8) and do not respond to TcdB via Pyrin but via NLRP3 (CRID3 sensitive). We believe that the newly generated data with the activation of AIM2 and NLRC4 will suffice as evidence that VHH_{ASC} is able to target all ASC-dependent inflammasomes following pyroptosis.

Question 2: The authors reported in 2014 that pyroptosis also induce release of ASC specks from the cells. In Fig 4b, the decrease of intracellular ASC-specks could be also due to an increase of the release of ASC specks from the cells. Could the authors demonstrate that the presence of the nanobody also decreases the release of ASC-specks? This could be done not only for NLRP3 activation, but also for other inflammasomes as is suggested in the previous point.

Response: In the experiments presented in **Figure 4C** of our original manuscript, we demonstrated the oligomeric state of ASC specks released from inflammasome activated cells into the culture supernatants (sups). We showed that the treatment of extracellular ASC specks with VHH_{ASC} led to their destabilization. However, the conditions used were somewhat different to those used in the microscopy experiment (Figure 4A-B). We have now replaced the previous Figure 4C with a set of new data, in which we applied flow cytometry to measure the number of specks released into the culture supernatants by inflammasome-activated cells (**Figure 5C** and **Appendix Figure S1A-B** in the revised manuscript). We also analyzed the oligomeric structure of ASC released into the culture supernatants using DSS cross-linking and western-blotting (new **Figure 5D** in the revised manuscript). As recommended by this reviewer, we also investigated ASC specks released by THP-1 ASC-GFP cells stimulated with poly(dA:dT) (AIM2, **Figure EV5A-B**) or LFn-MxiH/PA (NLRC4, **Figure EV5C-D**). In all experiments, we found that stimulation of inflammasomes induced the release of ASC specks into the supernatants. Consistent with our previous observation on the NLRP3 inflammasome, our new data also shows that VHH_{ASC} decreased the accumulation of ASC specks measured directly in the culture supernatants as assessed by flow cytometry and Western blot. Together, these new datasets support

our previous conclusion and provide conclusive evidence that VHH_{ASC} targets post-pyroptotic ASC specks formed upon activation of multiple inflammasome sensors.

Question 3: The authors already published in 2016 that the VHH anti-human ASC resulted in the formation of large ASC filaments due to PYD-PYD interactions, since this nanobody bind to the CARD domain and blocked CARD-CARD interactions important for the formation of the ASC speck by the ASC filaments. In the Fig 2A, one would expect that the nanobody would result in ASC-mTurquoise filaments from ASC-GFP seed specks. Could the authors explain why this is not the case and implement this into the discussion. This is a critical point that should be explained.

Response: Firstly, we would like to point out that in the study mentioned by the reviewer (Schmidt et al.,2016), VHH_{ASC} was over-expressed in the cytosol of cells (THP-1 or A549) prior to inflammasome activation. In these conditions, the early presence of VHH_{ASC} in the cytosol blocked CARD-CARD interactions, while still allowing the formation of PYD-PYD-only interactions resulting in PYD filaments. In **Figure 2A**, and throughout the experimental settings in our manuscript, VHH_{ASC} targeted ASC specks that were already naturally assembled in inflammasome-activated pyroptotic cells. We expect that PYD-filaments released after the disassembly of ASC specks induced by VHH_{ASC}, would diffuse away in these cell-free settings and would be impossible to detect with the conditions used to image the very bright ASC specks. While it is likely possible to establish the conditions to see PYD filaments, we do not think this would substantially add to the content of the manuscript and would thus be outside the scope of our study.

Question 4: The novel VHH anti-mouse ASC developed in this study binds to the PYD domain of ASC and therefore targets a different domain when compared to the anti-human ASC nanobody. Could the authors show that this nanobody has no binding affinity to the PYD of NLRP3 or the PYD of other inflammasome sensors? As in Fig 3E,F the effect of this nanobody has been assessed using NLRP3 activation with PFO and would be interesting to know that this is not due to the nanobody binding to NLRP3 PYD and blocking the initial seed of ASC. What is the effect of this nanobody in a CARD-driving inflammasome, as the NAIP/NLRC4 inflammasome? Would be also able to block its signaling?

Response: Nanobodies are generated for their high specificity against their targets. Although the amino acid sequences of ASC^{PYD} and $NLRP3^{PYD}$ have only 25.3% similarity, we have now added additional evidence for the specificity of VHH_{mASC} to mouse ASC^{PYD} in the stringent LUMIER assay (new **Figure EV3C** in the revised manuscript): We compared the binding of VHH_{mASC} to mouse ASC^{PYD}, human ASC^{PYD} and mouse $NLRP3^{PYD}$. As expected, VHH_{mASC} exclusively displayed binding affinity to mouse ASC^{PYD}. Furthermore, we have tested the inhibitory activity of VHH_{mASC} against ASC specks in mouse BMDMs expressing ASC-mCitrine. We stimulated these cells with LFn-MxiH/PA to activate NLRC4 and induced the formation of ASC specks initiated by $NLRC4^{CARD}$:ASC^{CARD} interactions (new **Figure 5E** and **Appendix Movie S6** in the revised manuscript). As observed with VHH_{ASC} (**Figure EV5C**), specks formed upon NLRC4 activation were disassembled over time in the presence of VHH_{mASC}, reaching a ratio of speck-containing cells similar to non-stimulated cells. Together, these data confirm the specificity of VHH_{mASC} for ASC^{PYD} and demonstrate that VHH_{mASC} is able to target ASC specks formed upon activation of another sensor than NLRP3.

Question 5: Regarding internalization of the nanobody to the cells, the authors show indirect data suggesting that in fact the internalization of the nanobody is required to block intracellular ASC-specks. However, this has not been directly shown, and the authors should show intracellular detection of the nanobody with the different cellular activation used (nigericin, PFO, nigericin+VX765, PFO+VX765), i.e. by specific staining, FACS or other biochemical analysis of cellular fractions. Would not be possible to deliver the nanobody intracellularly, similarly that when flagellin is delivered into the cells for

NAIP/NLRC4 activation, and check that once intracellularly, the nanobody could block ASC speck formation from the starting and IL-1b release will be prevented.

Response: Access of the VHHs to the cytosol after pore formation is supported by the activity we observed against ASC specks in pyroptotic cells. Furthermore, we would like to emphasize that we have previously demonstrated that ASC specks become accessible to extracellular fluorescently-labeled anti-ASC antibody in cells undergoing pyroptosis (Franklin et al., 2014), (https://static-content.springer.com/esm/art%3A10.1038%2Fni.2913/MediaObjects/41590_2014_BFni2913_MOESM6_ESM.mov). As VHHs are significantly smaller than antibodies, it is highly likely that they access intracellular ASC through membrane pores, as supported by our findings in PFO-stimulated cells. We now specifically addressed this point by imaging the entry and co-localization of fluorescently-labeled VHH_{ASC} (VHH_{ASC}-AF647) to endogenous ASC specks formed by inflammasome activation in ASC-GFP expressing THP-1 cells (new **Figure 4C** in the revised manuscript). Furthermore, we show that caspase-1 inhibition prevents the co-localization of the VHH_{ASC}-AF647 to GFP-specks.

This reviewer also raises an interesting point, which is the requirement for membrane pores for intracellular access of the anti-ASC VHHs. To directly show that, we have now added additional data demonstrating the entry of VHH_{ASC} into the cells upon inflammasome-activation in cells where GSDMD is conditionally knocked out by doxycycline (Dox) treatment (new **Figure EV4** and **Appendix Movie S1** in the revised manuscript). We demonstrate that fluorescently-labeled VHH_{ASC} (VHH_{ASC}-AF647) directly applied into the culture supernatants entered inflammasome-activated GSDMD-expressing cells (-Dox) 30 minutes after stimulation with Nigericin. In GSDMD-KO cells (+Dox), VHH_{ASC}-AF647 remained outside the cells. To confirm loss of membrane viability we additionally applied propidium iodide (PI) into the culture supernatants. In contrast, upon stimulation with PFO, VHH_{ASC}-AF647 enters the cells more rapidly (from 5 min) and independently of GSDMD expression. In this case, entry of VHH_{ASC}-AF647 even preceded PI staining.

Regarding the alternative mechanisms to deliver VHHs into the cytosol: we have focused in our manuscript on the activity of exogenous administered VHH_{ASC} to specifically target post-pyroptotic inflammasomes (extracellular or following GSDMD activation and pyroptosis). This is a scenario more likely to recapitulate clinical settings. The effect of VHH_{ASC} on ASC-dependent inflammasomes inside cells has been previously established (Schmidt et al 2016). Hence, testing other artificial methods of delivery of nanobodies across the plasma membranes would be out of the scope of this manuscript.

Question 6: Regarding the animal model of gout, since in vitro it has been shown that the nanobody could target extracellular ASC specks and dissemble them and this is a late pyroptotic-related event, would not be better to treat gout once the flare has been established? The right control in this model would require to i.p. administrate either a mutant VHH-ASC or an irrelevant VHH to compare and not PBS. It might be that the presence of the nanobody (independently of its target) could have some effect in the establishment of the gouty model. Similarly, this model has been shown quite specific of NLRP3 by using NLRP3-KO mice, but would be interesting to compare the effect of the nanobody against ASC with the ASC-KO mice.

Response: This is indeed a very important, and clinically relevant point. The reviewer is correct that using a delayed treatment with VHH_{mASC} would indeed reflect better clinical settings. We have therefore repeated the gout model, this time allowing the MSU-induced inflammation and gout disease phenotype to start before treating with VHH_{mASC} (3 h post-MSU i.a. injection, New **Figure 6** of the revised manuscript). As suggested by the reviewer, we have also included an unrelated VHH control (VHH NP-1) to further strengthen our data. Strikingly, treatment with VHH_{mASC} ameliorated clinical parameters of disease (higher pain threshold and decreased joint swelling) and induced a decrease in leukocyte infiltration and tissue cytokines. The control VHH had no such effects, further demonstrating the specificity of the activity of VHH_{mASC}. Regarding the use of ASC KO mice, we would like to respectfully state that the gout model we have used is well described and specific to NLRP3. The dependency of

NLRP3 to ASC is evidently well established (Amaral et al., 2012; Martinon et al., 2006). The use of ASC KO mice in our experiment would only serve to demonstrate that MSU-induced gout is inflammasome-dependent. We respectfully argue that this experiment is out of scope of our study, and it would not justify the sacrifice of animals (following the 3R principle).

Question 7: Similarly, in the antigen-induced arthritis model, the right control would be a non-sense VHH. In this model authors should explain why blocking IL-1 with IL-1RA has a similar output than blocking extracellular ASC, since the authors show in the in vitro data that with the presence of the nanobody anti-ASC IL-1b is still released from the cells and even one of the conclusions of the study is that this therapy could target ASC, but not IL-1. While IL-1 is important for host defense, clinical data using anakinra and other IL-1 targeting therapies demonstrate that IL-1 blocking is not inducing immunosuppression of the patients and have a clear therapeutic benefit. This should be discussed and conclusion of the study modified accordingly, as the authors show that their nanobodies present a similar effect than IL-1RA.

Response: Although we agree with the reviewer that the AIA model could benefit from an unrelated VHH control, we hope the new in vivo data in the MSU model, where we have used the control suggested by the reviewer, will suffice to show the specificity of VHH_{mASC} in vivo. We do not feel this control would ethically justify for the sacrifice of many animals to repeat the entire study for the AIA model as well.
Anakinra is one of the treatments used in the clinic to treat arthritis and has been well studied in this mouse model. We used Anakinra to compare the efficacy of VHH_{mASC} with that of a benchmark treatment, and not to suggest that the mechanisms involved with the inhibitory activities of VHH_{mASC} and anakinra are the same. Anakinra targets IL-1R and therefore blocks inflammatory signals and neutrophil recruitment induced by IL-1β. In our previous study (Franklin et al, 2014), we showed that the presence of extracellular specks induces the release of IL-1β from phagocytic cells as an amplification loop that extends the pro-inflammatory potential of inflammasomes. In the present study, we show that the intracellular activation of inflammasomes is not inhibited by VHH_{mASC}. However, we also show that VHH_{mASC} targets post-pyroptotic specks. Hence, treatment with VHH_{mASC} should prevent the amplification loop induced by post-pyroptotic inflammasomes and thus induce a decreased IL-1β as well as a decreased infiltration of pro-inflammatory cells, as demonstrated in Figure 7D-E. We have now added text to even better reflect this in the discussion.

Minors Comments:
Minor Comment 1: Figure 1B: the purified ASC specks seems to come from macrophage lysates and therefore present some pro-caspase-1 as well as some p20 caspase-1. My suggestion is that this experiment would be more clean by using recombinant ASC-specks produced in cells lacking caspase-1, as in HEK293 cells or similar. What was the reason not to use a ASC speck without pro-caspase-1?

Response: ASC specks from myeloid cells are well-established assays that were used by us (Franklin et al., 2014) and others (Baroja-Mazo et al., 2014; de Almeida et al., 2015; Fernandes-Alnemri et al., 2007). We employed this method as expression and isolation of recombinant full-length ASC from HEK cells is difficult, as ASC can spontaneously aggregate and thus differ from inflammasome-nucleated ASC specks. Furthermore, we believe that macrophage-derived ASC specks more closely resemble physiological conditions where pyroptotic cells release their ASC specks into the extracellular environment. In fact, the above mentioned studies showed that extracellular ASC specks contain caspase-1 and other inflammasome components. In our data, the control used – ASC specks in the absence of pro-caspase-1- and pro-IL-1β-containing lysates (lane 9) – clearly shows that the preparations of ASC specks used only contained a minute amount of caspase-1 when compared to the levels found in lysates alone (lane 1). The ASC speck preparation also did not contain any IL-1β, reproducing previously published data (Baroja-Mazo et al., 2014; Fernandes-Alnemri et al., 2007;

Franklin et al., 2014). Finally, previous studies have demonstrated that caspase-1 is not active for very long after it is autoproteolytically cleaved (Boucher *et al*, 2018).

Minor Comment 2: Figure 3A-D: The legend of the y axe of the graphs state hIL-1b or mIL-1b, to differentiate human and mouse IL-1b, while this acronym is not explained in the figure legend, I recommend to state in the top of the graph human MDMs or mouse BMDMs and in the axe state just IL-1b. Of note, mIL-1b could be confused with "mature" IL-1b after caspase-1 processing of the pro-IL-1b.

Response: We thank the reviewer for his/her input. We have now better described the meaning of hIL-1β or mIL-1β in the figure legends.

Minor Comment 3: Some experimental panels show data from N=2 replicates, from these is difficult to conclude robust effects since statistic cannot be performed. I would suggest to increase at least to N=3 all the experimental replicates to gain statistical power.

Response: We appreciate this reviewer concern for the robustness of the data on our manuscript. We would like to point out that, each dataset on our figures is combined from different biological replicates (performed with different donors, or batch of cell lines) each containing at least two technical replicates. Furthermore, we mostly restricted to two biological replicates for experiments that support well-established conclusions previously achieved by other publications. For example, our original **Figure 1A** showed that ASC specks induce the release of IL-1β from stimulated mouse macrophages, an observation repeatedly described (Baroja-Mazo et al., 2014; de Almeida et al., 2015; Franklin et al., 2014; Venegas et al., 2017). We apologize to this reviewer if this was not better explained in our figure legends/text. While we stand by our data and are confident that it sustains the conclusion made in this manuscript, we have now repeated experiments, whenever feasible.

Minor Comment 4: Full uncropped Western blots from all panel figures of the paper should be shown in supplementary figures.

Response: We are fully aware of this requirement, and we firmly stand behind good scientific practice and reproducibility in science. We have all original and uncropped western-blots and have included them in **Appendix Original uncropped Western-Blots.**

Minor Comment 5: ratio of ASC-specks/cell (not only the concentration) used should be stated in the figure legend.

Response: Assessing ASC specks protein concentration is a well-accepted and more reliable way to estimate the immunogenicity of ASC specks. This is because preparations of ASC specks are usually heterogeneous as these complexes aggregate with many cytosolic proteins (Sahillioglu and Ozoren, 2015). The protein concentration, rather than ratio of specks/cell, was established so one could directly assess the immune activity of ASC specks and compare it to similar amounts of “mock speck preparations” from ASC-deficient cells (Franklin et al., 2014).

Minor Comment 6: It is not clear for both animal models how leukocytes have been assessed from the synovial fluid of healthy (not swelling) joints. In healthy conditions there is almost any synovial fluid to collect, could the authors clarify the methods employed for this in the MS.

Response: We apologize for missing to clearly describe the method used to quantify leukocytes from the joint synovial space. This has now been added to the Material and Methods section. Briefly, we perform tissue lavage by injecting joints with 2.5 μL of PBS, 4 times/joint) and collecting the lavages into

50 µL of PBS in one well of a 96 well plate. Cells are pellet and resuspended in 50 µL staining buffer with antibodies specific to the described cell markers, and finally used for Flow Cytometry.

Minor Comment 7: Authors state in the abstract and introduction (lines 44-46) that accumulation of ASC specks occurs in tissues of patients. Some references of primary data showing this should be added.

Response: We would like to respectfully refer the Reviewer to lines 46-52, where we give many examples and references of the accumulation of ASC specks in patient tissues. In brief, extracellular ASC specks are found in the circulation of patients with CAPS (Baroja-Mazo et al., 2014), HIV (Ahmad et al., 2018), cancer (Basiorka et al., 2018), in lavages of tissues of patients with COPD (Franklin et al., 2014), and in spinal cord fluid (de Rivero Vaccari et al., 2008), or brain lysates during neuroinflammation (Venegas et al., 2017).

Minor Comment 8: The authors should describe well the term "inflammasome" in the introduction (i.e. in line 9), are they referring to the ASC-speck, or just the oligomerization of the sensor inflammasome protein, or the sensor+ASC+caspase-1 into a large multiprotein complex?

Response: We thank the reviewer for his/her input. Unfortunately, the literature is not consistent on this subject. We always refer to inflammasomes as the assembly of a sensor with ASC and recruited caspase-1, and have now stated this more clearly in the manuscript.

Minor Comment 9: When discussing the effect of the nanobody in the gouty model, the authors should mention that early clinical trials with a NLRP3 inhibitor (Dapansutrile) show efficacy in the reduction of joint pain in gout flares (PMID: 33005902).

Response: We thank the reviewer for this suggestion. The reference has been added to the text.

Minor Comment 10: The first paragraph of the discussion stating the different investments of companies in the field of NLRP3 inhibitors is out of the scope and just a general sentence stating the pharma interest in the development of inflammasome blocking drugs would be sufficient.

Response: We thank the reviewer for her/his input. This paragraph has been modified accordingly, and it is highlighted in the text.

Minor Comment 11: Figure S1A and C are not in order related to their first appearance in the text, please fix. The results of the different panels of Fig S1 should be better explained in the text, not just state the final conclusion of the experiment as they represent different assays to the one shown in the main figures and the different conditions of the graphs used in the panels of Fig S1 are not easy to interpret.

Response: We thank the reviewer for pointing this out. We have re-ordered the figure panels and provided additional explanation of the assays and their interpretation to the text.

Minor Comment 12: Please adjust alignment of the x-axe labels of Figure S2, in some panels the symbols are moved respect to the bars.

Response: We thank the reviewer for pointing this issue out and we apologize for missing this mistake prior to submission. We have now re-aligned the labels and symbols.

Reviewer 2:
The study is based on relevant and well-established pre-clinical (murine) models of gouty inflammation and antigen-induced inflammatory arthritis. These in vivo mouse studies are complemented by mechanistic in vitro experiments with primary human macrophages and human myeloid cell lines to establish that the camelid anti-ASC antibodies (VHH-Asc) can suppress ASC-driven inflammasome signaling in living cells and by disassembling extracellular ASC complexes released from pyroptotic cells.

General Comments: This is a well-designed and appropriately interpreted study that describes the ability of camelid-derived anti-ASC antibodies (VHH-Asc) to suppress ASC-driven inflammasome signaling in living cells and by disassembling extracellular ASC complexes released from pyroptotic cells. The potential clinical/therapeutic relevance is supported by the use of well-established pre-clinical (murine) models of gouty inflammation and antigen-induced inflammatory arthritis. These in vivo mouse studies are complemented by mechanistic in vitro experiments that establish the suppressive effects of the VHH-ASC on: 1) inflammasome signaling in primary human macrophages; 2) ASC-speck accumulation in human myeloid leukocyte cell lines and murine bone marrow-derived macrophages expressing fluorescence-tagged ASC constructs; and 3) biochemical/ biophysical analyses of recombinant ASC assembling into prionoid-like complexes. Overall, the data support the ability of the VHH-ASC reagents to limit ASC inflammasome-dependent inflammatory pathology by predominantly attenuating the positive "feed-forward" amplification triggered by pyroptotic release of ASC complexes into extracellular compartments, phagocytotic uptake of those complexes by naive bystander (or recruited) myeloid leukocytes, and consequent "seeding" of inflammasome activation in the latter leukocytes. Importantly, this attenuation of the feed-forward amplification cascade occurs downstream of, or after, an initial burst of mature IL-1b release from the initial compartment of inflammasome-active cells. This minimizes the potential risk of increased microbial infection due to loss of host-protective early phase IL-1b production, as occurs with therapeutics that directly target IL-1b or initial upstream inflammasome activation, while targeting the later phases of amplified IL-1b production that contribute to chronic host-debilitating inflammation. Only a few technical or mechanistic issues require clarification or extension.

Response: We thank the reviewer for her/his time and for the positive assessment of our work. We respond to this reviewer's points below.

Specific Comment 1: (Minor) In the legend for Figs 5B/5C, the authors note that the clinical assessments were performed at 3- and 6-hours post-challenge. While these two time points are shown in panel B (mechanical pain threshold), data from only a single point in shown in panel C (edema). I assume the latter edema measurements were taken at 6 hrs, but this needs to be specified in the figure legend. The same issue applies to the data and figure legend for Figs S5A and B.

Response: We apologize for missing to precisely specify the time point for measurement of the edema. The reviewer is correct in her/his assuming that the measurement was done at 6h. This detail has now been added to the legend.

Specific Comment 2: Fig S2C: the authors should comment on why VX-765 effectively rescues viability of BMDM challenged with BsaK/PA (Panel F- to stimulate NLRC4 inflammasomes) but not nigericin (Panel C- to stimulate NLRP3 inflammasomes).

Response: We thank Reviewer 2 for her/his comment. Nigericin and PFO are very toxic chemicals as they disturb the ion gradient across the plasma membrane. They are notoriously potent inflammasome

activators, but can also affect cell viability in an inflammasome-independent manner, especially after prolonged exposure (incubation times). Hence, given enough time nigericin will induce cell death even in the absence of NLRP3. We would like to mention that for our experiments, we have rather used CTB as readout of cell viability as it more closely monitors the cell health and metabolic activity. Hence, CTB will not only reflect on the cell pyroptosis but also on the general health of the cells treated with the different activators of inflammasomes. Furthermore, in our hands, we have often seen that CRID3 is a better inhibitor then VX-765 with lower off-target cell toxicity (please also see the shift between CRID3 and VX-765 in Figure EV2A, in human macrophages treated with nigericin). With increasing concentration of VX-765, cell toxicity effects can be observed. In this case, it is possible that the added toxicity of Nigericin and VX-765 have prevented the full rescue of cell viability that should be expected by blocking caspase-1 activity. We find treatment with LFn-BsaK/PA is not as toxic to cells in the absence of inflammasome activity. There, VX-765 was therefore able to rescue cell death induced by activation of caspase-1 downstream of NLRC4.

Specific Comment 3: Fig 4A/B: These data suggest that early phase NLRP3 inflammasome activation (by nigericin) induces GSDMD pores which facilitate influx and cytosolic accumulation of VHH-ASC in amounts sufficient to disassemble ASC complexes and thereby inhibit/reverse sustained inflammasome signaling and (presumed) production/assembly of plasma membrane GSDMD pores. This raises two intriguing and significant questions that should be addressed by a few experiments: a) can GSDMD pores indeed facilitate direct influx of extracellular VHH-ASC? b) are these cells that have accumulated cytosolic VHH-ASC (after early-phase plasma membrane GSDMD assembly) viable and protected against on-going pyroptotic progression?

Response: In the new Figure EV4, we used GSDMD-KO cells to demonstrate that GSDMD is required for entry of VHH_{ASC} into pyroptotic cells following stimulation with nigericin (**Figure EV4A**, in the revised manuscript). In the case of PFO stimulation, GSDMD expression has no impact on the internalization of VHH_{ASC}. This new data, together with the large number of supporting evidence for the requirement of pyroptosis or membrane pores to allow entry of VHH_{ASC} into the cytosol, further solidifies our hypothesis that GSDMD pores enable VHH_{ASC} to target post-pyroptotic specks but does not prevent full IL-1β release.

For the viability of the cells treated with VHH_{ASC}, our data (**Figure 3A-D**, **Figure EV2** and **EV3D-F**) show that upon stimulation of different inflammasome sensors, the presence of VHH_{ASC} or VHH_{mASC} neither prevents the maximal release of IL-1β nor rescues the cell viability. This demonstrates that VHH_{ASC} cannot protect against ongoing pyroptosis as it does not gain access to the cytosol early enough. This would indeed be counterintuitive, as pyroptosis in the first place is a requirement for entry of VHH_{ASC} into cells.

Reviewer 3:

I selected medium for medical impact as this is proof-of-concept study using preclinical models, however the concept has a translational potential once it is decided which conditions to target.

The study by Bertheloot et al. describes that single-domain antibodies targeting human or mouse ASC are able to disassemble preformed ASC specks when specks are not protected by intact plasma membrane. They first demonstrate that previously characterized nanobody VHHASC targeting human ASC-CARD domain but not its nonbinding variant inhibits seeded ASC polymerization, caspase-1 processing and IL-1b in vitro. As VHHASC binds poorly to mouse ASC the authors further developed VHHmASC that binds to PYD domain of mouse ASC. Further they demonstrate that nanobodies cannot enter intact cells thus mostly act on extracellular ASC specks or in cells which were severely porated

(with perfringolysin O). In cells undergoing pyroptosis, VHHmASC disassembles specks, yet no decrease of IL-1b is observed. The highlight of the paper is that VHHmASC decreased inflammation and improved clinical parameters in vivo mouse models of gout and arthritis.
This is a well-designed study and I find it exciting for several reasons. It demonstrates that at least secreted ASC specks can be disassembled, suggesting that targeting other complexes where protein conformation is retained. Amelioration of disease in in vivo models provides a proof-of -concept for usage of nanobodies in treating inflammation and suggests that extracellular ASC specks might have a prominent role in inducing inflammation in chronic diseases. Drugs that stop inflammasomes at the stage of ASC and dissembling those particles might be prove useful in many chronic diseases.

Response: We thank the reviewer for her/his time and for her/his positive assessment of our work. We respond to this reviewer's points below.

I have some minor comments:

Comment 1: I find it surprising that VHHASC that targets CARD-CARD domain interactions is so efficient in disrupting ASC specks in vitro, particularly since it has been shown that this nanobody blocks crosslinking of ASCPYD filaments when expressed in the cytosol of cells. So I would expect that ASC specks would become less compact but not totally disassembled. On the other hand anti-ASC polyclonals are not effective at all in the same setup-why? I would expect that affinity is comparable-is it the size or simply that polyclonals do not target segments that engage in interacting surfaces. The authors demonstrated that VHHmASC binds PYD domain. Do they have any indication which segment is targeted? They cite several previous studies that showed the presence of extracellular ASC specks and the presence of anti-ASC antibodies in chronic patients that were not protective but rather enhanced inflammation by Fc-mediated ASC particle uptake. It is likely that antibody responses to ASC aggregates are raised against surface epitopes, and thus the key to presented nanobodies' success is mostly due to targeting the right epitopes with high affinity.

Response: In our previous study (Schmidt et al 2016), the over-expression of VHH_{ASC} prior to inflammasome activation enabled the formation of filaments assembled uniquely through PYD-PYD interactions. In this context, only PYDs are available for interaction, enabling the stable formation of a filament over time. In Figure 2, VHH_{ASC} was added to already formed specks which are assembled by both PYD-PYD and CARD-CARD interactions. In these settings, VHH_{ASC} targets CARD-CARD interactions disassembling the speck. Outside cells, as was the case for these in vitro experiments, ASC would most likely diffuse away limiting the formation of large PYD-based filaments. Hence, PYD-based filaments would most likely not be visible at the applied resolution, and their fluorescence would bleach quickly under the required microscopy settings. In our imaging of live cells (Figure 5A), VHH_{ASC} can only access ASC specks inside pyroptotic cells or following the formation of large pores (PFO) in the plasma membrane. In these circumstances, the opened cytosolic space will again limit the accumulation of ASC to a concentration necessary for formation of large filaments. In either case, the scope of our manuscript is to demonstrate that VHH_{ASC} disassemble active ASC specks and inhibit their pro-inflammatory and prionoid activities. We believe that all together, our *in vitro* and *in vivo* data supports the idea of VHH_{ASC} and VHH_{mASC} as potent inhibitors of post-pyroptotic inflammasomes.

The reason why conventional antibodies do not disassemble specks is because they most probably cannot reach their target epitope within the speck. Nanobodies have been developed for a combination of two important properties: their high affinity and their small size. These properties enable nanobodies to penetrate within nooks and crannies of folded proteins or protein complexes. We believe this gives VHH_{ASC} and VHH_{mASC} a strong advantage for the targeting and destabilization of ASC specks. We do not have more precise data of which segment of the ASC^{PYD} is targeted by VHH_{mASC}. Such information would require a structure or hydrogen deuterium exchange (HDX) studies which would be beyond the scope of this manuscript.

Nanobodies do not have an Fc domain. In our previous study (Franklin et al 2014), we showed that targeting extracellular ASC specks with conventional antibodies (which possess an Fc domain) increased the pro-inflammatory potential of the specks because they induced the Fc-mediated opsonization of the specks by phagocytic cells. Hence, the fact that VHH_{ASC} or VHH_{mASC} do not possess an Fc-domain might be yet one more factor making them successful at targeting post-pyroptotic inflammasomes in vitro and in vivo.

Comment 2: Have the authors considered more traditional approaches for increasing efficiency of plasma membrane or BBB transfer such as fusion of nanobodies with cell penetrating peptides?

Response: We have tested the use of peptide-mediated delivery of nanobodies and found it to work very poorly. Furthermore, we believe that the fact that VHH_{ASC} does not pass the plasma membrane is beneficial as this provides specificity for post-pyroptotic inflammasomes either present in the extracellular space or inside pyroptotic cells. Thus, to develop membrane-permeable VHH_{ASC} would unfortunately be out of the scope of this study.
For the delivery of VHH_{ASC} to the CNS, we are indeed considering different methods that would enable VHH_{ASC} to pass the BBB (as described in the Discussion section). However, much work will be required to test these systems and to make sure VHH_{ASC} preserves its activity. We respectfully believe this would best be published in a separate article.

Comment 2: How have the authors decided on the amount of VHH used in in vivo models?

Response: We have selected the dose of 5mg/kg based on our long-standing experience with nanobodies and a preliminary study where we had used a lower dose (2mg/kg) which had shown only a partial effect with systemic exposure. This dose however had shown strong activity of VHH_{ASC} as local delivery (**Appendix Figure S4A-C**).

Comment 3: Typos: page 3, line 27-reference is in superscript; page 5, line 113: verbs should be in plural or singular; page 20: lines 564 and 565- are ER2738 and ER2837 really two different bacterial strains or a typo?, figure 5 caption, line 4 synovial

Response: We apologize for these mistakes and thank Reviewer 3 for taking the time to bring them to our attention. These have now been corrected.

References cited in this letter:

Ahmad, F., Mishra, N., Ahrenstorf, G., Franklin, B.S., Latz, E., Schmidt, R.E., and Bossaller, L. (2018). Evidence of inflammasome activation and formation of monocyte-derived ASC specks in HIV-1 positive patients. AIDS *32*, 299-307. 10.1097/QAD.0000000000001693.

Amaral, F.A., Costa, V.V., Tavares, L.D., Sachs, D., Coelho, F.M., Fagundes, C.T., Soriani, F.M., Silveira, T.N., Cunha, L.D., Zamboni, D.S., et al. (2012). NLRP3 inflammasome-mediated neutrophil recruitment and hypernociception depend on leukotriene B(4) in a murine model of gout. Arthritis Rheum *64*, 474-484. 10.1002/art.33355.

Baroja-Mazo, A., Martín-Sánchez, F., Gomez, A.I., Martínez, C.M., Amores-Iniesta, J., Compan, V., Barberà-Cremades, M., Yagüe, J., Ruiz-Ortiz, E., Antón, J., et al. (2014). The NLRP3 inflammasome is released as a particulate danger signal that amplifies the inflammatory response. Nature Publishing Group *15*, 738-748. 10.1038/ni.2919.

Basiorka, A.A., McGraw, K.L., Abbas-Aghababazadeh, F., McLemore, A.F., Vincelette, N.D., Ward, G.A., Eksioglu, E.A., Sallman, D.A., Ali, N.A., Padron, E., et al. (2018). Assessment of ASC specks as a putative biomarker of pyroptosis in myelodysplastic syndromes: an observational cohort study. Lancet Haematol *5*, e393-e402. 10.1016/S2352-3026(18)30109-1.

Boucher D, Monteleone M, Coll RC, Chen KW, Ross CM, Teo JL, Gomez GA, Holley CL, Bierschenk D, Stacey KJ et al (2018) Caspase-1 self-cleavage is an intrinsic mechanism to terminate inflammasome activity. The Journal of experimental medicine: jem.20172222-20172220

de Almeida, L., Khare, S., Misharin, A.V., Patel, R., Ratsimandresy, R.A., Wallin, M.C., Perlman, H., Greaves, D.R., Hoffman, H.M., Dorfleutner, A., and Stehlik, C. (2015). The PYRIN Domain-only Protein POP1 Inhibits Inflammasome Assembly and Ameliorates Inflammatory Disease. Immunity *43*, 264-276. 10.1016/j.immuni.2015.07.018.

de Rivero Vaccari, J.P., Lotocki, G., Marcillo, A.E., Dietrich, W.D., and Keane, R.W. (2008). A Molecular Platform in Neurons Regulates Inflammation after Spinal Cord Injury. Journal of Neuroscience *28*, 3404-3414. 10.1523/JNEUROSCI.0157-08.2008.

Fernandes-Alnemri, T., Wu, J., Yu, J.-W., Datta, P., Miller, B., Jankowski, W., Rosenberg, S., Zhang, J., and Alnemri, E.S. (2007). The pyroptosome: a supramolecular assembly of ASC dimers mediating inflammatory cell death via caspase-1 activation. Cell Death and Differentiation *14*, 1590-1604. 10.1038/sj.cdd.4402194.

Franklin, B.S., Bossaller, L., De Nardo, D., Ratter, J.M., Stutz, A., Engels, G., Brenker, C., Nordhoff, M., Mirandola, S.R., Al-Amoudi, A., et al. (2014). The adaptor ASC has extracellular and 'prionoid' activities that propagate inflammation. Nat Immunol *15*, 727-737. 10.1038/ni.2913.

Martinon, F., Pétrilli, V., Mayor, A., Tardivel, A., and Tschopp, J. (2006). Gout-associated uric acid crystals activate the NALP3 inflammasome. Nature *440*, 237-241. 10.1038/nature04516.

Sahillioglu, A.C., and Ozoren, N. (2015). Artificial Loading of ASC Specks with Cytosolic Antigens. PLoS ONE *10*, e0134912. 10.1371/journal.pone.0134912.

Schmidt, F.I., Lu, A., Chen, J.W., Ruan, J., Tang, C., Wu, H., and Ploegh, H.L. (2016). A single domain antibody fragment that recognizes the adaptor ASC defines the role of ASC domains in inflammasome assembly. The Journal of Experimental Medicine *213*, 771-790. 10.1084/jem.20151790.

Venegas, C., Kumar, S., Franklin, B.S., Dierkes, T., Brinkschulte, R., Tejera, D., Vieira-Saecker, A., Schwartz, S., Santarelli, F., Kummer, M.P., et al. (2017). Microglia-derived ASC specks cross-seed amyloid-β in Alzheimer's disease. Nature *552*, 355-361. 10.1038/nature25158.

Yamamura, K., Ashida, H., Okano, T., Kinoshita-Daitoku, R., Suzuki, S., Ohtani, K., Hamagaki, M., Ikeda, T., and Suzuki, T. (2019). Inflammasome Activation Induced by Perfringolysin O of Clostridium perfringens and Its Involvement in the Progression of Gas Gangrene. Front Microbiol *10*, 2406. 10.3389/fmicb.2019.02406.

28th Mar 2022

Dear Prof. Franklin,

Thank you for the submission of your revised manuscript to EMBO Molecular Medicine. I am pleased to inform you that we will be able to accept your manuscript pending the following final amendments:

1) We note that you currently have together with you, a total of 3 co-corresponding authors. Is that correct? Do you confirm equal contribution of these 3 people, able to take full responsibility for the paper and its content? While there is no limit per se to the number of co-corresponding authors, 3 is rare and may not reflect as intended to the community.
2) In the main manuscript file, please do the following:
- Remove text highlight colour.
- In M&M, provide the antibody dilutions that were used for each antibody.
- In M&M, add statistical paragraph that should reflect all information that you have filled in the Authors Checklist, especially regarding randomization, blinding, replication.
3) Funding: Please add only funding relevant to this study in our submission system as well as in the acknowledgments.
4) Synopsis:
- Please check your synopsis text and image and submit their final versions with your revised manuscript. Please be aware that in the proof stage minor corrections only are allowed (e.g., typos).
5) For more information: Please remove corresponding author's e-mail address. This space should be used to list relevant web links for further consultation by our readers. Could you identify some relevant ones and provide such information as well? Some examples are patient associations, relevant databases, OMIM/proteins/genes links, author's websites, etc...
6) As part of the EMBO Publications transparent editorial process initiative (see our Editorial at http://embomolmed.embopress.org/content/2/9/329), EMBO Molecular Medicine will publish online a Review Process File (RPF) to accompany accepted manuscripts. This file will be published in conjunction with your paper and will include the anonymous referee reports, your point-by-point response and all pertinent correspondence relating to the manuscript. Let us know whether you agree with the publication of the RPF and as here, if you want to remove or not any figures from it prior to publication. Please note that the Authors checklist will be published at the end of the RPF.
7) Please provide a point-by-point letter INCLUDING my comments as well as the reviewer's reports and your detailed responses (as Word file).

I look forward to reading a new revised version of your manuscript as soon as possible.

Yours sincerely,

Zeljko Durdevic

Zeljko Durdevic
Editor
EMBO Molecular Medicine

***** Reviewer's comments *****

Referee #1 (Remarks for Author):

The authors has addressed all the issues raised, the study has been greatly improved with additional experiments.

The authors performed the requested editorial changes.

We are pleased to inform you that your manuscript is accepted for publication and is now being sent to our publisher to be included in the next available issue of EMBO Molecular Medicine.

EMBO Press Author Checklist

Corresponding Author Name: Bernardo S. Franklin	
Journal Submitted to: EMBO Molecular Medicine	
Manuscript Number: EMM-2021-15415	

USEFUL LINKS FOR COMPLETING THIS FORM
The EMBO Journal - Author Guidelines
EMBO Reports - Author Guidelines
Molecular Systems Biology - Author Guidelines
EMBO Molecular Medicine - Author Guidelines

Reporting Checklist for Life Science Articles (updated January 2022)
This checklist is adapted from Materials Design Analysis Reporting (MDAR) Checklist for Authors. MDAR establishes a minimum set of requirements in transparent reporting in the life sciences (see Statement of Task: 10.31222/osf.io/9sm4x). Please follow the journal's guidelines in preparing your manuscript.
Please note that a copy of this checklist will be published alongside your article.

Abridged guidelines for figures
1. Data
The data shown in figures should satisfy the following conditions:
- the data were obtained and processed according to the field's best practice and are presented to reflect the results of the experiments in an accurate and unbiased manner.
- ideally, figure panels should include only measurements that are directly comparable to each other and obtained with the same assay.
- plots include clearly labeled error bars for independent experiments and sample sizes. Unless justified, error bars should not be shown for technical replicates.
- if n<5, the individual data points from each experiment should be plotted. Any statistical test employed should be justified.
- Source Data should be included to report the data underlying figures according to the guidelines set out in the authorship guidelines on Data Presentation.

2. Captions
Each figure caption should contain the following information, for each panel where they are relevant:
- a specification of the experimental system investigated (eg cell line, species name).
- the assay(s) and method(s) used to carry out the reported observations and measurements.
- an explicit mention of the biological and chemical entity(ies) that are being measured.
- an explicit mention of the biological and chemical entity(ies) that are altered/varied/perturbed in a controlled manner.
- the exact sample size (n) for each experimental group/condition, given as a number, not a range;
- a description of the sample collection allowing the reader to understand whether the samples represent technical or biological replicates (including how many animals, litters, cultures, etc.).
- a statement of how many times the experiment shown was independently replicated in the laboratory.
- definitions of statistical methods and measures:
  - common tests, such as t-test (please specify whether paired vs. unpaired), simple χ2 tests, Wilcoxon and Mann-Whitney tests, can be unambiguously identified by name only, but more complex techniques should be described in the methods section;
  - are tests one-sided or two-sided?
  - are there adjustments for multiple comparisons?
  - exact statistical test results, e.g., P values = x but not P values < x;
  - definition of 'center values' as median or average;
  - definition of error bars as s.d. or s.e.m.

Please complete ALL of the questions below.
Select "Not Applicable" only when the requested information is not relevant for your study.

Materials

Newly Created Materials	Information included in the manuscript?	In which section is the information available? (Reagents and Tools Table, Materials and Methods, Figures, Data Availability Section)
New materials and reagents need to be available; do any restrictions apply?	Yes	Materials and Methods and Supplementary Materials

Antibodies	Information included in the manuscript?	In which section is the information available? (Reagents and Tools Table, Materials and Methods, Figures, Data Availability Section)
For **antibodies** provide the following information:<br>- Commercial antibodies: RRID (if possible) or supplier name, catalogue number and or/clone number<br>- Non-commercial: RRID or citation	Yes	Materials and Methods

DNA and RNA sequences	Information included in the manuscript?	In which section is the information available? (Reagents and Tools Table, Materials and Methods, Figures, Data Availability Section)
**Short novel DNA or RNA including primers, probes**: provide the sequences.	Not Applicable	

Cell materials	Information included in the manuscript?	In which section is the information available? (Reagents and Tools Table, Materials and Methods, Figures, Data Availability Section)
**Cell lines:** Provide species information, strain. Provide accession number in repository **OR** supplier name, catalog number, clone number, and/**OR** RRID.	Yes	Materials and Methods
**Primary cultures:** Provide species, strain, sex of origin, genetic modification status.	Not Applicable	
Report if the cell lines were recently **authenticated** (e.g., by STR profiling) and tested for mycoplasma contamination.	Yes	Materials and Methods

Experimental animals	Information included in the manuscript?	In which section is the information available? (Reagents and Tools Table, Materials and Methods, Figures, Data Availability Section)
**Laboratory animals or Model organisms:** Provide species, strain, sex, age, genetic modification status. Provide accession number in repository **OR** supplier name, catalog number, clone number, **OR** RRID.	Yes	Materials and Methods and Supplementary Materials
**Animal observed in or captured from the field:** Provide species, sex, and age where possible.	Not Applicable	
Please detail **housing and husbandry conditions**.	Yes	Materials and Methods and Supplementary Materials

Plants and microbes	Information included in the manuscript?	In which section is the information available? (Reagents and Tools Table, Materials and Methods, Figures, Data Availability Section)
**Plants:** provide species and strain, ecotype and cultivar where relevant, unique accession number if available, and source (including location for collected wild specimens).	Not Applicable	
**Microbes:** provide species and strain, unique accession number if available, and source.	Yes	Supplementary Materials

Human research participants	Information included in the manuscript?	In which section is the information available? (Reagents and Tools Table, Materials and Methods, Figures, Data Availability Section)
If collected and within the bounds of privacy constraints report on age, sex and gender or ethnicity for all study participants.	Not Applicable	

Core facilities	Information included in the manuscript?	In which section is the information available? (Reagents and Tools Table, Materials and Methods, Figures, Data Availability Section)
If your work benefited from core facilities, was their service mentioned in the acknowledgments section?	Yes	Acknowledgments

Design

Study protocol	Information included in the manuscript?	In which section is the information available? (Reagents and Tools Table, Materials and Methods, Figures, Data Availability Section)
If study protocol has been **pre-registered, provide DOI in the manuscript**. For clinical trials, provide the trial registration number **OR** cite DOI.	Not Applicable	
Report the **clinical trial registration number** (at ClinicalTrials.gov or equivalent), where applicable.	Not Applicable	

Laboratory protocol	Information included in the manuscript?	In which section is the information available? (Reagents and Tools Table, Materials and Methods, Figures, Data Availability Section)
Provide DOI OR other citation details if **external detailed step-by-step protocols** are available.	Yes	Main Text and Material and Methods

Experimental study design and statistics	Information included in the manuscript?	In which section is the information available? (Reagents and Tools Table, Materials and Methods, Figures, Data Availability Section)
Include a statement about **sample size** estimate even if no statistical methods were used.	Yes	Materials and Methods
Were any steps taken to minimize the effects of subjective bias when allocating animals/samples to treatment (e.g. **randomization procedure**)? If yes, have they been described?	Yes	Materials and Methods
Include a statement about **blinding** even if no blinding was done.	Yes	Materials and Methods
Describe **inclusion/exclusion criteria** if samples or animals were excluded from the analysis. Were the criteria pre-established?<br><br>If sample or data points were omitted from analysis, report if this was due to attrition or intentional exclusion and provide justification.	Not Applicable	
For every figure, are **statistical tests** justified as appropriate? Do the data meet the assumptions of the tests (e.g., normal distribution)? Describe any methods used to assess it. Is there an estimate of variation within each group of data? Is the variance similar between the groups that are being statistically compared?	Yes	Materials and Methods

Sample definition and in-laboratory replication	Information included in the manuscript?	In which section is the information available? (Reagents and Tools Table, Materials and Methods, Figures, Data Availability Section)
In the figure legends: state number of times the experiment was **replicated** in laboratory.	Yes	Figure legends
In the figure legends: define whether data describe **technical or biological replicates**.	Yes	Figure legends

Ethics

Ethics	Information included in the manuscript?	In which section is the information available? (Reagents and Tools Table, Materials and Methods, Figures, Data Availability Section)
Studies involving **human participants**: State details of **authority granting ethics approval** (IRB or equivalent committee(s), provide reference number for approval.	Yes	Material and Methods
Studies involving **human participants**: Include a statement confirming that **informed consent** was obtained from all subjects and that the experiments conformed to the principles set out in the WMA Declaration of Helsinki and the Department of Health and Human Services Belmont Report.	Yes	Material and Methods
Studies involving **human participants:** For publication of **patient photos**, include a statement confirming that consent to publish was obtained.	Not Applicable	
Studies involving experimental **animals**: State details of **authority granting ethics approval** (IRB or equivalent committee(s), provide reference number for approval. Include a statement of compliance with ethical regulations.	Yes	Material and Methods
Studies involving **specimen and field samples:** State if relevant **permits** obtained, provide details of authority approving study; if none were required, explain why.	Yes	Supplementary Materials

Dual Use Research of Concern (DURC)	Information included in the manuscript?	In which section is the information available? (Reagents and Tools Table, Materials and Methods, Figures, Data Availability Section)
Could your study fall under dual use research restrictions? Please check biosecurity documents and list of **select agents and toxins** (CDC): https://www.selectagents.gov/sat/list.htm	Not Applicable	
If you used a select agent, is the security level of the lab appropriate and reported in the manuscript?	Not Applicable	
If a study is subject to dual use research of concern regulations, is the name of the **authority granting approval and reference number** for the regulatory approval provided in the manuscript?	Not Applicable	

Reporting

The MDAR framework recommends adoption of discipline-specific guidelines, established and endorsed through community initiatives. Journals have their own policy about requiring specific guidelines and recommendations to complement MDAR.

Adherence to community standards	Information included in the manuscript?	In which section is the information available? (Reagents and Tools Table, Materials and Methods, Figures, Data Availability Section)
State if relevant guidelines or checklists (e.g., **ICMJE, MIBBI, ARRIVE, PRISMA**) have been followed or provided.	Not Applicable	
For **tumor marker prognostic studies,** we recommend that you follow the **REMARK** reporting guidelines (see link list at top right). See author guidelines, under 'Reporting Guidelines'. Please confirm you have followed these guidelines.	Not Applicable	
For **phase II and III randomized controlled trials**, please refer to the **CONSORT** flow diagram (see link list at top right) and submit the CONSORT checklist (see link list at top right) with your submission. See author guidelines, under 'Reporting Guidelines'. Please confirm you have submitted this list.	Not Applicable	

Data Availability

Data availability	Information included in the manuscript?	In which section is the information available? (Reagents and Tools Table, Materials and Methods, Figures, Data Availability Section)
Have **primary datasets** been deposited according to the journal's guidelines (see 'Data Deposition' section) and the respective accession numbers provided in the Data Availability Section?	Not Applicable	
Were **human clinical and genomic datasets** deposited in a public access-controlled repository in accordance to ethical obligations to the patients and to the applicable consent agreement?	Not Applicable	
Are **computational models** that are central and integral to a study available without restrictions in a machine-readable form? Were the relevant accession numbers or links provided?	Not Applicable	
If publicly available data were reused, provide the respective **data citations in the reference list**.	Not Applicable	
